# Identification, characterization and utilization of unigene derived microsatellite markers in tea (*Camellia sinensis *L.)

**DOI:** 10.1186/1471-2229-9-53

**Published:** 2009-05-11

**Authors:** Ram Kumar Sharma, Pankaj Bhardwaj, Rinu Negi, Trilochan Mohapatra, Paramvir Singh Ahuja

**Affiliations:** 1 Biotechnology Division, Institute of Himalayan Bioresource Technology, IHBT, (CSIR), Post Box 6, Palampur, Himachal Pradesh, 176061, India; 2 National Research Centre on Plant Biotechnology, Indian Agricultural Research Institute (IARI), New Delhi, 110012, India

## Abstract

**Background:**

Despite great advances in genomic technology observed in several crop species, the availability of molecular tools such as microsatellite markers has been limited in tea (*Camellia sinensis *L.). The development of microsatellite markers will have a major impact on genetic analysis, gene mapping and marker assisted breeding. Unigene derived microsatellite (UGMS) markers identified from publicly available sequence database have the advantage of assaying variation in the expressed component of the genome with unique identity and position. Therefore, they can serve as efficient and cost effective alternative markers in such species.

**Results:**

Considering the multiple advantages of UGMS markers, 1,223 unigenes were predicted from 2,181 expressed sequence tags (ESTs) of tea (*Camellia sinensis *L.). A total of 109 (8.9%) unigenes containing 120 SSRs were identified. SSR abundance was one in every 3.55 kb of EST sequences. The microsatellites mainly comprised of di (50.8%), tri (30.8%), tetra (6.6%), penta (7.5%) and few hexa (4.1%) nucleotide repeats. Among the dinucleotide repeats, (GA)n.(TC)n were most abundant (83.6%). Ninety six primer pairs could be designed form 83.5% of SSR containing unigenes. Of these, 61 (63.5%) primer pairs were experimentally validated and used to investigate the genetic diversity among the 34 accessions of different *Camellia *spp. Fifty one primer pairs (83.6%) were successfully cross transferred to the related species at various levels. Functional annotation of the unigenes containing SSRs was done through gene ontology (GO) characterization. Thirty six (60%) of them revealed significant sequence similarity with the known/putative proteins of *Arabidopsis thaliana*. Polymorphism information content (PIC) ranged from 0.018 to 0.972 with a mean value of 0.497. The average heterozygosity expected (*H*_*E*_) and observed (*H*_*o*_) obtained was 0.654 and 0.413 respectively, thereby suggesting highly heterogeneous nature of tea. Further, test for IAM and SMM models for the UGMS loci showed excess heterozygosity and did not show any bottleneck operating in the tea population.

**Conclusion:**

UGMS markers identified and characterized in this study provided insight about the abundance and distribution of SSR in the expressed genome of *C. sinensis*. The identification and validation of 61 new UGMS markers will not only help in intra and inter specific genetic diversity assessment but also be enriching limited microsatellite markers resource in tea. Further, the use of these markers would reduce the cost and facilitate the gene mapping and marker-aided selection in tea. Since, 36 of these UGMS markers correspond to the *Arabidopsis *protein sequence data with known functions will offer the opportunity to investigate the consequences of SSR polymorphism on gene functions.

## Background

The ubiquity of microsatellite or simple sequence repeats (SSRs) in eukaryotic genomes and their usefulness as genetic markers has been well established over the last decade. Microsatellites are mainly characterized by high frequency, co-dominance, multi-allelic nature, reproducibility, extensive genome coverage and ease of detection by polymerase chain reaction with unique primer pairs that flank the repeat motif [1]. As a result of these characteristics, microsatellites have become the most favoured genetic markers for plant breeding and genetics applications such as, assessment of genetic diversity, constructing framework genetic maps, mapping of useful genes, marker aided selection and comparative mapping studies [2,3].

In general, SSRs are identified from either genomic DNA or cDNA sequences. The standard method for development of SSR markers involves the creation of small insert genomic DNA libraries, followed by a subsequent DNA hybridization selection by probing them either with radioactively labeled probes or trapping them with biotinylated SSR motifs, and clone sequencing [4,5]. These processes are time consuming, and labour intensive. Furthermore, SSRs acquired by these methods are limited with probed SSR motifs (most common are di or tri types), and hence the advantages are partially offset. Availability and continuous enrichment of expressed sequence tags (ESTs) database  in most of the crop species can serve as an alternative strategy for identification and development of microsatellite markers. SSRs can be directly sourced from such databases, thereby reducing time and cost for microsatellite development. However, non-availability of sufficient sequence information (as generation of EST-SSR markers is primarily limited to those species and their close relatives for which large number of ESTs are available) and redundancy that yield multiple set of markers at the same locus are among the major drawbacks of EST derived microsatellite markers. More recently unique gene sequences (unigenes) have been developed *via *clustering of overlapping EST sequences, which overcomes the problem of redundancy in EST database and detect variation in the functional genome with unique identity and position [6]. Parida et al. [7] identified and characterized microsatellite motifs in the unigenes available in five cereal crops (rice, wheat, maize, sorghum, barley) and *Arabidopsis*. These unigene derived microsatellite (UGMS) markers are expected to possess high inter specific transferability as they belong to relatively conserved regions of the genome.

Tea is the oldest, widely consumed and least expensive natural beverage grown mostly in the tropical countries of Asia (India, Sri Lanka, China, Indonesia), Africa (Kenya, Uganda, Malawi) and to some extent Latin America (Argentina). Three *Camellia *species namely *C. sinensis *L. (small leaves), *C. assamica *(Masters; big leaf) and *C. assamica *ssp. *lasiocalyx *(Planchon ex Watt; intermediate leaf), traditionally referred as China, Assam and Cambod varieties, respectively are the important source of foreign exchange for almost all the tea producing countries in the world, including India. The complex life cycle and out breeding nature of tea poses several limitations for its genetic improvement through conventional breeding. The discrimination between true archetypal China, Assam and Cambod varieties is difficult due to heterogeneous nature of tea [8]. Furthermore, morphological characteristics are unable to reflect the inherent genetic variation within the crop, which actually shows high plasticity with respect to biochemical and physiochemical descriptors [9-12]. Therefore, identification of highly reliable molecular tools such as microsatellite or SSR markers is extremely important to reveal the unexplored genetic variation in tea. Despite the obvious advantages of microsatellite markers in terms of inferring allelic variation, estimating gene flow and development of genetic linkage maps [1], only a few microsatellite makers have been reported in tea [13-15]. Over the past few years, various EST projects and studies [16-18] have generated publicly available EST sequence data in tea. These ESTs were mostly derived from different organs/tissues such as, young & mature leaves and tender shoots under natural environmental conditions. Considering the multiple applications of such data in gene discovery and comparative genomics, publicly available EST sequence data (as on May 21, 2006) in *C. sinensis *was mined in the present study for SSR identification *via *clustering random ESTs into unigenes/contigs. These unigenes were also searched for abundance, repeat motif types and pattern of distribution of SSRs in the non-redundant (NR) expressed genome of tea. Functional analysis of unigenes containing SSRs was done through gene ontology (GO) annotations with the Arabidopsis information resource .

We report the development of UGMS primer pairs flanking these microsatellite motifs additional to those reported by Zhao et al. [15]. The UGMS markers developed were also tested for cross species transferability to different *Camellia *species. Locus orthology was monitored by analyzing the amplification patterns and by sequencing selected amplicons. Polymorphisms detected within the accessions of one species and between a set of *Camellia *species was also analyzed to assess as to whether these markers could be useful for diversity studies and also for distinguishing the *Camellia *species.

## Results

### ESTs/Unigenes data set

A total 1,223 (893 singletons and 330 contigs) unigenes were predicted from 2,181 publicly available EST database in *C. sinensis *by clustering of 2 – 34 random EST sequences. Non-redundant (NR) sequence data set represented ~425.67 kb expressed genome of tea (*C. sinensis*).

### Abundance and distribution of SSRs

All 1,223 potential unigenes were searched for the presence of microsatellites. A total of 109 (8.9%) unigenes containing 120 SSRs with motif length ranging from 2 to 6 bp were identified (Additional file 1). One sequence contained three SSRs and three sequences contained two SSRs each. Six SSRs were of compound types (SSR containing stretches of two or more different repeats). Of these, four compound SSRs were uninterrupted, while remaining two were interrupted by the presence of ≤ 8 arbitrary nucleotides. One SSR was detected for every 3.55 kb of the EST sequences. Further analysis of SSR containing unigene sequence data revealed that majority of them (94.1%) were perfect repeat and/or class I (≥20 nucleotides; nts length). However, remaining 5.8% (comprising of 2.5% di repeats and 0.83% each of tri repeats, tetra and penta repeats) were found to be of class II types (≥12 nts and <20 nts length).

Data analysis of SSR motifs in unigenes revealed 61 di repeats (50.8%), 37 tri repeats (30.8%), 8 tetra repeats (6.67%), 9 penta repeats (7.5%) and 5 hexa repeats (4.16%) (Table 1). Among the di-nucleotide repeats the (TC)n.(GA)n motifs were most abundant (83.6%) followed by (CA)n.(TG)n and (TA)n. Among the mirosatellites containing tri-repeats, (CAT)n.(ATG)n and (TTC)n.(GAA)n were the maximum (18.9%), which was followed by (TGG)n.(CCA)n and (CTG)n.(CAG)n. Abundance of other tri repeat containing SSRs were more or less in the similar range. Frequency of tetra, penta and hexa repeat containing SSRs was the least.

**Table 1:** Characteristics and frequency of different types of SSRs identified in 1223 unigenes of tea

S. No.	SSRs details						No. primers designed	Primers recorded successful amplification
			
	Repeat type	No.	Repeat motif units	No. of SSRs identified	Class I *	Class II **		
1.	Di-nucleotides	61	(TA)n	5	5		3	2
			(TC)n.(GA)n	51	48	3	47	29
			(CA)n.(TG)n	5	5		2	1
2.	Tri-nucleotides	37	(TTC)n.(GAA)n	7	6	1	6	5
			(TCC)n.(GGA)n	1	1		1	1
			(TCG)n.(CGA)n	3	3		1	1
			(CAT)n.(ATG)n	7	7		6	4
			(TGG)n.(CCA)n	6	6		4	4
			(CTG)n.(CAG)n	5	5		5	1
			(CCG)n.(CGG)n	3	2	1	2	1
			(TTA)n.(TAA)n	3	3		2	2
			(CAA)n.(TTG)n	2	2		2	2
4.	Tetra-nucleotides	8	(TATG)n.(CATA)n	2	2		1	-
			(TTTG)n.(CAAA)n	3	2	1	2	1
			(TTTC)n. (GAAA)n	1	1		1	1
			(TTGG)n.(CCAA)n	1	1		1	1
			(ACTG)n.(CAGT)n	1	1		0	-
5.	Penta-nucleotides	9	(TTCCC)n.(GGGAA)n	1	1		-	-
			(TTGTG)n.(CACAA)n	1	1		2	1
			(GAGAA)n.(TTCTC)n	2	2		1	1
			(TTTTA)n.(TAAAA)n	1	1		1	-
			(CAAGC)n.(GCTTG)n	1	1		1	-
			(GGAAA)n.(TTTCC)n	1	1		1	1
			(CGCTG)n.(CTGCG)n	1	0	1	1	-
			(TTCTC)n.(GTGAA)n	1	1		1	1
6.	Hexa-nucleotides	5	(GGGAGA)n.(TCTCCC)n	1	1		-	-
			(CCCTAA)n.(TTAGGG)n	1	1		0	-
			(TTTTTA)n.(TAAAAA)n	2	2		1	-
			(CAAAAA)n.(TTTTTG)n	1	1		1	1

	**Total**	**120**		**120**	**113**	**7**	**96**	**61**

* SSR with repeat length ≥ 20 nucleotides; nts,

** SSR with repeat length ≥ 12 nts to ≤ 20 nts.

### UGMS primer designation

Of the 109 NR unigenes containing one or more SSRs, 91 (83.5%) were amenable to design flanking oligonucleotide primer pairs. Ninety six UGMS primer pairs (55 from singletons and 41 from clusters) flanking to different repeat motifs could be designed. Primer pairs flanking di repeats (54.2%) were the most abundant followed by tri (30%), penta (8.3%), tetra (5.2%) and hexa (2.1%) repeats containing microsatellites. Primers could not be designed for the rest eighteen (16.5%) SSR containing unigenes because of either insufficient flanking sequence (occurrence of SSR near or/at either end of the unigene) or inability to fulfill the criteria for primer design. Five (4.6%) of the 109 unigenes were used to design more than one primer pairs targeting NR SSR loci. Thus, a non-redundant set of UGMS primers could be designed for 7.4% of the total unigene sequences in our study.

### Annotations and functional classification

**Figure 1:**
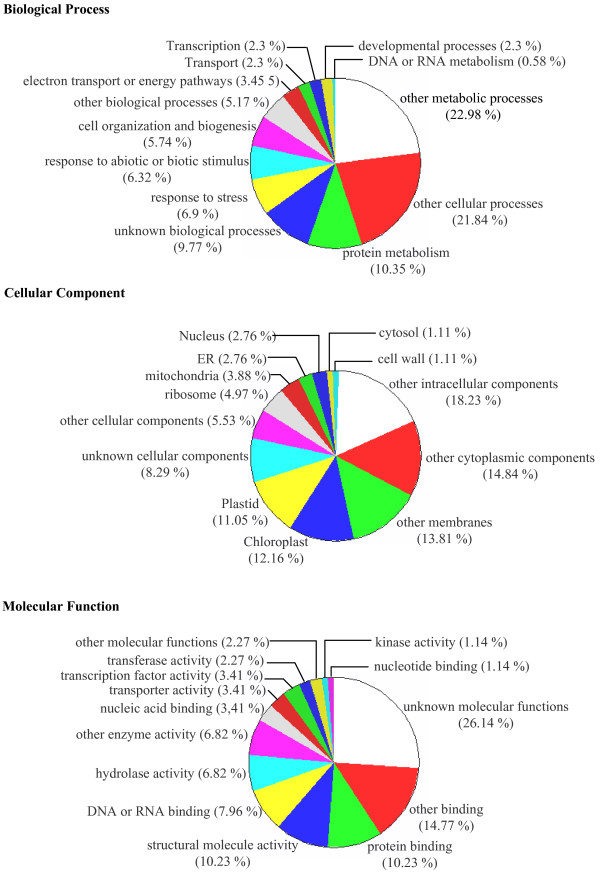
**Gene Ontology (GO) classification of the SSR containing tea unigenes**. The relative frequencies of GO hits for tea unigenes assigned to the GO functional categories biological processes, cellular components and molecular functions as defined for the *Arabidopsis *proteome.

Of the 60 unigenes that had successful primer pairs developed and validated, 36 (60%) matched to *Arabidopsis *genes with high expectation value (Table 2). To get a better view of the annotated unigenes, we downloaded Gene Ontology (GO) annotations [19] from the TAIR website [20] to classify SSRs containing unigenes into functional categories. Relative frequencies of GO hits for *C. sinensis *unigenes were assigned to the functional categories. Biological process, cellular components and molecular function as defined for *Arabidopsis *proteome are presented in Figure 1. In case of biological processes, the *C. sinensis *unigenes were assigned to thirteen categories. Majority were assigned to the two categories namely "other metabolic processes" (22.98%) and "other cellular processes" (21.84%). However, other important categories were "protein metabolism" (10.35%), "response to stress" (6.9%), "cell organization and biogenesis" (5.74%), etc. For the cellular components, the unigenes were assigned in thirteen categories with majority of them representing genes participating in "other intracellular components" (18.23%), "other cytoplasmic components" (14.84%) and "other membranes components" (13.8%). The remaining were assigned to important cellular components of "chloroplast" (12.16%), "ribosomes" (4.97%), "mitochondria" (3.88%), etc. When grouped according to likely molecular functions, the unigenes were assigned to fourteen categories and covered "protein binding" (10.23%), "other binding domains" (14.77%), "structural molecular activity" (10.23%), "various catalytic protein groups" (hydrolase, 6.8%; kinase, 1.14%) etc. There was considerable representation of unknown processes or fractions irrespective of the GO categories such as "unknown molecular functions" (26.14%), "unknown biological processes" (9.77%) and "unknown cellular components" (8.29%).

**Table 2:** List of 61 UGMS markers identified in a total of 60 unigenes showing the motif of the repeats unit and annotation of the unigenes as defined by best match *Arabidopsis *protein

Unigene ID	UGMS markers	Repeat motif	Arabidopsis Proteome hit
TUG1	TUGMS1	(TA)_13_	No hit
TUG3	TUGMS3	(TC)_10_	At5g25360-expressed protein; 1e-29
TUG4	TUGMS4	(TC)_11_(CA)_11_	At5g59320-Lipid transfer protein; 6e-24
TUG7	TUGMS7	(GA)_19_	No hit
TUG11	TUGMS11	(GA)_22_	At1g51650-Hydrogen ion transporting ATP synthase activity; 4e-31
TUG12	TUGMS12	(TA)_22_	At1g06680-calcium ion binding; 1e-20
TUG13	TUGMS13	(TG)_32_(TC)_24_	At5g26740-molecular function unknown; 1e-24
TUG15	TUGMS15	(GA)_14_	At5g10390-DNA binding; 1e-60
TUG17	TUGMS17	(TC)_25_	At3g22110-Ubiquitin-dependent protein; 5e-34
TUG18	TUGMS18	(GA)_10_	At4g05320-protein modification; 8e-39
TUG20	TUGMS20	(TC)_24_	At5g23860-Tubulin beta 8 chain; 2e-84
TUG22	TUGMS22	(GA)_13_	At2g14900-Gibberellin-regulated protein; 1e-05
TUG23	TUGMS23	(TC)_13_	At4g32130-UPF0480 family; 7e-42
TUG24	TUGMS24	(TC)_11_	At5g10390-histone H_3 _protein; 7e-40
TUG27	TUGMS27	(GA)_20_	At1g05010-1-amino cyclopropane-1-carboxylate oxidase; 7e-41
TUG28	TUGMS28	(TG)_12_(GA)_13_	At4g00165-lipid transport protein; 1e-30
TUG29	TUGMS29	(TC)_20_	At4g14420-Elicitor protein; 8e-10
TUG31	TUGMS31B	(TC)_9_	At1g33140-60S ribosomal protein; 8e-49
TUG33	TUGMS33	(GA)_10_	No hit
TUG34	TUGMS34	(TTC)_18_(GA)_10_	At4g25890-60S acidic ribosomal protein P_3_-1; 4e-13
TUG35	TUGMS35	(TC)_11_	At2g44650-Cholorplast chaperonin 10;; 3e-46
TUG36	TUGMS36	(GA)_13_	No hit
TUG41	TUGMS41	(GA)_11_	No hit
TUG42	TUGMS42	(GA)_23_(GA)_11_	No hit
TUG43	TUGMS43A	(GA)_11_	At2g18020-60S ribosomal protein; e-129
TUG44	TUGMS44	(GA)_12_	No hit
TUG45	TUGMS45	(GA)_20_	No hit
TUG46	TUGMS46	(TC)_13_	At4g10480-Alpha NAC putative; 1e-31
TUG48	TUGMS48	(GA)_14_	At1g19150-PSI type II chlorophyll a/b-binding protein, putative; 1e-10
TUG50	TUGMS50	(TC)_11_	At2g38140-30S ribosomal protein S31 choloroplast; 2e-07
TUG51	TUGMS51	(GA)_11_	At4g24820-26S proteosome non-ATPase regulatory subunit 6 probable; 3e-59
TUG52	TUGMS52	(GA)_14_	No hit
TUG58	TUGMS58A	(TCC)_14_	No hit
	TUGMS58B	(TCG)_28_	
TUG59	TUGMS59	(TGG)_9_	At3g49050-Calmodulin binding heat shock protein.; 2e-31
TUG63	TUGMS63	(CCG)_6_	At4g13940-Adenosylhomocysteinase 4e-29
TUG64	TUGMS64	(CAG)_9_	At3g26650-Glyceraldehyde-3-phosphate dehydrogenase A choloroplast precursor; 5e-38
TUG66	TUGMS66	(TTA)_8_	At5g03455-Dual specificity phosphatase cdc 25; 3e-17
TUG70	TUGMS70	(CAA)_15_	At4g34530-bHLH transcription factor; 3e-17
TUG71	TUGMS71	(GAA)_8_	At5g21430-DnaJ domain family; 5e-43
TUG72	TUGMS72	(ATG)_10_	At5g57660-Zinc finger protein; 7e-23
TUG73	TUGMS73	(TAA)_12_	No hit
TUG74	TUGMS74	(CCA)_9_	No hit
TUG75	TUGMS75	(ATG)_9_	No hit
TUG76	TUGMS76	(GAA)_10_	No hit
TUG77	TUGMS77	(CAA)_10_	No hit
TUG78	TUGMS78	(TCC)_13_	No hit
TUG79	TUGMS79	(CCA)_11_	At2g35960-hairpin induced protein putative; 1e-27
TUG82	TUGMS82	(CAT)_8_	At28750-Photosystem I subunit putative; 8e-09
TUG83	TUGMS83	(TGG)_9_	At4g13850-Glycine rich RNA binding protein 6e-34
TUG84	TUGMS84	(ATG)_34_	No hit
TUG85	TUGMS85	(GAA)_11_	No hit
TUG87	TUGMS87	(GAA)_6_	No hit
TUG90	TUGMS90	(TTTG)_6_	No hit
TUG92	TUGMS92	(TTTC)_13_	At1g49410-mitochondrial import receptor subunit TOM6 homolog; 2e-08
TUG95	TUGMS95	(CAAA)_6_	No hit
TUG98	TUGMS98	(TTGTG)_8_	No hit
TUG99	TUGMS99	(GGGAGA)_7_(GAGAA)_6_	At5g01650-light inducible protein; 1e-49
TUG102	TUGMS102A	(GGAAA)_12_(GA)_11_	No hit
TUG105	TUGMS105	(TTCTC)_5_	No hit
TUG108	TUGMS108	(CAAAAA)_6_	At1909310-expressed; 7e-11

In general, the SSRs containing unigene sequences detected in tea were homologous to proteins having distinct molecular functions such as, binding, catalytic, transport, enzyme regulators, and structural activities in different biological processes, and cellular and sub-cellular organization.

### Marker evaluation and polymorphism detection

Ninety six primer pairs designed in this study were used to amplify DNA from a panel of 34 accessions of cultivated tea and related species. Of these, 61 (63.5%) primer pairs produced repeatable and reliable amplifications in at least four accessions of tea, while 35 (36.5%) primer pairs either completely failed or led to weak amplifications and thus were excluded from further analysis. Marker evaluation details are given in Table 3. PCR products of the expected size were obtained in all the cases except in one UGMS primer (TUGMS83) that had amplified larger size additional amplicons in some cases. Multi-locus amplifications were recorded in case of TUGMS27 and TUGMS46. Over all, amplification success rate was the maximum in case of TUGMS primer pairs containing tri repeats (72%), followed by di-repeat (61.5%). The PCR success rate of UGMS classes having tetra, penta and hexa repeats were ranged from 50% to 60%. Seven polymorphic primer pairs namely TUGMS3, TUGMS7, TUGMS33, TUGMS46, TUGMS52, TUGMS75, TUGMS85 gave amplification in all the tested genotypes irrespective of species (Table 3) and hence can be utilized as universal markers for molecular analysis in tea. However, these markers need to be validated in a larger panel of *Camellia *species.

**Table 3:** Marker validation and features of new 61 UGMS markers of tea

					Heterozygosity **				
								
Locus name *	Primer sequence	Repeat Motif	Annealing temperature (*T*_*a*_)	No. of alleles	*H*_*O*_	*H*_*E*_	PIC	Approximate size range (bp)	No. of genotypes amplified
TUGMS1	F5’CTTCAAGTTGAGTTTGTCCG' R5'CAAGGGATGGTTTTCACTTG	(TA)_13_	55°C	4	0.118	0.558	0.770	85 bp–100 bp	15
TUGMS3	F5'GCGTATGGAAAAGCTGAGAA3' R5'GAAGCAAACCACTGAGGTGA3'	(TC)_10_	57°C	8	0.559	0.857	0.595	160 bp–220 bp	34
TUGMS4	F5'CCACCGACTCGATGACATAA3' R5'GCATTGAGATTGATGGACCA3'	(TC)_11_(CA)_11_	57°C	6	0.765	0.808	0.306	250 bp–300 bp	32
TUGMS7	F5'GGACCACTTGATTTTCAGCT3' R5'ACGTACAATCACCACCGACT3'	(GA)_19_	55°C	6	0.853	0.766	0.154	300 bp–400 bp	34
TUGMS11	F5'GGGGAGTGTTTGTTTGAATA3' R5'TGTAGGGTTCTTTGAGGCAG3'	(GA)_22_	55°C	8	0.500	0.857	0.630	190 bp–240 bp	29
TUGMS12	F5'GAAGTTTGTTGAGAGTGCTGC3' R5'ACAGATCTAAATTTGGGGGG3'	(TA)_22_	55°C	7	0.382	0.663	0.294	160 bp–200 bp	30
TUGMS13	F5'GATCTGTGTCTCTCTGTTCCC3' R5'CCACACATCATCTTTTCCTC3'	(TG)_32_(TC)_24_	55°C	7	0.324	0.804	0.675	185 bp–205 bp	25
TUGMS15	F5'GTTGCTTCCTTGGTGCCT3' R5'GCGGGGACCACATYCAGTA3'	(GA)_14_	55°C	15	0.500	0.871	0.692	145 bp–190 bp	30
TUGMS17	F5'GGGGAATTTCAGACAGACAC3' R5'GCCGTTCAGTGTAGTAGATCG3'	(TC)_25_	55°C	5	0.588	0.796	0.414	160 bp–200 bp	25
TUGMS18	F5'GGGGAAGAAAAAAAAAGTTG3' R5'TTTCTGGATGTTGTAGTCGG3'	(GA)_10_	55°C	4	0.059	0.583	0.921	190 bp–260 bp	13
TUGMS20	F5'GGGGAATTTCATCACTCAAAC3' R5'AGATCGGAGTCACCGTTGTA3'	(TC)_24_	55°C	4	0.235	0.748	0.727	290 bp–320 bp	21
TUGMS22	F5'GGCAGCTTCAGTTCATCTCT3' R5'CATAAGGAAAGCTGCAAGAG3'	(GA)_13_	55°C	8	0.559	0.835	0.626	140 bp–160 bp	24
TUGMS23	F5'GGGGAGCTTACAAAGAGTCA3' R5'GTGCCGAAGAGAGGATAGAG3'	(TC)_13_	55°C	8	0.618	0.839	0.503	135 bp–200 bp	31
TUGMS24	F5'CTCACTACAGCRGCAACCGC3' R5'CCTGAATCTAGTGGGGCTTC3'	(TC)_11_	55°C	5	0.235	0.727	0.640	280 bp–300 bp	24
TUGMS27***	F5'GGGGATAGTACAAACACACAAC' R5'GCTCCTCTTTCTTCACCACTT'	(GA)_20_	55°C	9	-	-	-	80 bp–110 bp	32
TUGMS28	F5' GTCCCCATTGCTCTTAGTTT 3' R5' GACAATCATTGCCACCACAT 3'	(TG)_12_ (GA)_13_	55°C	4	0.529	0.745	0.384	170 bp–200 bp	29
TUGMS29	F5' CAAAACAGAGCCTTCATAAG 3' R5' ATCGAGACAGAAGACAGACG 3'	(TC)_20_	53°C	4	0.029	0.481	0.968	105 bp–120 bp	10
TUGMS31B	F5' CTATGTACGACTCTCTGCCTG3' R5' GTTTGTCTGGAGTTAAACGAG3'	(TC)_9_	55°C	5	0.294	0.558	0.853	140 bp–170 bp	12
TUGMS33	F5'CCCTCTTCTCTCACCAGATC3' R5'TCCCTTCTTTGCCTTCTACA3'	(GA)_10_	55°C	3	0.441	0.615	0.180	150 bp–160 bp	34
TUGMS34	F5'GCCAAAATTCCATCTAGGG3' R5'TGCAACTCGTATGTGGACC3'	(TTC)_18_(GA)_10_	55°C	10	0.618	0.828	0.364	160 bp–200 bp	32
TUGMS35	F5'GGGGCTCTCTCTCTCTAAAG3' R5'TGCTGTGAGAAGTAAAGGGC3'	(TC)_11_	55°C	6	0.853	0.848	0.267	110 bp–140 bp	30
TUGMS36	F5'GCCAGCAAGTAAGAGAAGCT3' R5'GTGGGTTGAGTACCAACAGG3'	(GA)_13_	55°C	2	0.029	0.510	0.856	125 bp–115 bp	13
TUGMS41	F5'CCTTTCACAACAGATCCACA3' R5'GAGCTTCCTGACGATGGTTA3'	(GA)_11_	55°C	3	0.235	0.625	0.717	115 bp–120 bp	16
TUGMS42	F5'GTAGCTCGCAACACAACACC3' R5'CTCCAACGACACACTCTCTG3'	(GA)_23_ (GA)_11_	55°C	7	0.588	0.860	0.581	100 bp–180 bp	29
TUGMS43A	F5'CATTTCCTTCTCACCCCTAC3' R5'GTGGGTGTGGGACTTGAATA3'	(GA)_11_	55°C	4	0.264	0.576	0.842	150 bp–170 bp	13
TUGMS44	F5'GTGTTGGGAGTGTTGCTGAA3' R5'ACCACCTGATTCGACATCTC3'	(GA)_12_	55°C	4	0.118	0.478	0.353	300 bp–360 bp	25
TUGMS45	F5'GGGGATTGTTGAAGTTTCTC3' R5'CTTCACCCATATCTTCCAAA3'	(GA)_20_	55°C	2	0.118	0.555	0.764	148 bp–150 bp	16
TUGMS46***	F5'GGGTTCAGTCGCAGCAAA3' R5'GAGGAGTTCTTCTTGCGTCT3'	(TC)_13_	55°C	10	-	-	-	98 bp–120 bp	34
TUGMS48	F5'TCGGGCAACCACCATATATA3' R5'CTTTTCCCACCAGACAAGAA3'	(GA)_14_	55°C	7	0.441	0.880	0.755	100 bp–135 bp	28
TUGMS50	F5'GGGGATTCATCTCTGAACAC3' R5'GGAGAGAGTGAGAGCTTTGG3'	(TC)_11_	55°C	2	0.059	0.527	0.932	168 bp–175 bp	12
TUGMS51	F5'CCAGACTCATCGCAGAAATC3' R5'GGTTGGGTGAGGAGGAATAG3'	(GA)_11_	55°C	7	0.765	0.792	0.353	145 bp–170 bp	32
TUGMS52	F5'GAACCAACCCAGTCTATACTCC3' R5'AGCACACGCCATCCAATC3'	(GA)_14_	55°C	16	0.794	0.909	0.622	90 bp–120 bp	34
TUGMS58A	F5'TTCTTCCTCTTCTTTGGTGG3' R5'AGAGGGTGAAGAGGAAGTTG3'	(TCC)_14_	55°C	4	0.647	0.678	0.210	90 bp–110 bp	31
TUGMS58B	F5'CAACTTCCTCTTCACCCTCT3' R5'GCTGAAGAGAACGGTGAAGA3'	(TCG)_28_	55°C	2	0.059	0.216	0.124	140 bp–160 bp	31
TUGMS59	F5'CACCTTCATCTTCACCTTCC3' R5'TGAGTCTGCTCGTAGGTGAG3'	(TGG)_9_	55°C	3	0.382	0.454	0.018	168 bp–180 bp	25
TUGMS63	F5'CAAGGTAAAGGACATGCACC3' R5'GTCCTCAGAAGCCATCGAA3'	(CCG)_6_	55°C	2	0.177	0.613	0.568	150 bp–155 bp	22
TUGMS64	F5'TGCAGGGGAGATGAATTAAC3' R5'ACCTGCATTTCCCAGTCTT3'	(CAG)_9_	55°C	5	0.382	0.775	0.605	280 bp–320 bp	24
TUGMS66	F5'AATGGTTGGGTAAGCCTCT3' R5'TGACCAACAACGGATCACA3'	(TTA)_8_	55°C	4	0.441	0.644	0.231	220 bp–320 bp	29
TUGMS70	F5'ATCAGACGATGTACCGAAGAG3' R5'CGAACGTGAATGTAATCAGG3'	(CAA)_15_	55°C	2	0.029	0.504	0.782	180 bp–190 bp	14
TUGMS71	F5'AGCAGCAAGTGTCGTTTACA3' R5'GCAGAAATGAGAGAAGGAGG3'	(GAA)_8_	55°C	3	0.235	0.511	0.204	240 bp–320 bp	30
TUGMS72	F5'CCAGCTCGATAGCATCTACA3' R5'CACTATCCAAATCCATCGC3'	(ATG)_10_	55°C	2	0.559	0.396	0.072	198 bp–205 bp	28
TUGMS73	F5'GTCAAGACGCCCACTACAGT3' R5'GACTGTGTAACCTGCCAAGAC3'	(TAA)_12_	55°C	9	0.677	0.907	0.694	150 bp–220 bp	32
TUGMS74	F5'CACCCCCTTCCTATTCAAA3' R5'AGGTGGTCACTTCTTCAACG3'	(CCA)_9_	55°C	6	0.706	0.900	0.676	170 bp–200 bp	32
TUGMS75	F5'GGTGATCCGATGGTGAATT3' R5'ACAGGAGCATCAACAGCAGG3'	(ATG)_9_	55°C	4	0.265	0.293	0.032	240 bp–280 bp	34
TUGMS76	F5'AGATGAGCACAAGGAAGGAG3' R5'CGAAGTAGTGTAGGGGAAGAA3'	(GAA)_10_	55°C	2	0.441	0.618	0.652	198 bp–210 bp	16
TUGMS77	F5'CTACCCTTCTTCTCAGTTCCA3' R5'CAGATGAAATGAAGGGCATC3'	(CAA)_10_	55°C	2	0.206	0.490	0.848	132 bp–140 bp	11
TUGMS78	F5'CACCGCTTGACTAAAATGG3' R5'AAACTATCAACCGTATGGGC3'	(TTC)_13_	55°C	8	0.647	0.878	0.597	130 bp–170 bp	31
TUGMS79	F5'GGGTAATTTAAGGGTGTCCT3' R5'AAGAGGGTGATAAGGATTCC3'	(CCA)_11_	55°C	7	0.324	0.682	0.503	160 bp–260 bp	24
TUGMS82	F5'AAGTTAGAGAGAGAGAAGTGGC3' R5'AATGCCACACCAGTCCTAG3'	(CAT)_8_	55°C	6	0.412	0.691	0.344	140 bp–180 bp	30
TUGMS83	F5'GAGGATTTGGGTTTGTGAAC3' R5'TCATTCTCTCTGGCATCACC3'	(TGG)_9_	55°C	4	0.765	0.671	0.242	250 bp–600 bp	30
TUGMS84	F5'GCTAGGCATTCGAGGAGTT3' R5'GGACTCCTCACTGCTTGAAG3'	(ATG)_34_	55°C	2	0.500	0.499	0.060	220 bp–500 bp	31
TUGMS85	F5'GACGGAAAATCGAAGGC3' R5'TCTTACTGCTCTTGGCTTCC3'	(GAA)_11_	55°C	3	0.059	0.140	0.063	120 bp–140 bp	34
TUGMS87	F5'TCACATTTTCAGAGGAAGAGG3' R5'TTAGGGTTTAGTTGTGGCTG3'	(GAA)_6_	55°C	5	0.235	0.733	0.673	90 bp–125 bp	28
TUGMS90	F5'GAGGGGAAGTGTGAAAAATC3' R5'TTGGGATTCCTTTTCTATGC3'	(TTTG)_6_	55°C	4	0.412	0.696	0.607	95 bp–120 bp	18
TUGMS92	F5'TCTATCAGTTGGCTTGGTTG3' R5'CAATCTTTCACTGGCATGAG3'	(TTTC)_13_	55°C	4	0.118	0.269	0.972	145 bp–180 bp	5
TUGMS95	F55'GGCTCCTTCCTCTTCTGATC3' R5'TGAAGTTGGGATTGAGCATG3'	(CAAA)_6_	55°C	5	0.412	0.777	0.596	120 bp–150 bp	23
TUGMS98	F5'AGCCCAACTCCTCCTGAC3' R55'GAGCAGCCTCATTCGGAC3'	(TTGTG)_8_	55°C	3	0.441	0.613	0.223	280 bp–380 bp	31
TUGMS99	F5'GAATAGGGTTTGGCAGAGGC3' R5'AGGATGGAGGAGGTGTCAA3'	(GGGAGA)_7_ (GAGAA)_6_	55°C	6	0.206	0.154	0.542	160 bp–200 bp	25
TUGMS102A	F5'CGTAGCTCGCACACAACAC3' R5'CGTCCCCTCCGAAATGA3'	(GGAAA)_12_	55°C	6	0.706	0.803	0.230	100 bp–120 bp	27
TUGMS105	F5'GGGAGCTAGGGTTTTAGTTT3' R5'CTTCAGAGCCACTTCTTTGTC3'	(TTCTC)_5_	55°C	5	0.618	0.711	0.098	150 bp–200 bp	30
TUGMS108	F5'GGGACATCATCACCAGCTT3' R5'TTCCTTGGTAGAACTCTGCTT3'	(CAAAAA)_6_	55°C	6	0.853	0.790	0.141	130 bp–160 bp	30

TUGMS,Tea unigene microsatellite; bp, base pair; H_*O*_, Observed heterozygosities; H_*E*_, Expected heterozygosities)

* Accession details of contributing ESTs for unigenes are given in additional file (see Additional file 1).

**A significant deviation in Hardy-Weinberg equilibrium at P < 0.001 level recorded all single locus UGMS primers.

***TGUMS marker with multi locus amplifications.

**Figure 2:**
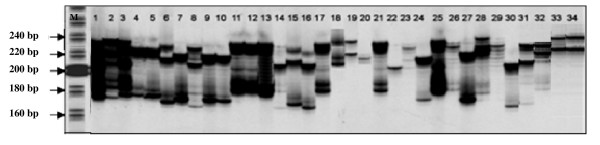
**PCR amplification profile generated with primer TUGMS3**. Lanes 1–34 represent accessions of *Camellia *spp. as presented in Table 6; M: 20 bp DNA ladder (Cambrex bioproduct, USA) as size standards.

Sixty one primer pairs amplified 324 alleles of which 321 (99%) were found to be polymorphic. All the UGMS markers identified in the present study remained highly polymorphic (Figure 2). The number of alleles detected in the present case ranged from 2 to 16 with an average of 5.3. The UGMS markers namely TUGMS52 and TUGMS15 recorded a maximum of 16 and 15 alleles, respectively. Total number of alleles detected among the accessions belonging to three varietal types i.e. Assam, Cambod and China were 213, 214 & 278, respectively. A high level of polymorphism has been observed at the species level. No significant difference was detected in percentage polymorphism of China and Assam (~94% in each case), however, due to hybrid nature of *C. assamica *ssp. *lasiocalyx*, a slightly higher level of polymorphism (98.4%) was recorded in Cambod. The *H*_*E *_and *H*_*o *_ranged from 0.140 to 0.909 (with an average of 0.654) and 0.029 to 0.853 (with an average of 0.413), respectively (Table 3). All the UGMS markers showed a significant departure from Hardy-Weinberg equilibrium (HWE) at P < 0.001 level. The polymorphism information content (PIC) ranged from 0.018 to 0.972 with an average of 0.497. There was significant difference in the average PIC values was recorded in UGMS locus harboring different repeat types. Average PIC values ranged from 0.183 (penta repeats) to 0.725 (tetra repeats). However, an average of 0.578 and 0.390 PIC values were recorded in TUGMS primers with di and tri repeats, respectively (Table 3). Of the 34 UGMS primer pairs with PIC values ≥ 0.50, 5 (13.8%) namely TUGMS3, TUGMS52, TUGMS73, TUGMS74, TUGMS78 recorded amplification in ≥30 accessions were identified as informative and thus would be useful in future marker assisted studies in tea. Further, at least 14 primer pairs with PIC values ≥ 0.70 were identified, which may also be categorized as informative primers after their validation in a larger panel of tea accessions.

In mutation drift equilibrium, heterozygosity excess/deficiency under different mutation models (IAM & SMM) generated by BOTTLENECK showed significant excess of heterozygosity in both the models. All the tested loci showed excess heterozygosity in sign test and found to be significant in both standardized and Wilcoxen test (Table 4).

**Table 4:** Allele frequency based mutation drift equilibrium of UGMS loci

Mutation model	Sign test	Standardized differences test	Wilcoxon test
IAM	Hee = 20.49	T_2 _= 13.196	P (one tail for H deficiency) 1.000
	Hd = 0	P = 0.000	P (one tail for H excess) 0.000
	He = 40		P (two tails for H excess and deficiency) 0.000
	P = 0.000		
			
SMM	Hee = 22.40	T_2 _= 11.518	P (one tail for H deficiency) 1.000
	Hd = 0	P = 0.000	P (one tail for H excess) 0.000
	He = 40		P (two tails for H excess and deficiency) 0.000
	P = 0.000		

(IAM, Infinite allele model; SMM, Stepwise mutation model; Hee = Expected heterozygosity excess; Hd = Heterozygosity deficiency; He = Heterozygosity excess)

### Cross-species transferability

To assess the conservation of *C. sinensis *UGMS loci across the *Camellia *species, we tested the cross amplification of 61 primer pairs on five species representing ten accessions each of C. *assamica *and *C. assamica *ssp. *lasiocalyx *(cultivated tea) and one accession each representing *C. lutescens, C. irrawadiensis, C. japonica *white flower and *C. japonica *red flower (wild and/or ornamental species). Except for the annealing temperature (*Ta*), identical PCR conditions were used to assess the extent of transferability to related species. All the 61 primers recorded transferability in *C. assamica *and *C. assamica *ssp. *lasiocalyx *showing high degree of locus conservation in the cultivated species. However, 51 UGMS primers gave reproducible amplification at least in a single related species (*C. lutescens*; 63.4%, *C. irrawadiensis*; 34.4%, *C. japonica*; red; 59% and white flower; 57.4%) and recorded an overall 83.6% cross transferability rate. Marker wise amplification pattern of successful UGMS primers is presented in Table 5. Furthermore, transferability rate was significantly higher in TUGMS primers containing tri or hexa repeats (≥ 95%) followed by the primers with di and penta repeats (75% in each case). Least transferability was recorded in primers with tetra repeats. As a whole, 15 (~25%) UGMS primers recorded cross-transferability in all the tested species.

**Table 5:** Cross-species amplification pattern of tea UGMS markers

S. No.	Name of locus	*C. irrawadiensis*	*C. lutescens*	*C. japonica, (*Red flower)	*C. japonica *(White flower)
1	TUGMS3	+	+	+	+
2	TUGMS4	+	+	-	-
3	TUGMS7	+	+	+	+
4	TUGMS11	+	-	+	-
5	TUGMS12	+	-	+	-
6	TUGMS15	-	-	+	+
7	TUGMS22	-	+	-	-
8	TUGMS23	-	+	+	+
9	TUGMS24	-	-	-	+
10	TUGMS27	+	+	-	-
11	TUGMS28	+	+	+	-
12	TUGMS29	-	-	+	-
13	TUGMS33	+	-	+	+
14	TUGMS34	+	+	+	+
15	TUGMS35	-	+	-	-
16	TUGMS36	+	-	-	-
17	TUGMS42	+	-	+	+
18	TUGMS43A	-	-	+	-
19	TUGMS44	+	-	-	-
20	TUGMS45	+	-	-	+
21	TUGMS46	+	+	+	+
22	TUGMS48	+	+	+	+
23	TUGMS50	-	-	+	+
24	TUGMS51	+	+	+	+
25	TUGMS52	+	+	+	+
26	TUGMS58A	+	+	+	+
27	TUGMS58B	+	-	+	+
28	TUGMS59	-	+	+	+
29	TUGMS63	+	-	-	+
30	TUGMS64	+	-	-	+
31	TUGMS66	-	-	-	+
32	TUGMS70	+	-	-	-
33	TUGMS71	+	-	+	+
34	TUGMS72	+	-	+	-
35	TUGMS73	+	+	+	+
36	TUGMS74	+	+	+	+
37	TUGMS75	+	+	+	+
38	TUGMS76	+	-	+	+
39	TUGMS77	+	-	-	-
40	TUGMS78	+	+	+	+
41	TUGMS79	+	-	+	+
42	TUGMS82	+	-	+	+
43	TUGMS83	+	-	+	+
44	TUGMS84	+	+	+	+
45	TUGMS85	+	+	+	+
46	TUGMS87	+	-	+	+
47	TUGMS90	+	-	-	-
48	TUGMS98	+	-	+	+
49	TUGMS99	+	+	+	+
50	TUGMS102A	-	-	+	+
51	TUGMS108	-	+	-	-

Overall Transferability		39 (63.4%)	23 (34.4%)	36 (59.0%)	35 (57.4%)

### Sequence comparison of SSR locus

**Figure 3:**
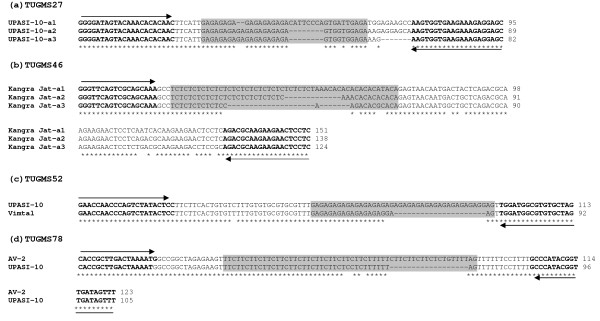
**Sequence alignment of different amplicons**. Different amplicons from the same accessions are indicated by the name of accessions followed by a1, a2 and a3, with primers TUGMS27 (a) and TUGMS46 (b). Alleles from the different accessions are indicated by their names, with primers TUGMS52 (c) and TUGMS78 (d). The shaded nucleotide highlights the microsatellite motifs and arrow indicate the primer sequences used to amplify the microsatellites in each case.

**Figure 4:**
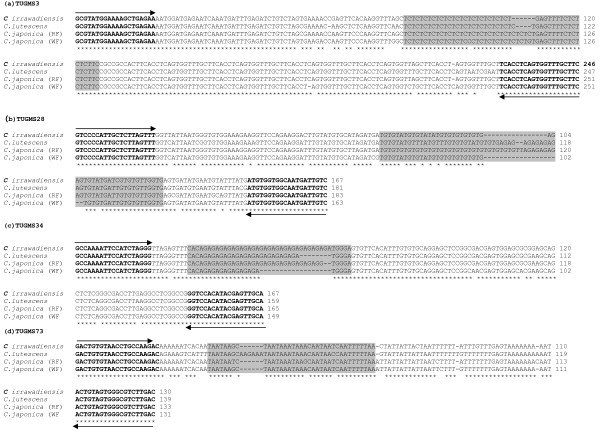
**Sequence alignment of cross species amplicons**. Cross-species amplicons obtained with TUGMS3, TUGMS28, TUGMS34, TUGMS73 markers in different *Camellia *spp. are indicated by species names. The shaded nucleotide highlights the conservation of microsatellite motifs in different species and arrow indicates the respective primer sequences.

To validate the conservation of SSRs across the varieties and species, at least one amplicon from different genotypes/species and multiple amplicons from the same genotypes were sequenced. Multiple amplicons from single genotype were selected to determine the orthology and paralogy of the sequence. When a locus wise DNA sequences data in each case was compared, it showed electromorphic size variation solely attributed either due to expansion/contraction of the SSRs, or due to interruptions in the SSR regions. This was most notable among different alleles where the size differences resulted from either simple or complex variation in SSR motifs. Even at the multiple amplicons from the diploid genotypes similar situation was noticed. As illustrated in Figure 3, the size of the multiple amplicons having (GA)n motif and consumed primer sites were 95, 89, 82 bp longer in case of genotype UPASI-10 for marker TUGMS27. Similarly, for the Kangra Jat genotype amplicon size 124, 138 and 151 bp were obtained for TUGMS46 that amplified TC repeats. Similar situation was observed with identical amplicon size, and repeat motifs for allelic amplicons from different genotypes as in case of TUGMS3 and TUGMS53, respectively. Further, in order to confirm DNA polymorphism and cross-transferability at the sequence level, selected amplicons from C. *lutescens, C. irrawadiensis *and *C. japonica *(RF: red flower & WF; white flower) were sequenced for three UGMS primers namely TUGMS3, TUGMS-34 and 73. The presence of the target microsatellites were observed in all the cases (Figure 4).

### Inter and intra specific genetic variations among the tea accessions

In the present study, correlations observed between the genetic similarity (GS) matrixes based on Jaccard's and Nei and Li's coefficients methods was 0.991. The average GS among the 34 accessions of *Camellia *species was 22%. Within *C. sinensis*, GS ranged from 26% between Kangra Jat and Sikkim-1 to 59% between Teesta Valley-1 and Sikkim-1. Within *C. assamica *GS was ranging from 15% to 71%, where as GS ranged from 26% to 46% in *C. assamica *ssp. *lasiocalyx*. The average GS remained almost similar in case of *C. assamica *(Assam; 28.6%) and *C. assamica *ssp. *lasiocalyx *(Cambod; 28%), while slightly less among the accessions of *C. sinensis *(China; 27%). We recorded 37% GS between the two accessions of ornamental types *C. japonica *with red and white flowers.

### Cluster analysis

**Figure 5:**
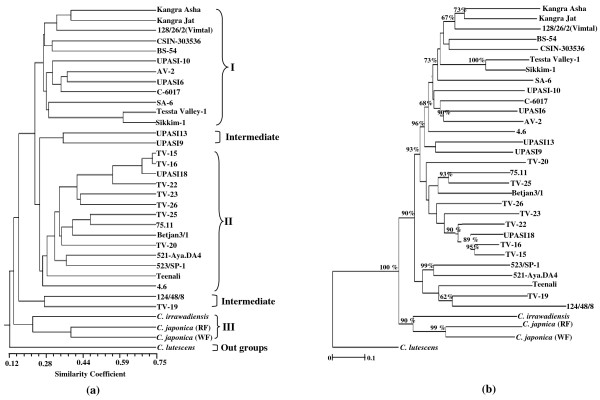
**Phylogenetic tree construction**. Genetic relationships among 34 accessions of *Camellia *spp. based on the 61 UGMS primers identified in the present study.(a) UPGMA clustering based on Jaccard's coefficient of similarity; (b) Neighbour Joining tree based on Nei and Li distance. Tree branched with bootstrap values greater than 60% are indicated. The scale bar represents simple matching distance.

The phenetic analysis of the UGMS data by two methods showed distinct groups and subgroups (Figure 5a &5b). The cluster analysis with Jaccard's similarity matrix corresponded well with the Nei and Li's matrix. Though minor changes were evident within the subclusters of the major varietal types, the relative position of the major clusters remained preserved. The neighbour joining (NJ) tree was more precise in differentiating the closely related accessions with high bootstrap values (Figure 5b). Clustering of thirty four accessions of genus *Camellia *into three major groups was strongly supported by high bootstrap values (≥ 90%). However, accession of *C. lutescens *remained isolated as a single solitary genotype with 100% bootstrap value and defined as outgroup. All the China accessions were clustered together in group I. However, two accessions namely UPASI 6 (Assam) and C-6017 (Cambod) were also clustered in this group. Majority of Assam and Cambod tea accession clustered together in group II with bootstrap values of 65%. All but one (TV-19), TV series accessions representing either Assam or Cambod also clustered together in group II. Interestingly, two accessions namely UPASI 13 and UPASI 9 known for excellent spread and are the source of good quality tea, remained together as intermediates between groups I and II. Accession 124/48/8, an extreme Cambod type with broad-elliptic leaves without distinct marginal veins with pink pigmentation at the petiole base, along with TV-19 (Cambod) clustered as an intermediate group between ornamentals and cultivated tea accessions. As expected, all the three species (*C. irrawadiensis*, *C. lutescens, C. japonica *with white and red flower) clustered separately in the present case.

## Discussion

### Abundance and distribution of SSRs and UGMS primer development

The present study was designed to utilize the publicly available tea ESTs for development of reliable UGMS markers. We assembled ESTs into unigenes, consisting of consensus sequences of contigs and the singleton sequences for SSR analysis. The assembly generates longer sequences, which gives a better chance of association of sequences with the proteins. Generation of longer sequences can be useful for SSR studies since it can give longer SSR surrounding sequences for primer designing. In addition, the use of NR sequences can give a better estimation of the sequence features in the genome.

In case of tea, we found that 8.9% unigenes contained NR SSRs. This EST-SSR frequency was in the 2.65 – 10.62% range obtained for 49 dicot species [21]. However, it was higher than the 1.5 – 4.7% range reported for monocots [22]. Frequency of EST-SSRs in various plant genomes is significantly influenced by the repeat length and the criteria used to search the SSRs in database mining [23]. If the repeat length is 20 bp, in general 5% of ESTs have recorded the presence of microsatellites [6]. The present study recorded a relatively higher abundance of SSRs as compared to earlier reports in tea [15] and also in other plant species such as grapes [24], sugarcane [25], cereals [7,22,26] and coffee ESTs [23,27]. Cardle et al. [28] in a comprehensive computational and experimental characterization of publicly available EST sequence database of different plant genomes recorded a significant difference in the type and abundance of SSRs. The average distribution of SSRs estimated to be ranging from 3.4 kb in rice to 7.4 kb in soybean, 8.1 kb in maize, 11.1 kb in tomato, 13.8 in *Arabidopsis*, 14.0 kb in popular and 20 kb in cotton. Furthermore, occurrence of high frequency of Class I (94.1%) and or perfect repeats in the present case is possibly due to the criteria that had been implemented for mining of SSRs. Experimental data originally reported for human [29] and then confirmed in many other organisms including rice [30,31] had suggested that longer perfect repeats are more polymorphic. The rate of strand slippage has been shown to increase with increasing length of blocks of repeats. Therefore, longer perfect repeats are highly variable. However, the lower rate of polymorphism of repeat sequences containing interruptions may be due to the fact that strand slippage of these sequences produces structures with non-complementary bases.

The frequency analysis of various nucleotide repeats in *C. sinensis *ESTs revealed that di nucleotide SSRs were the most abundant SSRs followed by tri-, tetra-, penta- and hexa repeats. This is in agreement with the frequency trend has been earlier reported in tea [15]. In general, microsatellites containing tri-repeats remained most common among the monocots and dicots [6]. However, Kumpata and Mukhopadhyay [21] recorded the abundance of di-repeats in most of the dicots species investigated. High frequency of di-nucleotide repeat has also been reported in case of eucalyptus [32] and citrus [33] ESTs. High frequency of dinucleotide repeats as observed in the present case could be because ~70% of the overall sequences included in analysis correspond to 5' end of the transcript [17], which included 5' UTRs. Hence, representation of di nucleotide repeats in this region would not affect the reading frame and thus tolerated more as compared to amino acid coding regions. However, certain frequency of di nucleotide could be abundant in the coding regions such (TC)n.(GA)n in the present case, which might represent GAG, AGA, UCU and CUC codon in a mRNA population and translate into the amino acids Arg, Glu, Ala and Leu, respectively. Ala and Leu are present in proteins at high frequencies of 8% and 10%, respectively [34]. (TC)n.(GA)n motifs were also the most frequently observed SSRs in different plant species including coffee, cereals and forage crops [23,26,31,34] and also in other perennial crops, such as eucalyptus [32], apple [35], strawberry [36] and citrus [37,38].

The most abundant tri nucleotide repeats observed in present study were (CAT)n.(ATG)n and (TTC)n.(GAA)n making up 18.9% each of total tri-repeats mined, which is the second most abundant motif in *Arabidopsis *[7]. Further, (CCG)n.(CGG)n repeats, which accounted for half of the tri repeats in rice, were rare in dicots (*Arabidopsis *and soybean) and moderately abundant in monocots other than rice [39], were found to be ~8% of mined tri-nucleotide repeats in present case. Parida et al [7], while analyzing the unigenes sequence data of five cereals and *Arabidopsis *observed that monocot and dicots possess common tri repeats. AGC/AGT/TCA/TCC/TCG/TCT (16.6%) coding for serine was the most abundant motifs in *Arabidopsis*, followed by glutamic acid (GAA/GAG, 12.3%) and leucine (CTA/CTC/CTG/CTT/CTC/TTA/TTG, 10.9%). Abundance of small/hydrophilic amino acid repeat motifs like that of alanine and serine in the unigenes of cereals and *Arabidopsis *was perhaps because these are tolerated in many proteins, while strong selection pressure possibly eliminates codon repeats encoding hydrophobic/other amino acids [40]. This observation suggested that considerable sequence divergence, since their early separation about 200 million year ago, between monocot and dicot has led to differential amino acid repeat motifs in the proteins, and that the selection has played a significant role in greater retention of those which are tolerated more.

The overall frequency of NR UGMS primer designation was 7.4% of the unigene sequence data. This figure is significantly higher than that found in the case of grapes and sugarcane [24,25], where the frequency of non-redundant SSRs in the total population of the clones in the cDNA library was 2.5% and 2.88%, respectively.

### Functional characterization

We characterized a set of unigenes containing successful UGMS markers by function. Since, the ESTs utilized here were obtained mostly from leaf and tender shoot tissues under natural environmental conditions hence, functional classification in relation to the organ or physiological conditions is not possible with the available data. However, a considerable frequency (60%) of unigenes containing UGMS markers was identified that correspond to the *Arabidopsis *gene sequence data base. These markers were present either in 5' UTR (52.8%) or in the ORFs (47.2%). As observed in earlier studies, majority of the transcripts detected through GO annotations represent enzymes of general metabolism [32,35,36]. However, transcripts related to biological process such as response to abiotic and biotic stresses can be readily mapped using the existing populations. This might reveal functional identity of particular marker locus. Since, these markers have recorded allelic variation across selected tea accessions, thereby working with these UGMS markers may arguably provide a shortcut to candidate genes and gene based functional markers. One of the approaches for their functional validation could be the establishment of association between trait phenotypes and UGMS markers based on these unigenes. In this context, UGMS primer pairs designed in tea would be very important assets for understanding functional diversity and also in marker-assisted breeding in this important commercial crop.

### Marker evaluation and polymorphism detection

Only 63.5% of the designed UGMS primer pairs proved to be functional. Similar findings were made for sugarcane [25], where 40% of all primer pairs failed to amplify the products. Possible explanation for this could be that primers extend across a splice site, the presence of large intron in the genomic sequence, or primers that were derived from chimeric cDNA clones. In general, because of conserved nature, limited polymorphism has been detected for EST-SSRs than the SSRs derived from genomic libraries [30,41,42]. Contrarily, a high level of polymorphism was detected in present case irrespective of the *Camellia *species. This is in agreement with some earlier studies that reported high [43,44] to even higher level of polymorphism with EST-SSR markers than genomic SSRs markers [6,45]. Furthermore, the ability to detect per primer a higher number of alleles than Zhao et al. [15] might be due to high abundance of di-repeats containing UGMS primer pairs (62%). However, the average number of alleles observed in this study remained comparatively lower than that for genomic microstellites (8.3 alleles and 7.8 alleles per primer, respectively) reported by Freeman et al. [13] and Hung et al. [14]. Detection of larger amplicons than the expected in few cases was probably due to the presence of introns which were excluded during processing of hnRNA into mRNA. Alternatively, multi-locus amplification detected with limited cases, were probably due to duplication and heterozygosity in tea, as was previously reported in tall fescue [44] and wheat [46]. The mean PIC estimated for genomic SSRs in tea [13], is higher than the estimated mean PIC for UGMS markers in the present study. The mean heterozygosities expected (*H*_*E*_; 0.654) and observed (*H*_*o*_; 0.413) estimates were also slightly less in the present study [15]. Further, test for IAM (Infinite allele model) and SMM models (Stepwise mutation model) for the UGMS loci showed excess heterozygosity in sign test and found to be significant in standardized and Wilcoxon test suggested that the studied marker loci did not show any bottleneck operating in the tea population and remain highly out breeding.

### Cross species amplification and sequence comparison of UGMS markers

UGMS markers identified in present study are highly transferable with in species and, frequently among species as reported in barley [26]. For instance, all the 61 UGMS markers developed for *C. sinensis *are fully transferable to *C. assamica *&*C. assamica *ssp. *lasiocalyx*, and at the various levels to *C. lutescens*; *C. irrawadiensis, C. japonica *white flower and *C. japonica *red flower. Similar pattern of cross transferability has been recorded in case of genomic SSRs in earlier studies in tea [13,14]. Interestingly, there were 15 (~25%) of the UGMS primer pairs which recorded cross-transferability in all the tested species. This suggested possible representation of highly conserved genes with some important biological/cellular/molecular functions. Further, conservation of repeat motif sequences at the species level and even at the multiple amplicons from the diploid genotypes suggests the wider utility of UGMS markers. Conservation of multiple repeats in diploid genotypes suggests presence of paralogs due to duplication of a particular locus within the genome.

### UGMS markers for evaluation of inter and intra specific genetic variations

The results obtained with 34 accessions tested from six tea species indicate that UGMS markers could be utilized for evaluation of genetic relationships within and at the species level. The genetic similarity matrix obtained from the two methods (Jaccard's and Nei & Li's) was significantly correlated confirm the utility of UGMS markers in tea. The genetic relationship among the cultivated *C. sinensis, C. assamica *and *C. assamica *ssp *lasicalayx *accessions reported in this study (GS; 28%) is comparable with RAPD based genetic relationship in 34 Keneyan accessions by Wachira et al. [47]. However, overall an extensive genetic variation was obtained at the intra and inter species level among the 34 accessions [48-52]. The difference in GS might be due to the use of different markers which most likely assay variation in the different genomic regions. However, SSR variation within the genic regions should be very critical for gene activity. Few of the UGMS markers that have shown significant hits in the Arabidopsis proteome can occupy certain positions in coding regions. Expansion and contraction of SSR repeats with known function in these regions might help to establish the association with phenotypic variation as reported earlier in the case of rice [53] and should detect "true genetic diversity" in crop species [26,54,55].

Cluster analysis of 34 tea accessions representing *C. sinensis *and related species revealed genetic affinities (Figure 5a &5b), which were broadly in agreement with known taxonomic classification of tea [56]. Traditionally, Cambod is considered a sub group of Assam type or sometimes referred to as a subspecies of Assamica known as *lasiocalyx *[56], therefore, majority of *C. assamica *(Assam) and *C. assamica *ssp. *lasiocalyx *(Cambod) tea accessions were clustered together in group II with high bootstrap values. Betjan 3/1, a fast growing, high quality tea accession, being an extreme Assam type was also clustered in this group [57]. Tea accessions namely TV-15 and TV-16 are moderately tolerant to tolerant to drought and hence clustered as a distinct subgroup under the major group II. Possible explanation of clustering TV-19 (Cambod; drought tolerant high yielder) and 124/53/8 (an extreme Cambod type) as an intermediate group between ornamental and cultivated accessions is due to their development from progenies of open pollinated seeds. TV-19 developed, introduced by T.C. Tunstall in the year 1918 was selected from progenies 124/53/25 and 124/41/42 of St.124 developed through open pollinated seeds collected from plants of 19/22 [58]. Further, *C. irrawadiensis *clustered along with two accessions of *C. japonica*, with red and white flowers in group III suggesting a possibility of introgressive hybridization between these two species. In general, limited introgressive hybridization had occurred in wild/ornamental species because of small populations and narrow geographical distributions. This might also be the reason for clustering of *C. lutescens *as a single solitary out-group in the present study. Conversely, self incompatibility and long term allogamy make the cultivated tea accessions highly heterogeneous and consequently with broad genetic variations [51].

## Conclusion

Our study revealed the insight of abundance and distribution of microsatellite in the expressed component of the tea genome. Sixty one UGMS markers developed and experimentally validated for genetic diversity analysis in different *Camellia *spp. will be enriching the limited existing microsatellite markers resource in tea. Most of the UGMS primers were highly polymorphic and were able to unambiguously differentiate the tea germplasm at the inter and intra specific levels. The use of these markers would reduce the cost and facilitate genetic diversity assessment, gene mapping and marker-aided selection in tea. Functional categorization of these UGMS markers corresponded to many genes with biological, cellular and molecular functions, and hence offer an opportunity to investigate the consequences of SSR polymorphism on gene functions.

## Methods

### Plant materials

Screening of newly identified UGMS markers was performed on a test array of 34 accessions of *Camellia *species (Table 6). This included 30 accessions of the main class of cultivated tea belonging to three major traditional varietal types namely *C. sinensis *(China type), C. *assamica *(Assam type) and *C. assamica *ssp.*lasiocalyx *(Cambod or Indian type). Three *Camellia *species comprising of *C. lutescens, C. irrawadiensis, C. japonica *(red flower), *C. japonica *(white flower), significantly exploited either in tea improvement programme as wilds and/or as ornamentals used for the examination of cross-species amplification of newly identified UGMS markers. The genomic DNA from the individual tea bush in each case was isolated from young leaves using CTAB method as described by Doyle and Doyle [59] with minor modifications.

**Table 6:** Tea accessions used for UGMS markers based genotyping analysis

S. No.	Accession Name	Species	Chromosome (2n)	Varietals type	Source
1.	Kangra Asha	*C. sinensis *(L) O. Kuntze	30	China	HPKV, Palampur
2.	Kangra Jat	*C. sinensis *(L) O. Kuntze	30	China	Kangra region
3.	UPASI 10	*C. sinensis *(L) O. Kuntze	30	China	Brookland Estate, The Nilgiris
4.	CSIN-303536	*C. sinensis *(L) O. Kuntze	30	China	NIVOT, Japan
5.	SA-6	*C. sinensis *(L) O. Kuntze	30	China	South India
6.	AV-2	*C. sinensis *(L) O. Kuntze	30	China	Makaibari TE, Darjeeling
7.	BS-54	*C. sinensis *(L) O. Kuntze	30	China	Banuri TEF, IHBT Palampur
8.	128/26/2 (Vimtal)	*C. sinensis *(L) O. Kuntze	30	China	Kumoun hill
9.	Teesta Valley-1	*C. sinensis *(L) O. Kuntze	30	China	Darjeeling
10.	Sikkim-1	*C. sinensis *(L) O. Kuntze	30	China	Darjeeling
11.	TV-15	*C. assamica*	30	Assam	NBA, Tocklai, Assam
12.	TV-16	*C. assamica*	30	Assam	NBA, Tocklai, Assam
13.	UPASI 18	*C. assamica*	30	Assam	Brookland Estate, The Nilgiris
14.	UPASI 13	*C. assamica*	30	Assam	Brookland Estate, The Nilgiris
15.	UPASI 6	*C. assamica*	30	Assam	Brookland Estate, The Nilgiris
16.	UPASI 9	*C. assamica*	30	Assam	Brookland Estate, The Nilgiris
17.	Teenali	*C. assamica*	30	Assam	Teenali, Assam
18.	4.6	*C. assamica*	-	Assam	Tocklai, Assam
19.	75.11	*C. assamica*	-	Assam	Upper Assam
20.	Betjan 3/1	*C. assamica*	30	Assam	Middle Assam
21.	TV-23	*C. assamica *sub. lasiocalyx	30	Cambod	NBA, Tocklai, Assam
22.	TV-19	*C. assamica *sub. lasiocalyx	30	Cambod	NBA, Tocklai, Assam
23.	TV-25	*C. assamica *sub. lasiocalyx	30	Cambod	NBA, Tocklai, Assam
24.	TV-20	*C. assamica *sub. lasiocalyx	30	Cambod	NBA, Tocklai, Assam
25.	TV-22	*C. assamica *sub. lasiocalyx	30	Cambod	NBA, Tocklai, Assam
26.	TV-26	*C. assamica *sub. lasiocalyx	30	Cambod	NBA, Tocklai, Assam
27.	C-6017	*C. assamica *sub. lasiocalyx	30	Cambod	South India
28.	124/48/8	*C. assamica *sub. lasiocalyx	30	Cambod	Tocklai, Assam
29.	521-Aya.DA4	*C. assamica *sub. lasiocalyx	30	Cambod	Tocklai, Assam
30.	523/SP-I	*C. assamica *sub. lasiocalyx	30	Cambod	Tocklai, Assam
31.	*C. lutescens*	*C. lutescens*	-	Related species	South India
32.	*C. irrawadiensis*	*C. irrawadiensis*	-	Related species	South India
33.	*C. japonica* (RF; Red flower)	*C. japonica*	-	Related species	South India
34.	*C. japonica* (WF; White flower)	*C. japonica*	-	Related species	South India

### EST data mining, unigenes prediction and SSR detection

A total of 2,181 FASTA formatted EST sequences in *Camellia sinensis *were retrieved on May 21, 2006 from the National Center for Biotechnology Information (NCBI; ) for subsequent data mining. This dataset was scanned and assembled using SeqMan DNA Star lasergene version 7.1 (DNASTAR Inc, Madison, WI) and predicted potential unigenes that contained contigs and singletons from all the EST sequences with parameters (match size: 5, minimum match percentage: 80, match spacing: 150, gap penalty: 0.00, gap length penalty: 0.70, maximum mismatch bases: 15). Further, gaps in the aligned sequences due to limited dataset were removed on the basis of probability function of nucleotide occurring at the particular position using Gene Runner version 3.05 nucleotide windows and stored as the relational database. All the unigenes were subsequently searched individually for the presence of SSRs with help of Repeat masker  and SSRs with a minimum length of ≥ 18 bp (di & tri) and ≥ 15 bp (tetra, penta & hexa) were masked. These parameters were chosen to identify SSRs with high polymorphic rate. Uninterrupted type of microsatellites in the present case are continuous, however interrupted one's are defined as presence of ≤8 arbitrary nucleotides in between ≥2 SSR motifs.

### Functional characterization

Initially an annotation of the SSR containing unigenes was done using BLAST in the complete GenBank NR database, and the complete coding sequences from *Arabidopsis *[60]. Further classification of these unigenes was done using Gene Ontology (GO) system [19]. All the *Arabidopsis *hits with an high expectation values (Table 2) were submitted to the GO annotation search tool at TAIR website [20,61], and relative gene counts assigned to the different GO functional classes were displayed as pie chart using Microsoft Excel.

Primer pairs from the SSR containing unigenes were designed with Gene Runner 3.05 software with the following criteria; i) nucleotide length of 18 – 22 base pairs, ii) a T_m _value of 50°C to 60°C, iii) the 3' end base with a G or C, preferably and iv) an amplified fragment size of 100 – 350 bp. The formation of secondary structure and primer dimmers were critically monitored to get success of the primers. The names of the primers were prefixed as TUGMS (Tea unigene derived microsatellite) markers as the source is from *Camellia sinensis *unigene database (Additional file 1).

### PCR amplification

PCR amplification of all the primers were performed in 10 μl reaction volume consisting 1× PCR buffer (10 mM Tris- pH 9.0, 50 mM KCl, 0.01% Geletin, 1.5 mM MgCl_2_), 200 μM of each dNTPs, 15 ng each of forward and reverse primers, 0.2 U Taq DNA polymerase (Bangalore Genei) and 20 ng of template DNA. Forward primer was labeled with γ^33^P ATP (phosphorylation by T_4 _polynucleotide kinase). The PCR protocol was consisted of one denaturation cycle at 94°C for 4 min, followed by 35 cycles of 94°C for 1 min, annealing at optimum temperature (*T*_*a*_) (Table 3) for 1 min, and extension at 72°C for 2 min. The final extension cycle was carried out at 72°C for 7 min. All the PCR reactions were carried in I-Cycler (Bio-Rad).

PCR fragments were separated on denaturing polyacrylamide gels consisting of 7% polyacrylamide (AA: BIS = 19:1) and 7 M urea in 1× TBE buffer. The PCR reactions were mixed with equal volume of loading buffer (98% formamide containing 0.8 mM EDTA and 0.025% of each bromophenol blue and xylene cyanol), denatured at 94°C for 5 min and snap cooled on ice. Samples were loaded in preheated Sequi-Gen GT sequencing cells (Bio Rad, Australia), which run at 60 W for 1.5 up to 2.0 hrs depending upon the fragment sizes to be separated. After run, the gel was blotted on the chromatographic paper CP3M (PALL Life Sciences) and vacuum dried for two hrs before subjecting it to autoradiography for 2–3 days at -70°C depending on the signal intensity. The size of the fragments was estimated using 20 bp DNA size standard (Cambrex Bioproduct, USA).

### Sequencing of PCR product

PCR products were separated on polyacralamide gel. Selected fragments were excised and dipped in 10 μl nuclease free water for 30 min. Another round of PCR was made following the same protocol with extracted DNA as template. The PCR products were separated on 2% Seakem LE agarose (Cambrex bioproduct, USA) gel and extracted using kit (Montage Millipore Corp, USA). DNA concentration in each case was measured using NanoDrop 1000 (NanoDrop spectrophotometer, USA). The PCR products were ligated to pGEM-T easy vector (Promega, USA). Sequencing was performed using ABI 3730 xl DNA Analyzer in 20 μl of sequencing reactions consisted of 250 ng of template DNA, 4.0 pmol universal sequencing primer, 8 μl of ready reaction mix BigDye terminator (Applied Biosystem Version 3.1). The base calling and post processing of the sequence data were done using sequence analysis software (Applied Biosystem Version 5.2). The nucleotide sequences were aligned using DNASTAR software (MegAlign DNA Star lasergene version 7.1) using Clustal W algorithm method.

### Data analysis

The fragment size is reported for the most intensely amplified band for each UGMS locus or average stutter if the intensity was same using 20 bp DNA size standard. Null alleles were assigned to genotypes with confirmed no amplification products under the standard conditions. The polymorphism determined according to the presence (1) or absence (0) and data was entered in a binary data matrix as discrete variables. Jaccard's coefficient was calculated to develop a phylogenetic tree on the unweighted pair group method with arithmetic mean (UPGMA). The computer package NTSYS-pc Ver. 2.02e, Rohlf, [62] was used for cluster analysis and matrix correlation. Genetic similarities (GS) based on Jaccards's coefficient were again checked by Nei and Li's formula [63] as GS_xy _= 2N_xy _(N_x _+ N_y_), where N_xy _is number of bands shared in accessions X and Y, N_x _is the number of bands shared in accession X, N_y _is the number of fragments shared in accessions Y, were calculated using TREECON software package [64]. The robustness of neighbour joining tree was evaluated by bootstrapping (1000 bootstrap replicate) using TREECON. Popgene software package by Yeh et al. [65] was used to calculate heterozygosity (observed & expected). The polymorphism information content (PIC) of each marker was calculated according to Anderson et al. [66]:



Where P_ij _is the frequency of the j^th ^pattern for marker i and summation extends over n patterns.

The fit of each locus distribution to expected distribution under two different mutation models, the IAM (infinite allele model) and SMM (step mutation model) was tested using the program BOTTLENECK [67]. Considering the locus limitations in data analysis using BOTTLENECK, particularly 40 UGMS loci having detected PIC ≥ 3.0 were selected. Observed allele frequency and sample sizes were input parameters. These analyses provide a test statistic, the Wilcoxon sign-rank test, for the probability that an observed allele distribution with a given heterozygosity (gene diversity) was generated under each of the two mutation models.

## Authors' contributions

RKS conceived the study, participated in designing, coordination, data analysis, interpretation, checked the data, drafted, reviewed and improved the manuscript. PB carried out mining of EST data, unigenes prediction, GO study, analysis of repeat type and frequency of microsatellites, genotyping, and sequencing and helped in drafting the manuscript. RN carried out the microsatellite analysis for genotyping. TM helped in interpretations and improved the manuscript. PSA helped in overall coordination. All authors have read and approved the final manuscript.

## Supplementary Material

Additional file 1 **Details of SSRs containing tea unigenes**. Unigene designation, nucleotide sequences and accessions numbers of contributing ESTs are given. Click here for file
