# Supplementary material for: Identification, characterization and utilization of unigene derived microsatellite markers in tea (Camellia sinensis L.)
Source: BMC Plant Biol. 2009 May 11;9:53. doi: 10.1186/1471-2229-9-53 (PMC2693106; doi:10.1186/1471-2229-9-53)
Supplement: Additional file 1 — Details of SSRs containing tea unigenes. Unigene designation, nucleotide sequences and accessions numbers of contributing ESTs are given. [file 1471-2229-9-53-S1.doc]

**Additional file 1- Details of SSRs containing tea unigenes**

| **S.No.** | **Unigene detail** | **Nucleotide sequence *** | **Contributing ESTs**  **(Accessions number)** | **Successfully utilized for primer pairs designing** | **Amplification validation** |
| --- | --- | --- | --- | --- | --- |
| 1 | TUG1 | TAGAGCGTGTAAAAAGAAACATAGAAGAGAGACGTGCTTCTTTGTATATATAAATTTGTATGTATGTTTATTTATGGTAAAGAAAAAAGACACAAAACTTCTGTTTTGTTTAGAGGATCAGCTGCTTCAAGTTGAGTTTGTCCGAATTTGTTGGCTGTTAACTTACATGTATATATATATATATATATAACAGATTGAGAAGTTAACTCTAAATACAAGTGAAAACCATCCCTTGCAAAAAAAAAAA | DN976160, DN976164 | YES | YES |
| 2 | TUG2 | TATTATAGAGTGAGAGAGAAATATATATATATATATATATATATGTTTGGTGGAGGCGTTGTATCATTATTATCATCCAATTAAACTATTTAGGATTTTGATTTGTATAGTCTCTCTCGAATTAATGAACAGTTTTCGCTATTAGTAGAGGATTAGTGAAACTTTTATTAATTTTTATTTATTGCTAAAAAAAAAAA | DN976107 | NO | NO |
| 3 | TUG3 | TTCGAGCGGCCGCCCGGGCAGGTACTAAGCAAGCTCTCATAAGTAGCATTCCAACTCAATCTAGGTTCACGGACTTGTCCATGGTTCTTAGACCTTTTATTTCCAATCCACTGAAGCCTGGTCTGATACCAGAGAAGAAGGCCATGATTTACGAATTCAGAATGGTTGCTAGAGATGCCAGTGCCACTACTCAGGCTCTCGTTTGACACGCTGATAGATGAGATGCTTCTCTGAGAATGAACAGCGTTATTGTCCATCTCATATGTGCTGGTGCTCCAAAAATCTTCATATGCACTGCTTTTCCTCACCCTCTGCCCAATTCTGAGCTCCTTTGCTGGCGCATCCACAGCAATAACTGGTGTGGGTTTGGAATAGCATCCCATACAACCTCCCATGCAAGCGACGAAGCGATCGATCGAATCGAATCAGTGAATAAAGCGTGTATAATCATCAGCCTTCTCGTCCTCCTTAACTAACAAAGGCCGTAAAATCGACACATCGAATCTCCAATACCTTCAACAAAGAGAGGCGTATGGAAAAGCTGAGAAAATGGATGAGAATCAAATGATTTGAGATCTGTCTAGCGAAAACCAAGTTCCCAANGTTCAGCGCTCTCTCGCTCTCTCTCTCTCTCTCTGAGCTTTCTCTCTCTTCCGCCGCCACTTCACCTCAGTGGTTTGCTTCCAAAAACTNCAGTGGATTGGAGCGAANAAACACCTTAGTAAGCTTGAGAGTACCTCGGCCGCGANCACGCTA | CV699866 | YES | YES |
| 4 | TUG4 | TTTTTTTTTTTTTTTTTTTTTTTTTTTTTTRYAAGTAGAATAGATAGATATATAGATTAGATAAGATGATTTTATCACGTACAATCACCACCGACTCGATGACATAATACAACTAGAAAGACTTGACAGTAGTACCAAATACAATAGTAACARCCACCACCAACATAGTACATACACTCTCTCTCTCTCTCTCTCTCTCACACACACACACACACACACACAAATGGGGACTCTGGGTGGAATCCACACTTTCATCTTATTATTATTCAAAACAGCRGTAAATCTGTGCTAGCTGAAAATCAATGGTCCATCAATCTCAATGCACCTTGGAGCAGTCGGTGGAGGGGCTGATCTTGTAAGGAACGTTAACACCGCATTTGCCAGGGAGGGAAGCAGCCRMATGCGGGATTAATACCAGTAATGCTTTTAGAAGCCTGTTGCAAGCATATGCAAGTTTGCTTGCGGTCGGCAGGGGTCTTAGCAGCCGAGTTAATGGACCTAACCCCATTGCAGCACGCTGCTGGCAACGCCCCTCCCTTCCTCAGGTACCCAATGCACTGTGCCAGGCTCCCCTGCACCTGACCGCACGTAACCGCTTCCGAATACGGTGCAACCACCACCACCATGCACAACACCGCCAAGCATGCAATCTTCACCCCTGCCATTTCTTACTAATTCACCACCACCACTTAAGTACAACTCTTTTCTTTTTCTCTCTTTCTTTTGGTTATTGTTGCTATATTTGGGTCCCCG | CV013834,CV066940,CV067012,  CV014386,CV013970,CV699845  CV699741,CV014015,CV014529,  CV014132,CV014931,CV014029,  CV014848,CV014572,CV014399. | YES | YES |
| 5 | TUG5 | TAGCGTGGTCGGGCCGAGGTACNTACTTACAGCTTCTTGGAGGAGAACAAGCTGATGATTGGGTGAGAATTTAGGGTGGAGTTTTATGCAAGGGCAGGGCCAGTGCTGCAATTGCAGCAACTGATAATTTGATATTTGATATTTTCCTCCTAGCGTGGTCGCGGCCGAGGTCTCGGCCCTGTCTCTTGTGCAAGAGAGCTTCAAGCCGATCGATCCCAACTCACTCTCTCTCTCTCTCTCTCTTCAATAGTCTTTTCGTAGTCTAAAGGTTTTGATAAATGGGTAAGGAAAAAGTTCACATTAACATTGTGGTCATTGGCCATGTCGACTCTGGGAAGTCGACCACCACTGGTCATTTGATCTACAAGCTTGGGGGTATTGACAAGCGTGTCATCGAGAGATTTGAGAAGGAAGCTGCTGAGATGAACAAACGTTCATTCAAGTATGCATGGGTCTTGGACAAGCTCAAGGCTGAACGCGAACGTGGTATTACCATTGATATTGCTTTGTGGAAGTTTGAGACCACTAAATGCTACTGCACTGTCATTGATGCCCCTGGCCATCGTGACTTTATCAAGAACATGATCACTGGTACCTGCCCCGGGCGGCCGCTCGAA | CV699652,CV699749,CV699656,  CV699604 | YES | NO |
| 6 | TUG6 | TAGCGTGGTCGCGGCCGAGGTACTACAGATGGAGGCCAAGGCATCCTCCACACATGGACACTCGTCTCTCTTGTGCTGATAACGCACATCCTGATGAACTGGATGAAGAATTTGATACTTTTCCAACTTCCCGTCCTAATGATATTGTGAGGATGAGGTATGATCGTTTGAGAAGTATTGCAGGCAGGATTCAGACAGTGGTTGGTGATTTGGCAACTCAAGGGGAGAGGCTGCAGTCCTTGCTGAGCTGGAGAGATCCAAGAGCTACTGCCCTGTTTGTGATTTTCTGCTTGGTTGCTGCAATAGTTCTCTATGTTACACCATTCCAAGTTGTGGCGCTTATCACTGGATTTTACGTGTTAAGGCACCCAAGGTTCCGTCATAAGCTGCCTTCAGTGCCCCTCAATTTCTTCAGGAGGCTGCCTGCAAGAACTGACTGCATGTTATGAGCAAAGCCACCGCCAGTTTTTTAAGCCTTCTATTCTATTCTCCTTCAATGTTTATTAAGCACCGCCAGTTCAAAACCAGTTGGGGAAGAAGATGTGGAGACTTATGGAGCTTGCTTGCTTTCTTTCACCTAGCAGTGTGGTGAATATTGGGTCCCCGGTATGATGNGTNATCTCTCTCTCTCTCTCTCTCTCTCTCTCTCCCGGGCTTAGTAGATTTTATGGATTTGCAGNTTATTTATATGCCNTCATANGGTTCCTTTATTCAAAGATTGACCTCTGGTACCTGCCCCCGGGCCGGCCGTTTCNNAAA | CV699614 | YES | NO |
| 7 | TUG7 | GGTATGTATATCCCCGCCAATTTTGGGCTGGCCTTTCCCCTTCCCCTGTGGGCCAAATTCCGGGGTGTTAAACCGGTTTCCCCTTTACCAAAGAGTCCTAGGCCCCCCTTCCCCAACCCGGACTTGCTCCAAGGTGCAATTTGAGATTTGATTGGACCACTTGATTTTCAGCTAGCACCAGATTTACCGCCTGTTTTGAATAATAATAAGATGAAAGTGKGGATAGTACMAMCMCASARTCYYCATTTGWGWGWGWGWGWGWKWRAGAGAGAGAGAGAGAGAGAGAGTGTATGTAACTATGTTGGTGGTGGYTGTTACCTACTKGTMTTKGGTACTACTGTCAAGTCTTTCTAGTTGTATTATGTCATCGAGTCGGTGGTGATTGTACGTGATAAAATCATCTTATCTAATCTATATATCTATCTATTCTACTYAAAAAAAAAAAAAAAAAAAAAAAAACAAAAAAACA | CV067127, CV014439 | YES | YES |
| 8 | TUG8 | AAGGGAGAGTTCTTCGCATCCTCCTCGGTGAGACCGAGAGAGAGCTTGGTGATGACAGTACCGGTTACGAAGAAACCGACCAAGAACGGCCAGTTCCTACTCCATTCTCGCCGGAAGAAAACGGGTCACACRRATCGSATCCYCAAACATACCTCTCTCTCTCTCTCTCTCTCTCTCTCACATGGCTCCTCTAACCTGHTCTCACCACCCACCTTCCAAGGCCTCAGAMCCACCACCAAATCCCCACTCTCCCTCACCAAATCCATATCCACCGTCAAATTCACCCCCAAAAAACGATGCAGCGGCGCCATTAAAGCCGAATTGAACACATCTCTGGTCATCAGTCTCAGCACTGGTCTGTCCCTGTTTTTGGGCAGGTTCGTGTTCTTCAATTTCCAGAGAGAGAATGTGGCCAAACAGGTTCCCGAACAGAACGGTATGACCCATTTCGAAGCTGGTGATTCTCGAGCCAAAGAGTATGTGAGTCTCCTCAAATCCAACGAGCCAGTTRGGGTTCAACATCGTCGATGTCTTGGCTTGGGGTTCCATTGGACACATCGTTGCTTACTATATACTTGCTACCTCTAGCAATGGATATGATCCCAAGTTCTTTGGATGAATTTGAATTTGAATTTGAATTTGGATTGTTGTTGGGTTGTGCTAGTAATTCATGTGTATAGGTGGGGAGGCTACAATTTWTCTMTGTTGTTCTTCATGTTTTCGAATTAATTTGAAGCTTTGCTGTGTGTGTGGTSSWAGGAGGGGATGTGTGTAGTCTGGATGGTTATTTAGATCTTTTTCAAAAAAAAAAAAAATTG | CV067252,CV013915,CVO13656,  CV067044 | YES | NO |
| 9 | TUG9 | GGGGAGCTACAGAGCTGCCAACAGTTTTATAATCCATTTAAATCAAATTAAMGCVGGGGACTGTAAAAATATCAGCAGAGAGAGAGAGAGATCTATCATTCATTTTTTTTTCAAATTTGATCAGKGTTTGAAGTTGAGGAGATGGCAACAGCAGTGATGACCTCTCTCCCACAGTTCAGTGGGCTAAGACCCAAATCATCATCATCTGCTTCACCAATTCAAACCCCTGGTAGCAGTTCAACCAATGAGACGTAGGGGGAAGGGAGCTTTAGGCGCTCGCTGCGATTTCATCGGCTCCCCCACAAATTTGATAATGGTGTTTTCAACAAGCTTGATGCTGTTTGCGGGTCGGTTGGGGTTGGCTCCATCAGCAAACAGGAAAGCCACAGCAGGGTTGAAGCTTGAGATAAGGGAGTCTGGCCTTCAAACTGGTGACCCTGCTGGTTTCACCCTTGCAGATACCTTGGCTTGTGGCTCTTTKGGTCACATTATKGGTGTWGGGGTTGTTCTTGGCCTCAAGAACATKGGTGCTCTTTGAATAAACATATGTTCTAAAATTKGTTAATTAATATATAATAGAACTTTCTKGTTGTTTCAATWWAMAACAAAAWAAAAAAAAAACCC | CV014010,CV067072,CV013955,  CV014805, CV014551,CVO14715  CV014598 | N0 | N0 |
| 10 | TUG10 | ACGCCCGGGGAGCATTGAAGCGAGAAATTTCTGTCAACAATACTTCACTCTCTCTCTCTCTCTCTCTCTCTCTCTCTAGAAATGGCATCAAGCCATTCCACTGGCCTGGGCGCTCCGAAGTTCTCTCACTGTTCCGCTCTCTMMTCMGWWCMTCCSGCTAAATTTTGCCGAGCCTTACCGAACATTCATGCGGAGGTTACTCGAAGCGTTCGCTACCAATCGACGCCGTTTCCGCCAAGATACAGAAGGCCTCGTCTGGACCCGTTCATCGGATCTGCACTTCCGCTTTCTTCCGATGGGAAGTCTCAGCTCGAGGTCGCCACTAGACAGGCCATCGTGTACTCTACTATATGCTCCCAAGGTCAAGAGCGTCACTGGAGATTAAGAATTGAAGAGAAGCCGCGATTTTGGATGGTATTTTTTAGCTCTTGATTCATGTCAATTTTCAAACTCTTTTTGTTTTGCTTGGTCTTCGTTTTTGCCACCTGGGTTTGGGTGCCCAGTGTTATAGAACTTGAGAATTTGTCGTGAATGTGACGAAATTTCTGAACTTGTAGCGTAAGAATTAAGTTGTTTTTTTGAAATAGCAGAGGAAATATGGTATGAGTTGAATGTAAAAAAAAAAAAAAAAAAA | CV067063,CV067066 | YES | NO |
| 11 | TUG11 | GGGGAGTGTTTGTTTGAATAGGAACTGGAGAGAGAGCGAGAGAGAGAGAGAGCCGAGAGAGAGAGAGAGAGATGGCGTCGAACGCGGCCGTACCGTTCTGGAGAGCGGCGGGGATGACATACATAACGTACTCGAACATATGCGCGAACATGGTGAGGAACTGCCTCAAAGAACCCTACAAATCTGAAGCCCTAGCTCGCGAGAAGGTCCATTTTTCCGTCGCCAAATGGGTCGATGGCAAACCTCAAAAACCCATTGTTCGTTCGGATGATCCTGAAGAATGAAGCAGGGAGTCAGTTGCCAAATCTCTTGTTGGGAAAGTTCTTTTTTTTGCTGAAGTTCTTCCATTTCATCGAGTTTAGTGGGTATGATTTATATTTGGATTTGTAACTGCCTAAGCACTTGAAACAATGCTTGTAATACCGGGTTTTGCATGGAACTGAACTTGTAATAATGTGGCTCATGCTGCTGAAAACATGTTCACAGTTTTGTTGTTGAATGGGTTTCCCATATTGTCAAAAAAAAAAAAAAAAAAAAAAAACCCCCAAACAT | CV066980 | YES | YES |
| 12 | TUG12 | GAAGTTTGTTGAGAGTGCTGCAACTTCTTTCAGTGTTGCTTAATTTATATTTCTCTTTCTATATATATATATATATATACATTGATCAAATGTGGATTTGTATTTTGTAATTTGGTTGAGTTGTATGGAACCTTGTTCCCCCCAAATTTAGATCTGTTCAAGTGAATGTGGATATTTCTTTCTAAAAAAAAAAAAAAAAAAAAAAAACCCCCAAACAT | CV066969 | YES | YES |
| 13 | TUG13 | GAAAACAGAGGGAGGAAGAGAGAGAAGAAGAGAAAGAAGAGAGGAAGAGGGAACAGAGAGAGAGAGGCTCCACCCATACGGCGCCGCCATCCTCCGTGTGTGCATACACAGATCTGTATATCTGTTGATGATCTGTGTCTCTCTGTTCCCCGTTCTGTTGATGATCTGTGTGTGTGTGTGTACAGATCTGTCTGTGTCTGTGTGTATTGATGTTCTGTGAGTGTGTGAGTTATGAAAGAAGAAAAGGAATAGAAAAAGAGGAGGAAAAGATGATGTGTGGTGATGATGATGATGTGTTGTTTGATGGTGATGCTCTCCCTCGGTTGATGATCATGATCTGAGTTGGTTGGTGCTCTCTCTCTCTCTCTCTCTCTCTCTCTCTCTCTCTCTCTCTCTGTCTCCTTATCTTCACCTCTGCTTCAGATAAAAAAAGAGCCAAAAAAAAAAAATCCAAAACGTCGTTTTGAAAAAAAAAAAAAAAAAAAAAAAAAAAAAAACAT | CV066965 | YES | YES |
| 14 | TUG14 | ATGTTTGGTGGTTTTTTTTTTTTTTTTTTTTATACAACTTTTTTCTTTATGAAATCACTTCACTTATTTAATACAGTCCAACATCCCAGAGAGAGAGAGAGAGAGAGAGATGTGCCTAGGAAACAAAGAATCACTAGTGTTTTTAAGTTTGAGATTACTATTACATACATGAAGAAAAAACTACATGACAGTTTCTTGCAAAAATCTCAAAATCTAACATTTACTTTTCTTCTTCCTCATCTGTGATAAATTAGCGTGGTCGCGGCCGAGGTGCGAAGAAGAAGAAGGCAACATTGAGCAACACAAGGGGCTCAATATCACCAATTGGAACTCCTGTCTCTATGTTTAGCTGTGCCAGAGCTCCTTTCCCTGTTATCATTTCTCCAATTATAGAGAATACAATCCCCAACTGAGCCAATCGTCCCACGAACAGCTCGTTTGATTTTGTAAATCCAAATAGTGGACCTCCTTCTTTAAGGCCCAACGCGGATCTGAGTCCTTTGCCAGGAGGGATGACAGCCCTTTCAAGGCCAGTGGGAGGGTCATCGACAAAGCGGCCTCTATCTCCCAAAGCTCCAATGGCTCCAAGAAGGGTGAAAAGGATGAAGAAAAGAAGAAGAGGCTCAGCTTCATAGATGGGAATACCGGTCTCAAGATTGAGTTGTGCTAGAATTCCTTTCCCTGTTATTGCTTCYCCCAACAGAGATGCAGCAAAGCCGATCATCGCAACACGACCCACAAAGAGCTCATTTTGCTTTGTAAACCCAATCCCACCCGACGTGCCAAAAATACCACTTTCAACCTTCGGCTTAGGCTCGGCAACCTTCTTGACAGGGGCAGCTTTGGTTTTGGATTTGAAGAGAGCAATGGTGGTAGTCTGATGAAATGAAGAAGAAGATGGAAGTGGAGGTAGTAAGATGTGAGAGAATGGTTTGGGCCTTAGACTTTCAAGTAATGGTTGTCTCTTCAAATCCAATGCATGACTATAGACACTTGAACTAGAAGTGAGCAACATGGTCTGAGCCATAGATCTCTAGCTCTGKTTTGATATTTTTTTTTTGACCTGCCCGRGVVGRVYAAGGTMAGRAWTAYTTTTGTTGCTCCCTCACTCCCCC | CV066936, CV699709,CV699710  CV014116,,CV013622, CV014733,  CV014145 | N0 | N0 |
| 15 | TUG15 | TAGCGTGGTCGCGGCCGAGGTTTTTTTTTTTTTTTTTTTTTTTTTTCGGATACATAATAGCATAATTTTCATTGAAAATAACTTAATAAAAGCTTCAACAGTAGATAGGACACCAAATTAAAAACACAGAACCGACAAAGCACTAGAAACCAGACAACAACTAACCCTAACCACCTATACAAAACAACTAACCATTGATTCACAATCACATTAGGCAACAAATCTCAAACCCTAAGCACGTTCGCCTCTGATGCGGCGAGCAAGCTGCATATCCTTAGGCATAATCGTGACACGCTTAGCGTGAATAGCACAAAGATTGGTGTCTTCAAACAGTCCAACAAGGTAAGACTCAGCAGCCTCCTGGAGGGCCGCAACAGCGGAGCTCTGGAATCGGAGATCGGTCTTGAAGTCCTGAGCGATCTCACGAACAAGTCTCTGGAATGGAAGCTTTCGGATCAGAAGCTCTGTGGATTTCTGGTACTTTCGGATCTCCCGGAGCGCCACCGTGCCGGGGCGGAAGCGGTGGGGCTTCTTCACACCACCGGTGGCTGGAGCGGACTTGCGAGCGGCCCTTGGTGGCGAGTTGCTTCCTTGGTGCCTTGCCTCCGGTGGACTTCCGAGCTGTCTGCTTTGTACGAGCCATTTGAGACTAGGGTCTTAGAGAGAGAAAAAAAACTAGGCCTTGAGAGAGAGAGAGAGAGAGAGAGAGAGAGAATACTGRATGTGGTCCCCGCCGT | CV699537,CV013548,CV014230,  CV067117,CV066957,DN976200,  CV067162,CV067124,CV013651,  CV014574,CV013835,CV014937,  CV014506,CV014692. | YES | YES |
| 16 | TUG16 | GGGGATGTATTTTTACAAGGTGGGAGAGGGAGGGAGAGAGAGAGAGAGAGGGAGAATGGCGGGAGTTGAAGGAGCGAGAAAGTGAGGTTACCTTGGATGAGCTGAAGAAGAAAATGGCAGATTTTGCTAGGGAAAGAGACTGGGATCAGTTTCATAGCCCTAGAAATCTTCTTTGGGCTCTGGTGGGAGAAGTGGGAGAGTTGTCTGAGATATTTCAGTGGAAAGGGGAGGTGCCAAGAGGTCTGCCAGATTGGGAAGAGGAAGAGAGACAACACTTGGGTGAGGAGCTCTCAGACGTG | CV014911 | YES | NO |
| 17 | TUG17 | GGGGAATTTCAGACAGACACACGCACCCTTTCTCTCGTCTCTCTCTTCGCCCCCTCTCTCATCTCTCTCTCTCTCTCTACCCCCAAAACCCTACCCATCGTCGTCCTCGTCCAGCGGTCTACACTAAACAGTCGACCGACGATCTACTACACTGAACGGCGATGGGCTATCGATCTACACCGAACGATATTCTCCTACGATCTGCTCGTCTCTGCTCCTGTTCTACCCTGACGATGTCTCGAAGGTATGACAGCCGTACAACAATCTTCTCTCCAGAAGGTCGTTTGTACCAGGTTGAATATGCAATGGTAGCAATGGGAAATGCTGGGACTGCCATAGGGATACTATCAAAAGATGGGGTCGTACTGGTTGGAGAAAAGAAAGTCACTTCCAAACTCCTACAGACCTCAACATCCACCGAGAAAATGTACAAGATTGATGACCATGTTGCATGTGCCGGTGGCTGGAATCATGTCCGATGCCAACATCCTCATCAACACAGCTCGGGTCCAAGCTCAGCGCTACACGTATGCTTACCAAGAACCAATGCCAGTTGAACAGCTGGTTCAATCTCTATGTGACACCAAACAAGGATACACACAATTCGGTGGGCTTCGACCATT | CV014908 | YES | YES |
| 18 | TUG18 | GGGGAAGAAAAAAAAAGTTGAGAGAGAGAGAGAGAGAGAGATGCAGATTTTCGTGAAAACCCTAACGGGGAAGACGATAACTCTCGAGGTGGAGAGCAGCGACACCATCGACAACGTCAAGGCCAAGATTCAAGACAAGGAAGGAATTCCACCGGATCAGYAGCGTCTGATCTTCGCTGGAAAGCAGCTTGtAAGATGGCCGAACCCTCGCCGACTACAACATCCAGAAAGAGTCTACGCTTCATCTTGTRTTGAGGCTTAGAGGAGGGATTATAGAGCCTTCTTTGATGGCTTKGGCYCGCAAATACAATCAAGACAAGATGATCTGCAGGAAATGCTATGCTCGCCTGCATCCAAGGGCTGTGAACTGCCGGAAGAAGAAGTGTGGCCATAGCAACCAGCTTAGGCCCAAGAAGAAGTATCAAGTGAACACTAGTGAAATTTCTGCATCTAGAGGTGGATCCGGAGCATCAAATTCTAAACAAAGCAAGTACAACCTTTTATTTCAGCAGACATTATCTCCATATTATGTTTTGTGATTCTCCTTGAAGCATTTTACTTGTAATTGGTACCGGATTGGATCTTGAATTTACCATATATCATGATGTTCCTCAATTTTTGATGAA | CV067133 | YES | YES |
| 19 | TUG19 | GGKGGCARCTGATGCGAGAGAGAGAGAGAGAGAGAGAGATRGTGTARGTSMYCGATATAGAGAGAGAAAGCTGTAGAAGAAAGAGATGGAAGGAGACAGGAGAGTGGGTGTGGCTGTTGATTTCTCGGCATGTAGCAGAGAAGCACTGAAGTGGGCTGGTGGATAACATTGTCCGCGACGGGGATCATCTTATCCTCGTCACCGTTCTCCCCGAAGGACACTACGAGGAGGGCGAGATGCAGCTTTGGGGAACCACCGGCTCCCCTCTAATCCCTTTGAAACGAGTTTCTCGTGAGCCCACCATCATGAAGAAATACTGGTGTGAAGCCCGACCCCTGATAACWTKGGACATTGGCCAATACTGCTGCTAGGCAGAAAGAGATTATGGTGCTTATGAAGATCTACTGGGGARATGCTCGGGATAAGATATGTGAAGCAATKGATAATATTCCTCTTAGCTGCCCTTGTTATAGGGAACCGAGGGCTCGGCAAGCTCAARAGGGCTATCATGGGCAGTGTTAGCAAACTACGTGGTGAACAGTGCCTCTTGTCCTGTTACAGTCGTGAAGTCGTGAAGAATGCAGATCATGAATGACTGCAATGCAGTATCCTGCTGGATTTCTAGTATGAACTTGTATCAATTGGTCATACCGATTGTAATTTACCTTCGAGTTTGAATTTAAGTTGCCGTGTGTGTGTAATGTATGTTGTGTAATCTAAAATCTTTGCCTGTGGCAGTGTTATGGACTTATAGTTTGCTGTAATCCAGTGGTCAAGAAGGAATAAATTTTGTTTTTGAAAAAAAAAAAA | CV014348,CV014852,CV067004 | N0 | N0 |
| 20 | TUG20 | GGGGAATTTCATCACTCAAACGGTCCATCTCTCTCCTCTGTGCTTTTCACACTCTCTCTCTTTCTCTGCAACTCTCTCTCTCTCTCTCTCTCTCTCCCTACAAAGATGCGTGAGATTCTGCATATCCAAGGAGGCCAATGCGGGAACCAGATCGGAGCGAAGTTCTGGGAAGTGGTATGTGCAGAGCACGGGATCGACTACAGCGGCAAGTACAACGGTGACTCCGATCTACAGCTCGAGCGAGTCAATGTCTACTACAACGAAGCCAGCTGTGGCCGCTTCGTTCCCCGAGCCGTCCTCATGGATCTCGAACCGGGCACCATGGACAGCGTCCGCTCCGGTCCCTACGGCCAGATCTTTCGACCCGACAACTTCGTCTTCGGCCAGTCCGGCGCCGGCAACAATTGGGGCCAAAGGTCACTACACCGAGGGAGCCGAACTCATCGACTCGGTTCTCGATGTCGTTCGCAAAGAAGCAGAGAATTGTGACTGTCTCCAAGGGTTTCAGGTGTGTCATTCGTTGGGAGGAGGTACTGGATCGGGGATGGGGACACTATTGATATCGAAGATTAGGGAGGAGTATCCGGATCGGATGATGCTGACTTTCTCCGTGTTTCCGTCGCCAAAGGTG | CV014798 | YES | YES |
| 21 | TUG21 | GGGGAAAACACACAATAATTTCTCCACCAAACTTGATTTCACACACACACACACACACACACACACACACACCTTATACTCATTCACACTACAGTTCCCTCACCGCCGCTGCTACCATTTCATCGATTCACACACGATGGTACATTGTGCCGATCGGCTCACCGCCGACAGCTTTTCCTCACCGCCGCCGTGTGGTTTCTCCTTCTCTGGTGGCCGATCGTTAGCCCCTCTTTTCCTCACCGCCACTGTTGTGCCGATGGGCTCACCACCACCGCTAAAGCCAACCGTAGTTATCAGAAGGATATATCAGCTTTCTGAGAGGCCAGAGATTCCAATGCCAATGCATTAGTTGCTCAAGAGGATGGTCAGGGGAGTGTGATTTCTGGAGAAGGTTATATGGAGTGGGCTAAATGAAAGTCCTTAAGCCAGAATCCTAACAAAGAAGAGTATTGAATGTTTAACAAGCAAATTGTGAATTTTATGGGTTGTATAGATATAGTATGGTTTAACACTAAATGATGTTGCTTTGCATGATGGCAGAGTGGGTGTATGACAGCTCTTCAGGCTATTATGGTAATCAGAAATAATGGTTGTATCTATGATCCAAAGTCTCTCTTCTACATACACGTGATGCTTAAGGTAAGGGTGGGTGACACCGAGGAAAAACGC | CV014782 | YES | NO |
| 22 | TUG22 | GGGGCAGCTTCAGTTCATCTCTACAGTCTAGAGTTCCTTCCCTACCATATATATAGGCCATACTTGTCCTCTATTTTCTCATCAACCTAATCAGAGAGAGAGAGAGAGAGAGAGATAGAAACTTGCTCTTGCAGCTTTCCTTATGGKGTCTCTTGTWCTGAGCTCTTTTCTCCTCCAGACCGCAATGGCTGGTTCTAGTTCGTGTGACTCAARGTGCGGGGaTTCGGaTGCGCGAAAGCGGGTGTAcAMGAACAGGTGCTTGAAATACTGTGGGATTTGCTGCCAGGAATGgCAACTGCGTGCCTTCTGGGACTTATGGGAACAAGCAaCGAaGTtGttgCCCcTTttGgCTtAaCcAGGGggACAAAAAtGAACTCCAAtGGtGCCAGCCCaAAGTGCCCCTAAAaTAACcACACCTTCATTTACTCATCAAGAGATTATTATGTATAAGGCTACTACTATAAACATATATAGGT | CV014703, CV014829 | YES | YES |
| 23 | TUG23 | GGGGGAGCTTACAAAGAGTCAGAGCTCTCTCTCTCTCTCTCTCTCTCTCTCTGCGCTTCCAAGGGCAGTGACGAAGAGTTCGAAACCGATCTTTCTCAAATTTTCACTTCTCTGCTTTCTCTCTATCCTCTCTTCGGCACTCGCGATCACGCCCTGGACGGGTGATGGTTATACCATTAAGGGTCGGGTGCAGATTCCAACTCCAGGTCTTGGTGCAAAAGGATTGGGTGTTCTGGCAAAACTATCAAATATTCGGGTCATCCTCAATGGTGGCGAGAGGGTCACTTTCCTCAGGCCTGATGGATATTTTTCATTCCATAATGTCCCAGCAGGTACTCATCTTATTGGAAGTGTCTGCGATTGGGTAATTTCTTTTCTCCAGTTCGTGTTGATGTTAGTGCCAGAAACCCGGGTAAGGTTCAGGCAACACTCACAGAGATCAGGAGGAGTCTGAATGAGTGGGTTTGGGAGCCTTTGAAAGAGGAGCAGTATTATGAGATGAGGGAACCTTTCTCCATAATGTCTGTAGTGAAAAGCCCAAGGGGTTGGATGATGGGTTACATGGTGATCGTGGTTGTTCCTAATTGGCCCAAGCT | CV014631 | YES | YES |
| 24 | TUG24 | GGGGAARRRSATCAMACTCACTACAGCRGCAACCGCACTAAGTTTCTCTCTCTSTCTCTCTYTCTYTMAAAATCAAATCATACACTTTGAAGYGATGGCAAGAACGAAGCAGACGGCGAGGAAATCGACCGGTGGGAAGGCGCCGAGGAAGCAGCTGGCGACGAAGGCGGCACGGAAGTCGGCGCCGGCGACCGGAGGAGTGAAGAAGCCCCACTAGATTCAGGCCGTGGGACGGGTGGCGCTAAAGGGAGATCCGAAAGTACCAGAAGAGCACGGAGCTTYKGATCCGGAAGCTTCCATTCCAGAGGYKGGTGAGGGAGATCGCTCAGGACTTCAAGACYGACCTTCGTTTCCAGAGCTCCGCCGTCGCCGCCCTCCAGGAAGCCGCTGAAGCTTACCTCGTCGGCCTCTTKGAGGATACTAACCTTKGCGCTATTCATGCCAAGCGTGTATACCATCATGCCCAAGGGACATGCAGCTTGCTCGCAGAATTCGTGGCGAGAGGGCTTAGAAATATCTTTTMATTCAGTGCAACTTGTAGTAGAGATSCTATCTCTTYGTAGTAGCTAGTTTWKGTAATKGATTTCATCTATWCATGGCTGTGGATCAATKYARTGTTARTGGTCAACYCTGGACGAAGATCGTAKRCTGGAACCATTKTCTCGCAAWWWGGCARCACAA | CV014628,CV013804,CV014099. | YES | YES |
| 25 | TUG25 | GGGGATATTCTCTCTCTCTCTCTCTCTCTCTGGCGGGTTTTCGATCGACAGATCATCAATAAACGATTATGAGTTCTACAGGCACAACAAAAGGAGGCAGAGGCAAGCCCAAGTCCTCCAAATCGGTCTCCAGATCTCAGAAAGCCGGCCTCCAATTCCCCGTAGGCCGAATCGCCCGTTTCCTCAAGGCCGGAAAATACGCCGAGCGTGTCGGCGCCGGTGCGCCTGTCTACCTCTCCGCCGTTCTCGAATACCTCGCCGCCGAGGTTCTTGAACTTGCAGGGAATGCTGCGAGGGACAACAAGAAGAACCGGATAGTTCCAAGGCACATACAACTTGCTGTGAGAAATGATGAGGAGTTAAGCAAGCTTTGTGGGAACAGTCACTATTGCAAACGGGTGGTGTTCTGCCAAACATTCATCAGAATTTGTTGCCAAAGAAGGTTGGGAAAGGGAAAGGCGAGATTGGATCTGCATCACAGGAGTTTAAGGGTTTATGGTAGTGGTTGGATTATTTATATAATTAAGGTTTTCATGTGGTTAATTGATGGGATCAATTTTGGTGCTTTGTATTATTGATCCCTGTGTTGTGGGAATTTACATTTGAATATAGAAGAACAATAAATTTGTCGGAAA | CV014563 | NO | NO |
| 26 | TUG26 | GGGGTTCAGTAACGGTTTCAATTTGATGGAACCGTAAAAAAGGGTTCAATTCCTCATTTACTCTAAAATCGGTCCAATCCAATCCCTGTACACCTATTCACTTACCAAACCTGCTTCTACATATATGTCTATGGTTTTATTTATTCATACCCAAATCAGGTTCTCTCTCTCTCTCTCTCTCTCTCCCCTAAACAAAAGTGGACAAAAAAAGAGTTTTCACAATTCCCCAAGTTGCTGAACACAAATCCAAGGAGGATGTGCGTGGTTCATCATCAAGTGGCAGAGATATGGGACGTGACGAAGTTTCTGGAAGAAACACCCAGGAGGAGAGGAGGTTTTGGATCGAATCAGCCGGAAAAGATGCAACAAAGGACTTTGAGGATATAGGACACAGCAAGGCTGCTAAGAACTTGATCCTCAAGTATCAGGTAGGGTACCTCCAGGGCTACAACATTCAAGAAGGCAATGACTCCAATGTTGAGATGGCTTCTAACAAGGAATCCAGGCCCAAAGAAATGAAAGCTTTCGTGATCAAGGACGATCTGGTGCCCAAAGTATGCGGCTTTTCTCGAGTTCTTTCGATGCCTCTGATAGTTGCCGATGGCTTCTTCAGTTACCGGTACCTCACCGGAGGCAGCCCAGTTCAGCTCTTTAAAACATGAATTTGGGATTTGGGGAATGGTAATCACTGGTCTTAAAATTACTACTGATGATTAAGCTTGCATGATGATGTAGAAAA | CV014520 | YES | NO |
| 27 | TUG27 | GGGGATAGTACAAACACACAACTTCATGAGAGAGAGAGAGAGAGATAAAGAGAGGAGAGAGAGAGAGAGCAATGGAGACATCCCAGGTGATTAACATGGAGAAGCTAAAGTGGTGAAGAAAGAGGAGCCACCAGTGGAGCTCATCAAAGATGCTTGCGAGAACTGGGGCTTTTTTGAGTTGATGAACCATGGAATTTCTCATGAGATGATGGACACAGTGGAGAGAATGACAAAGGAGCACTACAAGAAATGTATGGAACAGAGGTTCAAGGAGGTGGTAGCAAGCAAGGCTCTTGAGGGTGTCCAAGCAGAGGTCACCAACATGGACTGGGAGAGCACCTTCCACCTGCGCCATCTTCCTCAATCCAACATTTCAGAAGTACCCGATCTCAATGACGAATATAGGAAGGTTATGAAGGAATTTGCTGCAAAATTAGAGAAACTAGCAGAGGAGCTACTGGACCTGCTATGTGAGAATCTTGGGCTAGAGAAAGGGTACCTAAAGAAGGCCTTCTATGGAACAAAGGGTCCCAACTTGTGGGACCAAGGTTACCAACTACCCACCATGTCCCAAGCCAGACTTGATCAAGGGCCTCCGAGCCCACAACCGATGCCGGCGGCATCATCTTGCTCTTCCAAGATGCACGAGGTCAGCGGCCTCCAAACTCCTCAAGGA | CV014448 | YES | YES |
| 28 | TUG28 | GGGACCAACATTGCTCTTACCTCCTATTGGATGAAGAAAAATGGTAGGTTCAAAGACATCTTCTCTTCTAATTCTACTCATCATCATCATCTTTTTCTTCTTCAATATTGTCTCATCTCATAAGGTCCCATGCCCTCCGGCGAATCCTCCAGCCAAGTGCCCAAAAGACATTCTCAAGTTTGGTGTTTGTGGTGAATGGTTGGGATTGGTGACTGAAGTCATTGGGACTAAACCTAGTCCCCAATGCTGTACAGTGGTGAAAGGTTTGGCTGACCTCGAAGCTGCATTCTGTTTATGTACTGCAATTAAGGCCAATGTGCTGGGTGTTATCAAGCTTGAAGTCCCCATTGCTCTTAGTTTGGTCATTAATGGGTGTGGAAAGAATGTTCCAGAAGGATTTGTATGTGCATAGATATGATGCATGATGATGTGTGTGTGTGTGTGTGTGTGTGAGAGAGAGAGAGAGAGAGAGAGAGAGAGCAGAGTATGATTGTGTTTTGGTGAGTGATATGAACTGTACTTTATGATGTGGTGGCAATGATTGTCCACAAAAAAAAAACAAAAA | CV014397 | YES | YES |
| 29 | TUG29 | GGGGACTAAAATTTTAACAAAACAGAGCCTTCATAAGCTCTCTCTCTCCTTATCTCTCTCTCTCTCTCTTTCTCTCTCATACACTGTCCCTCATTTCACTCCTCGCGTCTGTCTTCTGTCTCGATTTCTAGAGAGAGAAGATCGATTGATATATATGAGAGAGAGTCAGGCGGCCGTGTTCGGACATCAATGGCGTTCGTATCGTTCCTTGGGAGAGTCCTGTTTGCCTCTGTTTTCATACTCTCTGCATGGCAAGAGTTCAATGAATTTGGCGTTATGTGGCCCAGCTGCGGAGTCCCTTACACCAAGTTCAATGTATTCTCAAAACATTTGTCAGTTCAAACTGGGCTGCAAGTGCCAGATATAGAATCGTTTTATTCACAGATCAAATTTCTAGTTTTGCAGCCATAGCTATAAGGGTCTTGGAAGCCTTCTTTTCATTTTTGGCAGCTCTCTTGGAGCTTTTC | CV014365 | YES | YES |
| 30 | TUG30 | AACTGCTTTCAGTTTCCCCTGTCATGTTCTGTCCTCTATGACTCTCAAATACTAACCAAGATGTCCGCAAATTGCTTCGAGATAGAGCAGTAGTTGAAGTAACAGCTAGCACACCTATAATCCCTGCCCTTTTAAGAGAGTTCTTGATGTGATTACTCCCKSSCTCTCTCTCTCTCTCTCAGAACACTCACAGYGTCKCGCYCTCTTTACATCTCTCTCTCTTCTCTCTCATTGTTTTAGGTTTGAATCGCAACAAGGAAGATTTCTCTGTTTCTCTCTCTTTCTTGAAGCTGTCCAAAGTGTGAAATTGGGAAGTAGAGCAGTGTAGAGGGATGGGTCAGATCCAGTACTCCGAGAAGTACTTCGACGACACCTACGAGTACAGGCATGTGGTTCTACCTCCTGAAGTGGCCAAACTTCTTCCCAAGAATCGCCTTCTCTCTGAAAACGAGTGGCGTGCGATTGGGGTGCAGCAGAGCAGAGGGTGGGTCCACTACGCAATCCATCGACCAGAGCCACATATTATGCTCTTCAGGAGGCCTCTGAACTATCAGCAGCAGCAGGAGAATCAGGCTCAGCCAGACTTGGCTGTCTAGTGAAATGTCACTCTCTGTCCCTTTCTAAATCCTTCTTAAAATTACTATATCTTCATTTCTGATAGAAATGAATTCTATGATGTGTGTTACCTGGGATTTGAGGTTCGGATAACTTTGGAACTGCTTTGGTATGTGATTCTCTTAACTCCGGAGAATTTCATACGGAACCTTTTGTTGGTTGACTCTGATGTCGCTAGTACTTTGGCAGGTAATAGGTGTATGAATCGGGGAACCTGGCGTATTATTGTTGCATTGGGTGTGGATACATGCTGGGGTAT | CV014324,CV013782 | YES | NO |
| 31 | TUG31 | CTTTCCTCACCTTCTATTGTCRCCAAGRAARTTRCGGATCTCGATGGACTTGTTKSYRTTGGTGATGCTAGCGTTGATCGGAAAATGAGCGTAGACGAAACGCATCTTGTATCGGTAACCCTTGGTGACACCGTTGATGAGGTATCGACATGGCTGAGCGCGGTGCGAATGGCGGCGGTAGTTTTCCGAGATCCGAACCAAGCATCGATCTTCAGCTTCTTCTTCCCTGTTTCCTCATCTGTGATCAGCTGGAAATCCAGGTTCAGATGCTTGAAATTGCGAGTCAGCTTCCCTCTAGGTCCCTCCACTTCGATCATCTTCGCCTTCACTTTGATCTTCACCCCATCTGGGATGTCCATGGTCTCCGACGACAAAATCGTCTTCATCTCGCACTCTCTCTCTCTCTCTGTTTGTTTGTGCGAAGATCGGGGAGGCGAGCAACCCCCCTAGCAARGKGACASRCAWATAWMACATCCTCTCTCTCTCTCTCTCTCTMMACCCTTCCAATGGATTCAATAGCCCACCACTACCACTACAGCTCACCTCCTCCTCCCCACCACTACTAAGAAATGGCTTTGATATAGTTCTAAGGAAAGGAGCTGAATAAAATAAAGAAGGCCAGTGTTTGCAAGCTTTTTTAATTAGAGAAGAGAGAGAAGGAAGGCAACAAGAAGATGAGGACGAGGAAGAAGAAGTAGAATAAGCCAAAAAAAACAGCCTGGTGTGTTTGATTGTATGTTGATAATGTTGTAATATTGCGGTGGTCCATGCAAGTATGCATGTATGAATGTTTTGACCCCTTGACAAGTGTGCAGAGAGGTCATTTATATAATAAAAAAGTGATAATGTTTCATCCTTTCACAAAAAAAAAAAAAAAAAAAAAAAATAAAA | CV013965,CV013919,CV013731,  CV014314 | YES | YES |
| 32 | TUG32 | GGGTGCTGCGATAAAAGATAGGATGGCAAGCCAACCGGCCATGAACAATGCGAAGCCTCCGACAGTGAATATCCGTGGACAGCCTGTTGCACAGAGCGGTGGCTGCCGCTCTTCTTAAGTGGAAAGGGGTAGGTGGGTACTAATTGTCTATGTACGACTCTCTGCCTGCGCTTTGGCTTCTCTCTCTCTCTCTCTCTCTCTCTCTCTCTAGCATGTGAAAGTACTATTTTCTTCACTGTCTTTAATTTGAAGGAAAAGCATATATATATACTCGTTTAACTCCAGACAAACTTTGTTTAACTATGCTTTTTTTTTTTTTTAGGTCTGTTTGGAGATTTGCGTATCCATTTTATTCTTTTGAATATATTTGATTATGAAAGCGATGTACAAATTAAGAATATGTCAATTAATGTTAGAAT | CV014223 | YES | N0 |
| 33 | TUG33 | GGGGAGAGAAGAAAAAGAGAAGAGGTGATGGCGGCACGACGAAGGTGATGGTACTATGATGATGGCACAACGGTGGGTCTCTTCGACCCTCTTCTCTCACCAGATCTGTGTGTATGCGCACAGATCTATACACAGATCTGTCTCCGAATTGGTTTTGATTTGTGGGTTTGTTTTTTATTTTGATTTGGTTTTGATTTGTAGATTTGTTTTTTATTTTGATTTGGTTTTGATTTGTATACACAGATAAGTTATTGTGGGTTTGTTTTTTAATTTTAATTTGGTTTTGGTTTGTATTGTGTATATACACAGATCAGTTGATTTAGTTTTAGAACTTCATTTTGGTCATGTTTGAGAGAGAGAGAGAGAGAGAGATGAAACTGTGTAGAAGGCAAAGAAGGGAAATGGGTATTAAAAAAAAAAAAAAAAATCCAGACCAAAAAAAAAAAAAAAAAAAAAAAAAAAG | CV014134 | YES | YES |
| 34 | TUG34 | GGGGATCTCAAAGCCGTCTTCGTCTTCTTCTTCTTCTTCTTCTTCTTCTTCTCTCCCAAAGTGTTGCCAAAATTCCATCTAGGGTTAGGGTTTCACAGAGAGAGAGAGAGAGAGAGATGGGAGTGTTCACATTTGTGTGCAGGAGCTCCGGCGACGAGTGGAGCGCGAAGCAGCTCTCAGGCGACCTTGAGGCCTCGGCCGGGTCCACATACGAGTTGCAGAGGAAACTTGTTCAGGCCTCTCTCTCCTCCGATTCCTCCGGCGGTGTTCAGTCTTCTTTCTCTCTCATCACTCCCTCTTCCGCTGTTTTCCAGGTGATTATTGGTGGAGGTGGTGGAGGTGGAGGTGTTGCTTTTGCTGCAGCTCCTGCAGGTGGAGGTGGAGCCCCAGCTGCTGAAGCACCTCCAGCTGAGGAGAAGAAGGAAGAGAAAGAGGAGAGTGAAGATGGAAGATATGGGTTTCTCACTTTTTGATTAGTGAATCATCTATATACAATGCTTTCTTATTTACCAAGTTTTGAATGTGTCGGCCAACTTTGATAGGGGTTGAATGTTGGCTTTTCATCAGTCAAGTATGAATCTTGTGGTGGTCTTTATGATCTSTAGTAGACAACTCACATTTGGTGGTTTTAAARCTGTGTTTTGAACTKGGCTTTGCTATATCTAATATGAGCTAGTGT | CV014184,CV014102,CV014769,  CV014072,CV014391,CV013956 | YES | YES |
| 35 | TUG35 | GGGGCTCTCTCTCTCTAAAGCAAGTTCACCAACACTATCATTCGCTCTCTCTCTCTCTCTCTCTCTCCATGGCTTCTACTCTCATTATTACAATGGCAAAGCCCTTTACTTCTCACAGCACCAACCTACCCTCGCTCTCTAACCAGAGACTGCTAGGTGGGCGTAGAAATTCCCTGAGAGTCAGTGCGATTGCAAAGAAATTTGAACCCACAAAGGTTATACCGCAAGCTGATAGAGTTCTGATTCGTCTGGAGGAGCTGCCTGAGAAATCAGCTGGTGGAGTTTTGCTACCCAAATCAGCTGTCAAATTTGAACGGTACCTTATGGGGGAGATTCTTTCTATTGGTGCTGAGGTGGAGCAAGTGGAGGCAGGGAAGAAGGTTCTTTTCTCCGACGTAAGTGCTTATGAGGTGGATTTGGGAACAGATGGAAGGCACTGCTTTTGCAAAGCTGGTGATCTGCTGGCCGTGGTTGAGTAAAGATGTTGATGTTGATGTTCAACCTCTTTAGAGAATTAACTAGCTGCTTTGCAGCCATTGGTTTGTGAACCTGTGTTAATTTGGGAGCAATATTGTTTGTTGAATGTTTACTTTGTTCAGAAACAGGGGAACAAAAATTTTTTGATTC | CV013984 | YES | YES |
| 36 | TUG36 | GGGGGAGTTGAGGCCAGCAAGTAAGAGAAGCTTCAATCTGTTTGGTGAGAGAGAGAGAGAAAGAGAGAGAAAGAGAGAAGCAATGGCATCACTCAAGCCCGAGAAACCTGTTGGTACTCAACCCACCGCCCCGGCCAAGAAAGAGCCTTCCAAAGGTTCTTCTAAGAAGGTCGGGCCGAAACCTCAAGAGCCTAAGAAAAAGTCATCAGGAACCAAGTTAGCAACAAAGAAGAAATAAGGACGATAGCACTGGCTTGTGATCTATAGCTATGTAGCCCTTTTTGGGATTGGCATAATAATTAAATAAAACATAAATATTTGACAGGAAAAAAAAAAAAAAAAAAAAAAAAAAAAAA | CV013972 | YES | YES |
| 37 | TUG37 | GGGGAATGAGAAGGTGCTGGTATCAACCTTCAAAACATATGCTTATATGTGTAAGTATTTGAGTGCTTCCTTTTCTCAATATAAGAATTACAGCAATGTTAGTGTTTGCTCTTTGCAAATTGAGTTATGTTCTCTATGTGAAGGTATATGTATATCTATCTATCTATATATATACATATATATCTGTTTTGGATTGTCTGCAAAAAAAAAAAAAAAAAAAAAAAAAAAAAA | CV013954 | NO | NO |
| 38 | TUG38 | GGGGATCACAAGATTGACATACACACGCACAGAGCTGACGCTGCAAAACAACAGCTTCAATTCAATCCTCTCCACTTTTAGAGAGAGAAAGTAGATAAACGAGAGAGAGAAAGAGGGAGAGAGAGAGAGAAGAAGTCGTGGATTTCAAGGCTGCTTCGGCACTCCTCCCGATACGATAAGAAAAAAATGAGTACATTGGACGTAGCTAGAGCAGAGCTTGGCCTTGCGGTTCTGTATTTGAACAAAGCCGAAGCCAGGGACAAGATATGCAGGGCAATACAATATGGTTCAAAATACTTGAGTAACGGAGAGCCTGGCACAGCCCAAAATGTTGACAAATCAACTAGCTTGGCACGGAAAGTGTTCCGTCTTTTCAAGTTTGTCAATGATCTGCACGCGCTCATTAGTCCAACTTCTCCTGGAACACCCCTTCCCCTTGTTTTTGCTGGGAAAGTCCAAAAATGCACTGTTGTCTACTTTCTTGTATTCTGGATCAAATTGTCTGGCTCAGTAGGACAGGCATTTATAAAGAACAAAGAACGTGCAGAGCTAATTGGCAGGATCTCTCTTTACTGTGGATGGGTTCCTCAATCTTGCACTTACCTTGGGTAGAGGTATGGGGAGCTTGGAAGCTTTCTGCAATTCCAACTGGAAGAAA | CV013948 | YES | N0 |
| 39 | TUG39 | TTCGCAAATTAATGGAAGTCATATATTCTTCATGGGTAGCCAAAGTAAATTACAGTGCTCAGTTTTTAAAACATTCAGTCATTATTGGGTAACATAGAAATGCCAACAGATTTGACATTCAATTCCAACAAAAGAAATCATATTGCTGAACTCCTTTCTGCATGTAGCATTGATAAATTTACCTCTTGTGCCTCTGCACAAAGGGAGAGTTCTTCGCATCCTCCTCGGTGAGACCGAGAGAGAGCTTGGTGATGACAGTACCGGTTACGAAGAAACCGACCAAGAACGGCCAGTTCCTACTCCATTCTCGCCGGAAGAAAACGGGTCACACRRATCGSATCCYCAAACATACCTCTCTCTCTCTCTCTCTCTCTCTCTCACATGGCTCCTCTAACCTGHTCTCACCACCCACCTTCCAAGGCCTCAGAMCCACCACCAAATCCCCACTCTCCCTCACCAAATCCATATCCACCGTCAAATTCACCCCCAAAAAACGATGCAGCGGCGCCATTAAAGCCGAATTGAACACATCTCTGGTCATCAGTCTCAGCACTGGTCTGTCCCTGTTTTTGGGCAGGTTCGTGTTCTTCAATTTCCAGAGAGAGAATGTGGCCAAACAGGTTCCCGAACAGAACGGTATGACCCATTTCGAAGCTGGTGATTCTCGAGCCAAAGAGTATGTGAGTCTCCTCAAATCCAACGAGCCAGTTRGGGTTCAACATCGTCGATGTCTTGGCTTGGGGTTCCATTGGACACATCGTTGCTTACTATATACTTGCTACCTCTAGCAATGGATATGATCCCAAGTTCTTTGGATGAATTTGAATTTGAATTTGAATTTGGATTGTTGTTGGGTTGTGCTAGTAATTCATGTGTATAGGTGGGGAGGCTACAATTTWTCTMTGTTGTTCTTCATGTTTTCGAATTAATTTGAAGCTTTGCTGTGTGTGTGGTSSWAGGAGGGGATGTGTGTAGTCTGGATGGTTATTTAGATCTTTTTCAAAAAAAAAAAAAATTG | CV067152,CV013915,CV013656,  CV067044 | YES | N0 |
| 40 | TUG40 | ATGAACACATGACATAAGATAACCGAAGATGAAATGGAAGAGTGATATCAATTTCCCATTAACAARGAATTTCCTTCYAATATATAACGATATTAAGGCAATAGCTTCRCATCTATACCAAGTTTGAGTGATCAAGCAATAGACATCAATGGATTTACCAATAGAAATGCAACCAAAACGATGAAATGTCAARCACGTTTCCCACTATTGGGATTGGACTTTGTGCAAAGGATCCATCGTCCCTTGGCACGTCTACGCTTCAACACYGCTCTTCCACTGGTGGTGCTCATTCTTTTGCGAAAGCCATGTGTCCGGGCCAGAGACTTTCGAGATCTGTTTCTCTTGGTTTGACAAAGAGTATACTTCCGRGCAGTCACTACTAGGCCACGGCCTTTCTCTCTTCTTACCCCAGTACTAGAATTCAAGTTCAAGCCTAATGACAAACCTGAAAAAGAAGAACCAGAAGAGAGAGAAAGTGCAGTGGCAGGGACGAAGGAGCAATGAAGGAACCCAGAACGACTAGGTGCATTTGTGGCCCCTTTCAAAGACCCCACAGAGCTCAATCTTTGGCTCGAACCAGTAAGGAGAGTAAGAGAAGCTGTTGGAATTCGAGTTTGTGAGCAAACCCATGTTCCCAGTGATACAAAGCCATTCTCTCTCTCTCTCTCTCTCTCTCTCTCTCTGTTATCYKCACCKMTGCTTCASMSMAAAAGAAGCCAAAAAAAAAAAAAAAATCCAAAACGTCGTTTTGGAACTCGGGCATTCGACCCTTGTGTATTTTCTGAGCACAAATCGGAAAGTTGTGTTTGGTTTGTGTTGTCATGTTGTCAATTTTTTTCACATGCATTCACATGCAATGTTACTGCATACACATTTGTTTTGTGTGTTTTACAACACAAACACACAACACAACACACAACACACACACACAAACACACACAAACAACACACACACA | CV013872,CV014206,CV013886 | YES | NO |
| 41 | TUG41 | GGGGACGACCACAAAAAACCTTTCACAACAGATCCACACCAAAAAAAAAAAAAAAAGTGTCCTTTTCAATCATCATTCTCTGTGTGTGTGTGAGAGAGAGAGAGAGAGAGAGATCAATCGGGGATCGGTCTGGTATGGCAGAGGCGCAATCTAGCTACCCCTACCCGCCGATGGCAAACCCAGTGGCCGTGATAAGCCCACAATATTGCGCACCGTACCCAGTTGATCTAACCATCGTGAGGAAGCTCCTGACCATAACAGAGGGCAACTTCGCAGTCACCGATGTCAATGGAAACATCATGTTCAAGATCAAAGGCAAGATTTTTAGCCTCCGCGACCGCCGAATCCTCCTCGACTCCGCCGACAACCCCATCCTCACTCTCCAACAAAAGATATTGACTGCACATAGGAGGTGGCAAGTGTTCAGGGGACAGAGCTCAGACCCCAAAGATCTGCTTTTCAGGTGTTAAAAAGTCGTCACTTATCCAATTCAAGACCGAATTAGATGTGTTCTTGGCTGCAATACTAAAGAAGAAGTTGGTGATTTCAAGGTTAAGGGAAGTTGGCTTGAGAGATCATGTGCCATACATCTTTGGAAATACTCCAACCATCATTTGCCCAGAATGCACAGAAAACACGGGTGTTCAAAAGCGTTGTTCTTGGGAAAAAGATACCGTTTGTGGGTGACTGGGTTCTCCCAACGGTGG | CV013881 | YES | YES |
| 42 | TUG42 | GGGGGTCGTAGCTCGCAACACAACACCACCAACAAGAGAGAGAGGGAAAAAAATACTAGAGAGAGAGAGAGAGAGAGAGAACGGAGGACAGCGTCGTCATTTCGGAGGGGACGGATCTCATATCGTGTGTGTGGAGAGAGAGAGAGAGAGAGAGAGTGAATATTAGAAAGAGAAAGGGAGACGCAGAGAGTGTGTCGTTGGAGAAGAGAGAGAGAAGCATCTGGAATCTGTTTGATTTGTGACTGGAGCATTTTGATTTGTTCTAGAAGGAAGGAAGAATGGAGAGAAGCACTCCGGTGAGGAAGTCTCACACATCCACTGCAGATCTACTCACCTGGTCTGAAATTCCTCCCTCCTATTCCCCCTCCACCGCCTCAGCTGCTCGTTCTCACCAGCCATCGGATGGGATCAGTAAGGTGGTGTTCGGAGGTCAGGTTACGGATGAAGAAGTCGAGAGTTTGAACAAAAGGGTCGTAGCTCGCAACACAACACCACCGAAAAAAAAAAAAAAAAAAAAAAAAAAAAAA | CV013870 | YES | YES |
| 43 | TUG43 | GGGGACCCTCTTCTCTAAAACCCTAATTTTACCCACTTTTCATTTCCTTCTCACCCCTACCTCTTCTTACCTTCCGCCGTCCCAAACCCTAGAGAGAGAGAGAGAGAGAGAGATGGGTCGTGTGATCAGAGCTCAACGTAAGGGTGCTGGGTCCGTATTCAAGTCCCACACCCACCACCGCAAGGGTCCAGCTCGGTTCCGCAGCCTCGACTTCGGCGAGCGCAACGGCTACCTGAAGGGCGTGATCACCGAGATCATCCACGACCCAGGTCGCGGTGCGCCTCTGGCTCGAGTCACGTTCCGTCACCCCTTCCGATTCAAGCACCAGAAGGAGCTCTTCGTCGCCGCCGAAGGCATGTASRSTGGCCAGTTCGTTTTCTGCGGAAAGAAAGCCAATCTCRTGGTTGGCAATGTTCTTCCTCTCAGATCTATCCCTGAAGGTGCTGTTGTTTGCAACGTTGAACACCATGTCGGTGATCGTGGTGTCTTGGCTAGAGCTTCTGGTGATTACGCTATTGTTATCAGTCRTAACCCAGATAATGGYACCTCAAGGATCAAACTCCCATCAGGAGCGAAAAAGATTGTGCCCAGTGGGTGTCGGGCCATGATTGGCCAGGTTGCCGGAGGAGGACGTACTGAAAAGCCCATGCTCAAGGCTGGTAACGCATATCACAAAGTATCCGTGTCAAGARGAACAGYKGGCCCAAGGGTWCGTGGTGTGGCTAATGAATCCCGTGGAAGCCATCCCCATGGTGGTGGTAACCATCAACATATTGGTCATGCCAGCACTGTTCGCCGTGATGCACCACCTGGGCAAAAGGTCGGTCTTATTGCTGCCAGGAGGACTGGTCGTCTCCGTGGACAAGCCGCTGCTACCGCTGCAAAGCTGATAAGACTACTTAGTGAGATTTTTAAGGATGCTTTGGCTTTTTATTTTCGTTATTTTCTTTTCCAGTCTTTATGATTCACGTGGATATGGAGTTTCGCAGGATCTAGAGGTAATATCTGTGGGGAATCTTAGAANATGTGTTGGTTGAATGAACCCCTTTCTTGGAAGTATGGGTATTGCTATGTTACATTGGTCCCCCATTTTAATTT | CV013831,CV013821 | YES | YES |
| 44 | TUG44 | GGGGGTTTGGTTATGCCATTTTCGCAAGGGAGTCTCTCCCTCGATTCGGTCTCCCAAACAGATAATCCACGTGCGCCTTTGGTCCAAATCCATCACCAACCCACGTCGGTTCTTCGCAAATCTCCCCTGACCCATCTCTCGAGTCTCACCCCATAGTCCCAATTTATGGCTTCGCAGATTCACAGGTAAATTCTGGGGGATGGACGTAAATTGTCACGTGGTGTTGGGAGTGTTGCTGAACATGCACCATACAGGTACGTATGTAAGAGACTTTGGTACGAGGGTTGAGAGGTGTGAAGAGAGAGAAAGAGAGAGAAAGAGAGTATCGGGGAGATGTCGAATCAGGTGGTGAAAGTGAGGAGAGAGACGATGGGTCATGCATGACTTGTCCGCTTTGCCACGAAGCTCTTCAAAGAAGCCACGACCATATCTGAATGTCTTCATACTTTGTGTGTAGAAAATGCATATATAAGAACGCTTTTAGACGATGGAAGTCGGCAATGTTGTACCAAGTATGCAAGCATCAATTCGGGTTGTGTGCCGATTGGAGAAGCTGAGGCCAGACCACTAACTTGCAGAGATGTGAGAATCACATGATCTTCCCTTTCAAACAGAAGAACAGGGTGAAGGCACCAGAAGTTGCAGCTTCTGTAACATTACCAGTGAGAAGAACAGGAGCAGATCTACTGTCATCATTGGTGGTCAGTACTCCCAGAGTAT | CV013820 | YES | YES |
| 45 | TUG45 | GGGGGATTGTTGAAGTTTCTCTCTCTAATTCATCTGCAATTGTAGAGAGAGAAAGCAGATATTGAGAGAGAGAGAGAGAGAGAGAGAGGCCTGAGGTATACTTAGAGAAGTTTGGAAGATATGGGTGAAGAGAAGAAAGAGGAGGAGAAGAAGAACGAAGGAGAAAAAAAGGAAGAAGGCAAAAAAAAAAAAAAAAAAAAAAAAAAAAAA | CV013818 | YES | YES |
| 46 | TUG46 | GGGGGTTCAGTCGCAGCAAAGCCTCTCTCTCTCTCTCTCTCTCTCTCTCCAAAACACACACAGAGTAACAATGACTGSTCAGACGCAAGAAGAACTCCTCGCCGCTCACCTCGAGCAGAAGATCGATCTTGAGAAGCCAGTGATTGAAGATGAAGATGATGAGGACTGATGACGACTGAAGACTGATGACAAAGACTGAACGACTGACTGTTGAACGGACAACGGAGATGCAAGTGGTGATGACACAAGCAGCGTGTGTCGAGATCGAAGGCCTGTGACAAGCTCTCAAAGGSCGCAAATGGTGACATTGTCTCTGCCATTATGGAACTTACAAACTAAGAGACAAGGTCGTATGGTGTGTATTTCAGTCTAGTGAGTATGCACTTTTTTTTTTTTTTTGTTGTCTTTCTTTCAAAACCATTTCTAGAGGATTTATAATGTTGAGGCTTTGGATTGGTAACCATTGTGGTGTTTACTCTTTGGTATGTTCTGTGTGTATTAGAACGAGGTTATTAAACAGGCATGGTCCAAGTGTCATGATATAAAGTTATCATATTGCACTCATTTCACACATTGAGAAAATT | CV013791,CV013892 | YES | YES |
| 47 | TUG47 | AAGGCAATTTGATAAGGCATATGGAGGAGGATGGCAATGCATAGTGGGTACACGATTTGGGTCGTGTGACTCACGTGCTATGGCTGTTATCATCTATTTCTGCATTGGCAGCCTTGCCATCTTGCTCTTCAGGGGTTCTGCAGCCCCTGAGGCCTCCCAATTAGCTTCCTKGGRGAGCAGTCARAGCTYSCAACGTCTCTCTCTCTCTCTCTCTCTCTCTCTCTCCAATTTTCACCATTGTTACTCAAGCTTTGAGCTTAAAGTGGGAGTCCTTAGAGTGAGATTTTGCACAATCTTGTAAACGGAAGTTGGAGAGCTCGCTGCACTCTTCGCACCGGTAGTCTCGGATTCATGATCGGGCGACGCTTGCATTTTCAAATTTGTTTTTCTTCATATTTGAGGAAATGTTTTTCGAATTTGAAGATCAAACCATGGGTTTTACCATTTCATAAGTGTAATGTAATGATGGATGGTACTACTTCACTTGTTGTTTGGCCGAGTGCTAGACAATAGCGTGGATGGACTTTCGGGAAGCAAGAGTTTCTTAATTATGTTTTGTTGAGTGTATGTTGGTGTTTGTAAAGCCAAGGGCTTAAGATGTAATGAGCGAGATTCAATTTGTGTAACCAAGCAATGACTTGGAAGTATGTAAGCCTACATGGCATTTAACTAGTTGTTAAACAGTAGACTCTGATGTATGAAAGTACGTTATTTCGTGAAAAAAAAACAACAAAAAAAAAACAAAA | CV013561,CV013781 | YES | NO |
| 48 | TUG48 | GGGGGGACAAGCTCGGGCAACCACCATATATATATTTAGAGAGAGAGAGAGAGAGAGAGAGAGAGAAATGGCTGCAACATCAGCTCCAGTGGGCTCTCCCTTCTTGTCTGGTGGGAAAAGAAGCCAGACATTGTTGTTGTCTGCAACCATTGGAGCTAGATCACTTGAGTGGCGGATGTGGTGGCTCCTAAGAAGTTTGTACTGGTGGCTGCTGCTCTTAAGAAGTCTTGGATCCCTGACTGTCAAAGGTGAGTGGCAGCTTCATCAACCCCGAATGGACTCGATGACTCGCTACCAGGGGACTATGGTAATATAGACCCTCTGGGCCTGAGGA | CV013757 | YES | YES |
| 49 | TUG49 | GGGGAGAAAATTTGAGTTTGAGAGAGAGAGAGAGAGAGAGAGAGAAATATGTCAGACGAGGAGCATCACTTCGAGTCGAAGGCCGACGCAGGGGCCTCCAAGACCTACCCACAGCAGGCTGGAACAATCCGCAAAAATGGCTACATAGTCATCAAGAACAGGCCTTGCAAGGTTGTGGAGGTCTCCACCTCAAAAACTGGCAAACACGGACATGCAAAGTGTCACTTTGTGGCAATTGATATATTTAATGGCAAGAAGCTTGAAGATATTGTCCCTTCATCCCACAACTGTGATGTTCCCCATGTTAACCGTACTGACTATCAGCTGATTGATATCTCCGAAGACGGTTTTGTGAGTCTTTTGACTGAAAATGGAAACACCAAGGATGACCTGAGGCTCCCCACCGATGAATCTCTGCTCACCCAGATTAAAGATGGGTTTGGTGACGGGAAGGACCTTGTGGTGACTGTCATGTCTGCAATGGGAGAGGAGCAGATCTGTGCCCTCAAGGACATTGGTCCTAAAAATTAAATGTGGTAGTAGGAAAAAAAAACAATCCTATGTTATCTAACATGGAATTTTATTTTATTCGACTATATGTATGTGGGTGTGAAGTGACCTGTTAATACTATGGTTTTAGCCATATGTAGTAAACCTGTTCGGGCTTCTCAGANGGGAAGGAAGAAGAGCTGGCTCTTGATCCCGGGGAGGACCTGCTACTACAG | CV013727 | YES | NO |
| 50 | TUG50 | GGGGATTCATCTCTGAACACACTATCTCTCTCTGTCTCTCTCTCTCTCATGGCGTCGCTGCTGTTAGTCGGAGTCGGAGCTCTTCCAATGGCCTCCTCCAAAGCTCTCACTCTCTCCCCCACGCTTTCGTTCTCTCGATCCCAATCTCTCTCTTCTCTCTCTCTCCCAACTTCTTCCTTCTCACTCTCAACCTCTCCATCACTCCCATTAATTTACTGTGGCAGAGGCGACAAGAAGACCGCCAGGGGGAAGCGATTCAACCACTCCTTCGGAAATGCAAGGCCTCGGAACAAGAAGAAGGGGAGAGGACCTCCGAGGGTTCCGGTTCCTCCAGRCACCACCCAAGAAGGACCGTTTCGACGACACCGAGAAGATCAAAATCGATATCGATGAGTCCCTCTTTTCTAGTTGAAGCAATTATTGCTGAATTTGTTTGTGATTTCTGGTAATTTCTCATCTTTTATGCATATCTTTTGCTGTCACAATGTTTTTAACTTTTGGTAATTGTTAATTTGCACTTTATCTTAATTTGCTTGTGAWRMMWWWWMWMAAAAAAAAAAAAAAAAAA | CV013700,CV013641,  CV013971,CV013717. | YES | YES |
| 51 | TUG51 | GGGAAAGTGCGAAACCAAACCAGACTCATCGCAGAAATCTGTATCAAAGAGTAGAGAGAGAGAGAGAGAGAGAGAAGCCATGGAAGGTCAAGAGGGTTCGCAGCAACCTCAGCTAGTGCTAGCTCACAAGCTATTCCTCCTCACCCAACCAGACGTCCCTGACATCGAGAAATTTCGTCTCCGCGAAGAGGTCCTGGACTTCGTCGTAGCCGATGATATGGTCGGGCTTTATGAAACCCTAGGCGCCAGTTCGTCCCTGGATTGGGATCTCTTGGCCTCGATGCGTGCCAAGATCGATGAACAGCTTAAAAAACTCGATGAAAAGATTGCTGATGCCGAAGAAAACTTAGGTGAAAGCGAAGTTAGAGAAGCCCATTTAGCCAAGTCATGTTTTTCATACGAATGGCGACATGGAGAAGGCTTGGAACAGCTCAAGTTAACAGAAAGCAAGACAGTTGCTGTTGGGCAGAAAATGGACCTGGTTTTCCATACGCTACAGATTGGCTTTTTCTATATGGATTTCGATCTTATTTCCAAGAGCATTGATAAAGCAAAGAACTTGTTTGAGGAGGGAGGTGACTGGGAAAGGAAGAATCGTCTACAAGGTGTATGAAGGCTTATACTACATGTCTACTCGAAACTTTAAGAAAGCCACCAGTTTGTTTCTTGATTCGAATCTCAACTTTCACAACTTTATGAACTTTTCCCATATGACCAACT | CV013686 | YES | YES |
| 52 | TUG52 | GGGGAGAACCAACCCAGTCTATACTCCTTCTTCACTGTGTTTTTGTGTGCGTGCGTTTGAGAGAGAGAGAGAGAGAGAGAGAGAGAGGATTGGATGGCGTGTGCTTTGAGTATGGTGAGTACTGGGTTCTCCTACTACAGCGGATTGGGGCTGAAGAAGCCAAGCAGAGTCGGCGGAGCTGTGACCCTCAGGAGGCAAAGGGCGGTGGTGAGAGCGGAGTCTATAAATCCAGAGATCAGAAAGTCCTAAGAGAAAGTGGTTGACTCTGTAATCGTCTCCGAACTTTCCAAGCCTCTCACCGCTTATTGCAGGTGTTGGAGGTCTGGAACTTTTCCTCTGTGTGATGGAAGCCATGTGAAGCACAATAAAGCTAATGGAGATAACGTTGGACCTTTGCTTGTGAAGAAGCAATAGCTATAATAGTGTTGTTCTTATGTTGTTTACTTGCTTTCCGTGCACCTTGAACTTTTAACTCATAAACAGAACATTCCTTTAGTGAATGATGTACGGTGATATGAGCTCTCAGGTATGTATTGCTAAACCAA | CV013680 | YES | YES |
| 53 | TUG53 | CTTGTTTCCCCAAGTGTTGTCCCAGCGCTTTCTCATAGTATTGGTGACCCACTACAAGTTTTCCCATCTTGGCGCTTGAATGCCAACTTAAATGCTGAATCCCACATCCTTCCAAGAGATTTGAGAGGTTATTCCTTCCTAGGGAAGCACGCCCTCGCCAGTCGTAAGTATGGTCTGTCTCAGTAAAGTATTTATCTTGGAATGGAGTTGTGTCCACGAAGAAGAATTCCACGATTTCTGCATTGAGAATATAAGATCTGAGGCAAAGCCATCTCCTATCCTTTTTTGTAAGGATGGGATTTATTTGTGCCAAACATCTCCTCTATAGTCATGGTTGCCCAAACTGTGTACCACTGTTTTTGTAAGCTTGGGGCAGTGTAAATGTTGCTAAATGACTCTTCAATGCTGGATCATCTACTCCGGTCAATCCATCTTCGTAAAAGTTATCACCGGTGGAAATGATGAAATCTATGTCCGACTTTTCTCCTGTTTTGCCCATCTGAAGAGCAACTTGAGATTGGTTGTAATTTCCTTTTCTTCCCCAGTCACCAACCACCAAACGCTAAGAGACCCATCAGCTTTTGCAGGGTGTTCGAACCTCTCAAGCTCAGCTATGGAGACACTGAAACACAGCCCTATTAGAATAGCAGTGAAGAGCTTGGGTTTAAGAGAGAGATTCATATTCTTGTTTCCGATCAACATTCAACCATGGCGTKGGATGTGAGAGAGAGAGAGAGAGMGMKWGWGAGAGAGAGAGATGGAAAGCTCTMMCCATTTTCTAAAAGCCCTTATCATTTTCCTTACCATCATCTCTTTGTGAACTCAAGCTCAGCTGCTCGAGTATCCAGAAAAACCAGAACTACTGAGTTCATCAAGACATCTGCAGGCAACAACTTACCCTAGACTTGCATAACCTCACTTTCAAGACATGCTAGTGCTATCCAAACAAGCCCTCGGCTTCTAGCCCACACAGCCCTCTCCCCCACACTCAACAACACCAAATCCACTTCGACCATGATGTCAAGGCTGTCCCAAATCCACGGCTTGAAGCAGAGAGAGATCGCCGCATGCACGACTGTGTTGAGCAGCTAAGTGACTCTGTGGACCAGCTGAGAAACTCAATAGGTGAGATGAAGAGACTGAAGAAGGGATCGAATTTTGATCTGACAATGAACGATATACAAACCTGGGTCAGTGCCGCCTTGTACAAACGATGACACCTGCACAGAGGGGTTTGCCGGCAGGCCATGAATTGGCAAGGTGAAGTACATTGTGAGGAGACGGATCGTGAAGATAGCGCAATTGACTAGCAATGCCTTGGCTTGATCCAATCGTCTTGCTTCTCTGGCACCGGTAGATCCACTCAAGAAACCAAG | CV014055,CV013648 | YES | N0 |
| 54 | TUG54 | GGGGGAAGAAGAAAGGACCACAAAAACAAAGAATCGGCTTTCTTACTGTGATCAGATTCTTCTTCTGCTTCATCAAAATCGCTCTCTCTCTCTCTCTCTCTGTCAGTGATCAGTGATCGATAGCGTCGACTGAGACGCATACACTGAGAGAGGATGGGAAGAGGAAAGTTCAAGGGCAAGCCCACTGGTCGACGCCAGTTCTCCACCCCCGAAGAGATGATTGCTGGTACGTCTGCTCGTCCTCGCACATTTAAACAGGCAGAAGCCAAAGAGGAGGAAGAAGAGAGATCCGAGGGAAGAATCCCGAAGAGGAGTCTGAA | CV013630 | N0 | N0 |
| 55 | TUG55 | GGGGATCCACAGTAGTACACTCTAATCTAATCACAGAGAGAGAGAGAGAGAGAGAGACAGAGAGAGATATTAGGAAATGCCGGCGACTTCGACGATGATCGGAGCCCTTCTGGGATTGGGCACCCAGATGTACTCCAACGCCCTCCGCAAACTCCCTTACATGAGGCATCCGTGGGAACATGTGGTGGGTATGGGATTAGGAGTGGTGTTTGTAAACCAGCTGGTGAAATGGGACGCCARGCTTCAGGAAGACCTCGACAAGATGCTCGACATAAGCTAAAGCCGCCAACGAGCGCCGTTACTTTGATGAAGATGAGTAGAACTTGGGCCGGTGTGGATTGAAGCATCYTAAGACTTGGTGTTCCATTGGTTGTAGACTCTCATTTGCTTAATCTACTGTTCTTTGTTTCTGAAGTTGCTTAGAATCTCGCTGCTCTCTGCTGGCATTTWGTTTCTCATAAGCTCTGTGATTTGTTCATGCTGWRAAWWRRAWWRWMWTCAATAATTGCAAGCTTTGAAAGTATATGCAATGCTAGTTTTCATCCAAAAA | CV013608 | N0 | N0 |
| 56 | TUG56 | GGGGACACAAAGCCAGTGGCCAACACTGTACATCTCTCTCACTACACCAAGCTATGATTTCCATGGATTCTCTCTCTAACTTCATCTCTATACCAGCCTTGGTTCTCTCCATAATCCTCGCTCTCCTTTCTCTCCAAATCCTTAAAAGAAAACTCAATGGAAAGAAGAAGTACCATCCAGTGGCAGGGACCATGTTCAACCAGCTACTCAATTTCAATAGGGTTCATGATTACATGACTGATCTTGCTGCAAAGCACAAGACTTACAGGTTGTTAAGCCCTTTCAGGAATGAGATTTACACTTCAGGATCCTGCAAATGTTGAGTACATTCTCAAAACAAACTTTGAAAGTTATGGCAAGGTATGACTTGAATTTTAGAATTACTTTTGTTGTTGATGTTTGGGTTTGATGCAGTTTTTATTTATATTTATTAAGCAAAAATAAATTAAATAAGTGTTTTTATTTTTATTTTTTTATTATCAACTTTTTAACTTATAATAAGTTACATCAAAATTTATATATATATATATATATATTTTTGTAGCCTAACAATTGAGGTTTGAACTTTTGGATGAATTGTAAATTCAACATCTTA | CV013583 | YES | NO |
| 57 | TUG57 | GGGGATCATATCCATCTCCAAGACTAGAGACAGCCTTGTGAAACTGATGATAGAAATAGTATATGAGACATGGCAACCACCACATCTCTRKSKACCCCTCTTCTCTCCTCCATTCTCAACCCAAGACYTCGRTATGCGCTTAATCCCCTCACCTTTCACCCTCCTCTTTCCTCAAAAAGAATCATCCCCCTTGACAACTCACCTCACGAAATCCCTCTAAAACCCACCCAAATTCGGAGAATCTCAGCTGTATCTGGAGATTCATTGCCTTTAGAAGCAAACCCAGTTGAGAATTCTGAGCAGATAGTTTCCGGTGGTGATAGATACTGGATTCACCATCATTTCTGTTCTTCTGTTCATAGCTTTCATTGGCCTGTCCGTTCTCACTATTGGGATTATCTACATAGCAGTGACAGATTTCTTGCAGAAGAGGGAGAGAGAGAAGTTTGAGAAAGAAGAGGCAGCTAAGAAGAAGAAGAAGAGTGGTAAAAAAGGGGTGGTGAGATCTAGGGCTGGACCCAGAGGGTTTGGACAAAAAATTGAGGGCGATGATGAAGATTTTTAGAATGCACAACAATTGTAAGATTCCCATTGTTATTATTTTATTGTGGGTACAGTCAGGTTGTAGCCATGAAGAAATGAGAGTTATAGTATCTGGCCAACTGTGTAGTCGTGTAGAGAGGAGAGTTTGTTGCACTTCTTAGCTACAAAGGGTGAAAGGTTCTA | CV014352,CV013584 | YES | NO |
| 58 | TUG58 | ACTCCAAAGGCGAGGTTGTCGATTATTCCGAGGATTCACTTCTTCCTCTTCTTTGGTGGCTGAAGTGCTTCTTCCTCCTCATCCTCTTCCTCTTCCTCCTATTCCTCCTCCACAACTTCCTCTTCACCCTCTTCTCCACCGTCGTCGTCGTCATCGTCGTCGTCATCATCGTCATCGTCATCATCGTCGTCGTCTTGGTCATCTCCGTCCTCCTCGTCATCATCTTCCTCTTCACCGTTCTCTTCAGCCCCGCCCGCACCTCCTGGACCTCCTTTCTTTGAGTTGCTTTTGTTGTTGTTTGGGGGGTTGTTGCCGACATCACCATCGTCTTCTGATGACAAATCCTCTTCGCCTTCTCCAAAGCCACCTTCACCCTCATCGTCATCATCATCATCACCGTCATCTTCCTCGGCATCTAATGGCTTGGAATAGGATCGGCCATGATAGTCTCCTTTGATTCCATGGCTGACGCTTCTTAGTGATCCGAGGAAATCCTGAGTTTGACCTGAAAGCTGGTTGGTTTTGACTAGGACAGCCGAGACTAGCATAGCTGCTAAAGCTTCTCCGGCGCACGTTAGCAGGT | DY523307 | YES | YES |
| 59 | TUG59 | GCCAATCCCCTATTCCACGTGGTCCATCTTACTAATATATACTATTAAATTCTAGTAATATCCTTCACCTTACTGTTCAAAAAAAAGCTTCACCTTCATCTTCACCTTCCCCTCCCCACTCTCTCTCTCACCAACTCCTTTGTTTTCTCTCTCTAAAAGTAATTAGATCAGCTGCAGAGTGGTAGTGGTGGTGGTGGTGGTGGTGATAGCGTTATGTCAGTGGCGTGCGGGGTGGAATGGGTGCTGGCGCTGGGTTTCCTCCGGTGGGGGTGGAAGCGTTGCACCTACATCGGGTCCTACGACAGCGCCACCTGGCCTCCTGCCACTGCCGAGGAGTTCGAGGCAGTCCCCCGCATTTGTCGCGTGACCCTCGCCGTCTACGAGGGCGACCTCCGCCACCCCCAATTCCCTCCAGCCGGCGGCTACCGCCTCAACCCGGACTGTGTGATAAAGCGCCTCACCTACGAGCAGACTCACGGCAACGCCCCTCCCTACATCATCT | DY523301 | YES | YES |
| 60 | TUG60 | TCTCTGGGAATCCAAATATGTCCATTACTAATTGGACTAGGCTACCCTTATATGGGATGGATTTTGGGTGGGGAAAGGAAATTTGCATGATACCCGGAGCAATCGGATATGATGGGAAGGCATTCATCCTCCCCGGTCGTAATGAAGATGGTTCTTTTCTTATCCCATTGCGTTTGCAGGTAGCACATATGGATAACTTCATTATTTTCTTTTATGAAGATATCTAATGTCATGTTAATGACCCCCAAACCCCCCCCCTCACAATTATGCGGCGGGGTGTGTGGGCTGCTGTAATGGCTGTGCTGCTTTTTATGGTAATGATGTTGTTGCTATTTCTTTGCTGCTGTTGCTAAGATGCTGTTGCTGCTACTGTCCCCTGCTTTGATCAAGAGGGGTGCCTCCCACAACATGGGATTATCCTCTATTTTTT | DN976121 | YES | N0 |
| 61 | TUG61 | TAGCGTGGTCGCGGCCGAGGTACCCTAAACCTCTTATGGATTTAAGTGGCTGGAATATACGTTGCATGGATTCAGGCAGCATGCACCATTTTGTTGGTGCTGATAGTTCCTGCATAAGTTGGGGTCATGCGCAGTCTGGGGAGCTGGGATATGGACCTTATGGACAGAAATCTTCTGCAGTTCCCAAGAAAGTTGACACTCTTGAGGGCATGCACGTCATCAGTGTTGCATGTGGATCTGCCCATTCCATGGTGGTGGTTGACAGAACGAATGTTGGTGACCGACTTGATCAGCTTGACGTCTATGATGGCAAAGCTGCTGGGGAAGGGAGTGTAGAACCTGAGGCTAAAAGTTCAGTTGCCAAAAAAAATACCAAAAAGGGTGCTGTTAAAACATCTGAAAACTCTACTAAGAGGAAGAAGTCAAAAGATTCATCAGAGTCAGAGGACGAAGAGGAGGATAGTGATGATGATGACGACGACGACGACGACGATGACAGCGAAGGGCAGGTAAATGGGCAGGAAGATCAGAAGAGTCAACGAGGTGGAAAGGCTGGTAGAGGTCGAGGCAAGGCTGGTGGGCGTGGCCCGGGGTCGTCCTTCCAACGAGAAGAAAAGCACACAGAGCACTCAAGCTAAAGGAAAATTGGCCAAGAGGGGAAGACCCAAGAAGTAATGAGAAATTTTGTTCTCTANAAATGNCCTGCCCCC | CV699872 | NO | NO |
| 62 | TUG62 | TTCGAGCGGCCGCCCGGGCAGGTACTGAAGCCCCTAGCCACAAGGGCAGGGGAACCCCCAGCATCAGCCTCTTCTATAACATCCTTAATCAGTTTTTCAGCCTTGTTTATATTTTCCAAAGTTCCTATTAGTTCCACAGGCCTGGTGGAAGAATAAGGATCAGCTTCACCATCCCTTGTGATCTGAATCTTGGCACCTGAATTCGRKYKGCMRMAACCGKRYAGKATSCYCWSCYTTGCGCCCAATGAKAACWCCAACCTTATTATTAGGAACCTCCATTTTCCGTGACATTGTTTGAGTCTCAGAYACAGGTTGCTCTTCCGCAGAAGAAAAATCCCCYTGTTGAGGAACTTCTGCTGAGGGTGGTTCCTGAAGCTCCCYTCCGSATGGCTSTTCCMGAGCACTTTCCAAYTGYGGATTTGCAATGGAGGGTGGCTGRRTGTCCTCTTTCTCRTGAYCATCAGTAGAAGGAAMTTGTTCAYTGTTCASRGTCTCYRAAGCTCCCYCTGTAGRCTGCTGAGTATCCRYTGACTCGRTATTATTATTCTCCWCAGATGGCTTTTCAGSAYCCTCCTCCRCAAGTTCATCYMCCTTCTCTTCTTGKACYYCGKTCTCAGCKACYRMGCYGTTGAGCTTCTCCTCCAAACGAGGTCGTTTCGCTTCGGACTCGTCGGAACCACCACCTTCGACAGCCTCCTCCTCTTTCTCCTCCACCTCCTCCTCAGCTCTCTTGATGGAGTCCAATTTGTTCTCGTTCGCATTTGAGTCAGCCATCAGTGTTTCAGGCTCCAAATCTTCATGCTTGCGCTTGTGATTCGACGGCGCANGACTTGAAGGGATCTGAGTTGAGGCACAAAGCAGCTTTCTGANGCCCAAAGCAGCTGCTTGCTGAGCAGCCAAGCGTATGTGCTCAACGCTCAAGCTTGCTGGATGCCNGGAGTCTATTANCCAGCCTCA | CV699834,CV699754 | YES | N0 |
| 63 | TUG63 | GCGTGGTCGCGGCCGAGGTCTTCACTCCCATCGTGAAAAAGAGAGTGAGTTTCAGATCCACTCACACTTGTTTTTGTGTGTTTTTGCTTTTAAACAAACATGGCTCTCATAGTAGAGAAGACCACCACAGGCCGTGAATACAAGGTAAAGGACATGCACCAGGCCGACTTCGGCCGCCTCGAACTCGAGCTCGCCGAAGTCGAAATGCCGGGTCTCATGTCCTGCCGGGTCGAGTTCGGCCCATCACAACCCTTCAAGGGCGCCCGCATCACCGGTTTGCTCCACATGACGATCCAGACCGCCGCCGCCGCCGCCGCCAGCGACGTTCGGAGAAGAGAGAGAGAAGGAATGTAGACTCCCTCTCTTCGTTAATCACCGATCCTCGCGATCGATCTCGATCTTTCGACCTAGGTCTGCTCCGGCGTTTTTGACTTGACCGACCTTTTTTCTGGTGAGTTTTTTCGATGGCTTCTGAGGACGTGAAGACCGNTGAATCTGCGGTTTCGACGAATCGTGAANCTCGCTGAA | CV699713 | YES | YES |
| 64 | TUG64 | TTCGAGCGGCCGCCCGGGCAGGTACTTGAGGAGGTGCGAGGCCTGCTTGACGCCACCGGTGTCGTTGATGGCAATGACGTCAAGAGGGGAATCCTTCCTGCCATGCCAACACCTCAAGAAGTTCCTTCCAATCCTTCCAAAACCATTTATTGCCACCTTCAGCTTTGCCTCCACCACTCCCCTCCCATTGTTCCTGTTCTCACCAATAGCAGAGGTTTGGAAGGCAACGACAGATAGTAAATCTAAGTGGGAGGCTTTCCGAGCAAAGGGAGCGGAGGCTGAAGNGTTGCGGAGGCCAGCGAATTCGGAGAATCCTCTACCGTTGGCCTGCAGGGGAGATGAATTAACCATGGAGAGAGCAGCTGATGCCATGGGTGCCTTTTTGGTGTCAGCCTACCCCTGACGACAACTAAGATGACCACTGAGTTCACCTCAGTTTTACTTGTTAAGCAGCAGCAGCGGCAGCAGCAGCGGCAACGCAATTACAGTGGATGCAAAAAGAAAGAAGTCACAAGACTGGGAAATGCAGGTGAGGAGAAGGAGGAGAGATGGGAAATTTAGTTGGGGGTTGACCTCGGCCGCGACCACGCTA | CV699671 | YES | YES |
| 65 | TUG65 | ACCTGATGAAGAAGATTTTGATGGGGAAGGTGATGAAGAGGAGAAGGGCAGTGATGGTTCTGAAGAGGAAGATGACGACGACGATGACGAGGAGGGTGATGATGAGGATGGAAATTGAGCTTCTTCCATAGCTCATTCCATATTTTCACTTTCGCTGTTTTAGTATGGCTTGTGTAAAAAGGGGTTTTTGAAGGTCGTTAAAGAACCAAGCGACATGATTTTCCTGCCATGGTAGCTGTGTAGAAAACTCTTCTAGTAGGCCAAACATTTGCTACTGTTGTCAATTACAGGTTATCTGACCTTTTCTTTCTTCATCAAGGCATATGTTATTTTATTTTTTGTTTTTGTGTTTTAATTGGGTATCGATGTGGGATGTCCTCTCTTGGTATGCAGATTGCTAGATGAATGAGAGAGTATTTAGAATTTTAAGCTAGCAATGTCATGAACTCCACTGAGATAATAGTTAAATTATAGTTTTGGTCAAAAAAAAAAAAAAAAAAAAAAAAAAAAAACA | CV067167 | N0 | N0 |
| 66 | TUG66 | AGAAGAGATGGCTAAGAGCATATCGTACGTCACAGGTTCTCAGCTTCTCTCTGCTTAAACTCCGCCCCAATATCGCTGTTGTAGATGTCAGGGACGATGAGAGGAGTTACGATGGGCATATAACTGGTTCACTCCACTATGCGAGCGATACTTTCTCTGACAAATGCCCAATTTAATTCAAGCTGTTAGAGGCAAAGACACCCTTGTCTTTCATTGCGCCCTAAGCCAGGTTCGTGGTCCAAAGTGCGCACGTAGGTTGGCAGATTACCTTGGAGAACTGAAAGAAGACATGGGAATAAAGAACATTATGGTTTTGGTAACGTGGTTATAATGGTTGGGTAAGCCTCTGGTAGACCTGTTTGTCGATGCACCAACATCCCTTGCCAGGGTGACAGCGCGTAGAACATATTGTTATTATTATTATTATTATTAACCCATGTTGGGGACATTTATTGCATTGTATGCAGAATACATGTCTAAACAATGACAATTACTTCATATCACAGAATATGTGATCCGTTGTTGGTCAGCATATGTTGATTACTTGAAGTTACCTTTATCTAAAAA | CV066952 | YES | YES |
| 67 | TUG67 | GGGGTATATCCAAAAACCCAATTTCTAATTGAAGATGGGAACAAAAGGGAGGAGGTCTGCAAGGGCCTCTCTCATCGACCACTGTCCTACTGGCAGTAGTGTCTCTGAGCTTGCCATTAGAAACCACAGCAAACTACAATTACTCATCTCCACCTCCTACCCAAGAAAGTCCCCTCCACCACCTCCTCCTCCTTACTACTACAAATCACCACCACCTCCTCCGCCAGTGCATTACTCACCACCACCACCTCCTCCGGTATTACAAATATAAGTCTCCACCACCGCCACCACCCAAGAAACCC | CV014907 | N0 | N0 |
| 68 | TUG68 | GGGGAAAAGCAAGAATTCTACTGATGATGGAGGTAGTAATGGTGGAAACTCGTCCTGGTGGTGGCGGCGGCGGCGGCAGTGGCAGTGGTGATTTGGGTGAGCCAGAGGATGAAGGCTTTGCTGGGATCGTGGATGAAGTACTGCAAGTGACCTTAGCAACTATGGGTTTGGTGTTCTCTGTATATTTACATCATCGAGGGTGAGGACATAACTCGGCTAGCACAGGACGTCATTAAGTTGGTGTTTAGTGGAAGGAAGAGTGTTCGTCTGAAGCGTCTCTTATATGAATGGGGAATGTTCTTCCTGAGGCAGACAGAGAAGAAGGAGATGCGGGAAGATTGGTTGGAATGTGCAATTATCACTACTCCTACACTGTACGATAGCCCTGAAAAGTACGAGCGCATCCTTCGGTCCTACCTTACTACTACTACGACGACTACAACTACCAGATCTGATGGTGATGATGACGGTGACTAACTGACTCCTGATCCTGGATACTACGACGGAGTAAACTGCTTGT | CV014834 | NO | NO |
| 69 | TUG69 | TTCGAGCGGCCGCCCGGGCAGGTACTGTTGAGATTATTACCCCTGTGGAGCTTATCAAGAAGGGTGACAAAGTGGGGTCTTCTGAGGCTGCCCTCCTTGCAAAACTTGGCATTCGCCCCTTCTCTTATGGTCTTATTGTCTTGTCTGTTTATGACAACGGATCAGTCTTCAGCCCAGAGGTTCTTGATATGACCGAAGATGATCTCATGGAGAAGTTTGCTGCTGGAATTTCCATGGTCACTACATTGTCGGGGGCTGTTTCTTACCCAACTCTTGCAGCTGCACCCCACATGTTCATCAATGCCTACAAAAATGTTTKGGCTGTTGCAGTTGCAACTGAATATTCCTTCCCCCAGGCAGACAAAGTTAAGGAGTACCTCGAGGATCCGACCAAGTTTGCTGTTGCTGTGGCTCCTGTTGCAGCTGCTGCTGATTCTGGCTCTGCTCCTGGCTGCTGCTGTGGAAGAGAAGGAAGAGCCCAGCGGAAGGAGTCTCTGATGATGACAGTGGGATTCTCAGCTCGTGTCTCGRTTAATTTAGCAAGCTTGTTATGGTGTGGCGTTTTCAAACTTATCCGTCTTTGCTTTTTTTTTATTTTTTTATCTTTGTTGAATTGGAGTAATGTAGGATTTGTTGTTTTGAATGGATTGTAACTTCTTGGTCAAGTTTTAATATTTAGACCAGTGATCTTCTTGGTTTTTTCTCTTATCTTGTTCCTTAATTGTTCGTTTGATAAATCTGTGTGCCATTCATGTCTTGNATTTATGGATAAGCCCTGGCTGTGAAGCTCTATTATATTTCTGTGATCTTTACACAAAAAACAAAAAAAAAAAAAAC | CV699581,CV014822,CV014112 | YES | NO |
| 70 | TUG70 | AGCAGGTGGTATCAACGCAGAGTTGCCAGTTACGGCTACAGCGGTGACTGAAGCAAAACAGAGAGACTCTTCTGTCTTGCCAGAGAAGATGAGCTTCAAGAAGAGAAAGGCTGAAAAGAACCACAACCTCAAGGCAGTAGCAGAAGAAGAAGCTTGTAAGGAAAAAAGGATCAGACGATGTACCGAAGAGGGAGAGTCGAAGATCACAGAGCAGAACAGCGACAACGACAGCAAGAACACCAGCAACAACAATAACAACAAGAGAGAAACATCTGCAGATACTTCCAAGGAGAACTCGAAGGTTTCGGAGGTTCAAAAACCTGATTACATTCACGTTCGTGCACGGCGTGGCCAAGCCACTGACAGCCATAGCTTGGCCGAAAGAGTGAGAAGAGAGAAAATCAGTGAGAGAATGAAATATCTGCAAGATTTAGTACCGGGATGTAATAAGATCACGGGAAAGGCTGGAATGCTTGATGAAATCATCAACTATGTTCAATCTCTTCAAAGACAAGTAGAGTTCCTGTCCATGAAACTGGCTGCTGTAAATCCAACACTCGACTTCAACATCGACAATTTTGTAGCGAAACGAGGTGTTTCAGCATGCACATCCAATTTTTCCAAATCGGGGTATCACTCAGAGAATCGGCGT | CV014765 | YES | YES |
| 71 | TUG71 | GGGGCTCTTCCAAATGGAAAAGAAACTTGTATTTTAGAAGCAGAAAACTCCCAAATAAATGGCTGTGTCATCTTCCATGGCGGCGGCAGCAGCAGCAAGTGTCGTTTACATCTCCAGACGGCAGAACGTTGCATCATCATGGGCTTCAAGAAGAAAAAGAAGAAGAAGAAGTTGTTGTGGCCCGTTCTTCTCCCAATCCAGAATCATTACTACTGCCTTCCCAACAAAACCCCCCAAATACGTAATACGATGCACCGCCACTGGTAATCCATCTCCAGAAACCGAGACTCCTGTTGAAGTTCCCAAAGGACCTCCTTCTCTCATTTCTGCTCTTAATGTCGAAAGAGCTCTTCGCGGGATCCCAATTACAGATGTAGATCACTATGGCAGACTTGGAATCCAAAAGTCCTGTCCATTTGATCAGGTTTATGTGGCATATAAGAACAAGGTAGAAGCTTTGATGAACGAAGGAGAAGACGAAGAAGCACTCAACAAGAAGCTTGAGCTTTTAAAGGAATCGTACTCGGTTTGGTCGAATGCACAAGAGAGAAGACTGTACGATGTGGAGCTTGGCTAGGAGTGAGAAGCCAGACAACATACATATGGCCTTATCGAGGTCGACACCACAAAAAATCAACCGAGTCTCCTCCTCCAAAGGAACCCAGAAGACGAAAGGGCAACAACCTTAGG | CV014723 | YES | YES |
| 72 | TUG72 | GGGGACACAGTCCCGATCAATCCGGTGTCGGGGATCGACAGAGAAGCGAGGGTGTTGAGGTACAGAGAGAAGCGAAAGAACAGAAAGTTCGAGAAGACGATACGGTACGCGTCGAGGAAGGCCTACGCAGAGACCAGACCKSGAATCAAAGGAAGGTTCGCGAAGCGCGCCGAGATCCAGCTCGATAGCATCTACAACAACTCTTCCTCCCCKGCCGCCTTCATCGCCGCCGATGCTGGRTACGGCGTCGTYCCTTCTTTCTAAGTAACTTCAAATCTCTTTCTTCTTTAGCGTTTKGTAATTCTGTTTTCATATCAACGATCTGGTATATATAAATGACGATGACGATGATGATGATGATGATGATCGATGGAGTAGTKGTTATKGCTGCCCGTCGATTTKGTAGAAGAAATKGTTGGTATTAATTTTGGTAAACTGGTTTTTCCAAATKKYCAWMRWMRWWWCWWSAMAWWRWWRAARWWSWWRYAMATGCGATGGATTTGGATAGTGTTGAGCTCAGCTCAGTGTACT | CV013747,CV014663 | YES | YES |
| 73 | TUG73 | GGGGCCAAAAAATCACTTTTTACCATCTTCCCAAAATAAGTCAGCAGAAGGGGCTCTATGGGTCAAGACGCCCACTACAGTATTTTTTTTCTCAAAAAAAAAAATAATAATAATAATAATAGTAATAATAATAATAATTTAAAATGGATTATGGTTTATTTCTGGGCTAAAAATGGTGATTTTTTGGTCTTGGCAGGTTACACAGTCCCATAAGAAACCAAGCTCAGAGATTCATAGCAAAATCCTAGGGTCATGAGTGGTCCTTATATTTTTATTGGCTAAATAAGAGTTGCTTGGACAAAGCAATAATAATAATATTTTAGAAAAAGAGTTATAAATATTGGCATGTAACTTTCAGTTGAATTATGTGATATAGAATAGCATTTCCTATGCTGGCCCCTACAACTCTCTTTTTAAAGAAGTACGTTCATACATTTACAACTTTTAAGTTATATTTAAATTTGGAATTGGTTCAACAAAAAAAAATAAAATTTGGATGATGGGTGGTGTAGTTTCATACCTTTTCTTTCGACACATTCAACATCAATGAGGGACACATATAACTAAATCATCTACAAAAAA | CV014636 | YES | YES |
| 74 | TUG74 | GGGGACAAACAAATCRCAAAACMTTTCAAATCACCCCCTTCCTATTCAAATCACCACCGTTGATTCTACTTTTTTCCGATCAGCACCACAAKGGCCGAAGAGAAGCACCACCACCACCACTTCCACCACCACAAGGACGACGAAGACAAGCCCATCGATTCAGCTCCTTATTCCGGCACCACCACCGGATATGGTGGCACCACCACCGGATATGGTGACACCACCACCGGATATGGTGGCACCACCACCGGMTACGGTGGCGTTGAAGAAGTGACCACCTATGGTGAGACCACCAACGCCTATGGAAGCGGTGGTSGAGSGAGYSAGYMYRGKSAGAGTACTGGTGTTTATGGAACTACAGCACCTCCTCGTTTTTCTGAGGAAACCCCTGATTATGAGAAAGAGGTTAAGCAGCACAAGCACCGTGAGCACCTTGGTGAGCTTGGTGCTGTTGCTGCTGGCASTTTTGCCTTGTATGAGAAGCATCAGTCAAAGAAAGACCCAGAGCATGCCCACAAGCATAAGATAGAGGAAGAGATAGCAGCTGTGGCTGCAGTKGGATCTGGTGGGTATGCATTCCATGAGCATCATGAGAAGAAAGATGCCAAGGAAGAAGATGAAGAGTCCCATGGCAAGAAGCACCACCACCTCTTTTAACTTTTGAGTGGCTTGCTCTGTACYWYGKCYKCGACYWYGCTATTCATCTTTAATTTGTGGTTTGTCTTTAATTTTATTAAGTGATCAAACTACCCTAATAATTATAGTTTGTGTTGAGTCAGTTTGTGGATTAGATTACTGGGATCTTATGGTTTCTATGCCATGACCTGGTATGAGGGTTGCAGGTGTTATCTTGGCCCTGTTATCATGTGATGTTTATAATAATTGTGTTTGTGAGTTGCCAAG | CV014588,CV013741,CV699610,  CV699597,CV014349,CV699541,  CV014525 | YES | YES |
| 75 | TUG75 | GGGGTGATCCGATGGTGAATTCGATGATGACGATGACGATGACGATGATGTCAGCCCTAAGAAGATCGGGTATGTTACGATTCCTGCTGTTGATGATCCTGTCAAAGCTCCTCTTGTGACCAAGGAGGTGGTCTGAGAAAAAAGGGTGGTATTGATTTGATGTTTCGGATTTAAGGGAATTTGTTCTTGTAAGTGTGGGCTATTAAACAAGTGCTACTTTATCTTCATCTGTTAACCTCTCTCTCTCCCTCTCTACATATATCAACACGTGCTACATATTGGTGATCCAATAAGCCAAAATGCTACTCTTTCAAAAAAAAAAAAAAAAAAAAAAAAAAACA | CV014586 | YES | YES |
| 76 | TUG76 | GGGGACAAATCCAATCGATCACATTCAGATCCCCAATTTCCCATCCATTTTTCTAGCGCGAAAAAAAGAGAGAAAAKGGCAGGAATCATACACAAGATAGGAGAGACCCTCCACATCGGAGGCCAAAAGGAAGGCGAGAAGCAACACAAGGAAGGTGAGCACAAGGGAGAGCACCAKGGCGATATGCACAAATCGGAACACTATGAAGGAGAAAAGCACAAATCTGAAGGAGAGAAGCACAAATCGGAGCACCAGTGGAGATGAGCACAAGGAAGGAGGGTTCATGRGCAAGATCCAKGGTGGTGGTGATCATCATGGTCATGAGGAGAARGGTGAGAAAAAGAAGAAGAAGGAGAAGAAGAAGAAGCACGAGGATGGACACGAGCACCAGTGGTCATGACAGCAGCGACAGTGATAGCGATTAGATCGCTCGCTCGCTTTATACTTCTTCCCCTACACTACTTCGAAGGTGGGCTTGGCTCTGAGGAGAGCTACAAATAAGAGGCCGATAGAGATGGGTGTGGTGGGAGGGAGAGAATAAAATCTTGCTTAGGTGTTATGAGATGTTATCAGGTGGGTTGAAGTGTAAGGTCTACAGATGATGTGACATACTATAACATTAC | CV014929,CV014545 | YES | YES |
| 77 | TUG77 | GGGGGGGTGGGTTAGACTTGAGAAGGAAGGAAGGATCCAATCCGATCCAATCCATAAAAAATGTCTTCGGCGGCGGCAATCTTTATTTCAACTCCAAACAACCACCACCACTACCCTTCTTCTCAGTTCCAGAAGCTCTTCCGTCATCGGCCAGATCTGCGTCGGATTTCGATACCCACTTCCACATGGGACCCACAGCAACAACAACAACAACAACAAGAGGACTTCATCTTCTTCTTCTTCAATCTTCAAACCCTTTTCCCCTGTCATGGAATGGCAGGATTGCACGATTAAAGATGGATATAGACGTCCCTGCTTCAGTTGCCTATAATTGGTTATTCTGATCGTGAAGCCATTCCCACGCTGGATGCCCTTCATTTCATCTGTAAAGATTTKRGAAGATAAGCCTGACCTATCRCRAGTGGTCRYTRAAGTATRAAGCATTTGGCCGTGATATTGAATTCTCTTGGCTTGCTCGAAATATGCAGCCCATCCCWAATCAGAARATCCAGTGGAGATCTCTTGAWGGTCTCCCYAAACAGCTARSWGTTKCAYGATKWWGTMCCKCAAMKCTGGGYTYCATGGCATGCMGTACTAAATCCTTTTACTGAGAGCTTACTTTTACGAGGTTTAGAAAGATTTGCAAAGTTGCAAAAAGCTACCCCACTAACTCAAAAAGGTGATGTCCAAATTAAGGGACTTAGGTGATGACTTTACCAAAATTATTAGCATGGACCATTCTTTCAAAGGATGAAGTAAGTCTGGATACTAAAGATGTAATGCATCTGTATTTCATAAAGAGGAATTAAGATATGTCCATTTATTTATCCATTTAATTTATAGAGACATTTCTGTCTCTTTAGACAAAAAAA | CV014458,CV014464 | YES | YES |
| 78 | TUG78 | GGGGGCAGAAGACCTCCGGACTGTCATGAAAATTGATCCTGGTAACAGGGTTGCAAGAAGTACCGTTCACCGCTTGACTAAAATGGCCGGCTAGAGAAGTTTCTTCTTCTTCTTCTTCTTCTTCTTCTTACTCTTTTTTAGTTTTTTCCTTTTGCCCATACGGTTGATAGTTTTATTCACCCTCTTCACATCAATGTGTTGGGGAATAATAACATTCTGCTTGGAAACATGTTTATTCTTTATATGTTTGACCTGTAATTTTACATGTATGATTTGGTTGTATTTCATGTCTTTGCTGGTTGTGAGTTGACTAAAAAAAAAAAAAAAAAAAAAAAAAAAAAA | CV014435,CV014452 | YES | YES |
| 79 | TUG79 | GGGGGTAATTTAAGGGTGTCCTAAAGGTGTGTCACCCCATTCTCTCTCCCAATCCTCACCGCCACCACCACCACCACAACCACCATCATGTCGGAAAAGGAGTGCAGCCTCCACAAGCACAAAAAACACAAACTCCTCCGCCGCATATTCGCCGGAATCCTTATCACCCTCTTCATAATCCTCATAATCGTCCTGATAATCTGGGCCATACTCCAACCCAAAAAACCCCAATTCAGCCTCCAAGACGCCACTGTCTACTCGTTCAACATCACCGCCCCAAACTTCCTCACCTCCGCAATCCAAATTACAATCTCCTCCCGCAACCCCAAACGATCAAATCGGCGTCTACTACGACAAACTTGACGTCTACGCCACGTACCAGAACCAGCAAATCACATACTACACCTCCATTCCTCCGACCTATCAGGGCCCACATGAGCGTCAACGTCTGGTCCCCCTTCGTCTCCGGACACCACCGTCCCCGTGGCTCCGTACAACGGCGTCGCTCTCAGCCAGGACGAGGCCGATGGAACAGTCATCTCTTGTTCAAAATCGACGGTCGAGTTCGATCCAAAGTCGGAACGTTCATCTCCGCTCAGTATCCTCTCTTCGTTAATGTCCGCGGTCATCACGT | CV014413 | YES | YES |
| 80 | TUG80 | GGGCAGTCCAAAAGTCATCAATTTGAAGGTGCTCTCATCCATGGCTCTCCAGTCTGCTGCTGCTGCTGCTGCTACTCTGCTTCCTTCTGCATTCTCCATCCCCAAGAGAGCAATTCAAGTGCATATTTGAAGAATTCCACTCTCTTGGGAATGTCACTCTCTGATCATGTCAAAGCTGATTCCAGCTCTTGTTCATTGAGAATCAAGAGGGACTGCAACCAAAGAAACCTTGCAATTCAAGCAGTGGCTACCACTCCGC | CV014376 | YES | NO |
| 81 | TUG81 | TTCGAGCGGCCGCCCGGGCAGGTACTGTTGAGATTATTACCCCTGTGGAGCTTATCAAGAAGGGTGACAAAGTGGGGTCTTCTGAGGCTGCCCTCCTTGCAAAACTTGGCATTCGCCCCTTCTCTTATGGTCTTATTGTCTTGTCTGTTTATGACAACGGATCAGTCTTCAGCCCAGAGGTTCTTGATATGACCGAAGATGATCTCATGGAGAAGTTTGCTGCTGGAATTTCCATGGTCACTACATTGTCGGGGGCTGTTTCTTACCCAACTCTTGCAGCTGCACCCCACATGTTCATCAATGCCTACAAAAATGTTTKGGCTGTTGCAGTTGCAACTGAATATTCCTTCCCCCAGGCAGACAAAGTTAAGGAGTACCTCGAGGATCCGACCAAGTTTGCTGTTGCTGTGGCTCCTGTTGCAGCTGCTGCTGATTCTGGCTCTGCTCCTGGCTGCTGCTGTGGAAGAGAAGGAAGAGCCCAGCGGAAGGAGTCTCTGATGATGACAGTGGGATTCTCAGCTCGTGTCTCGRTTAATTTAGCAAGCTTGTTATGGTGTGGCGTTTTCAAACTTATCCGTCTTTGCTTTTTTTTTATTTTTTTATCTTTGTTGAATTGGAGTAATGTAGGATTTGTTGTTTTGAATGGATTGTAACTTCTTGGTCAAGTTTTAATATTTAGACCAGTGATCTTCTTGGTTTTTTCTCTTATCTTGTTCCTTAATTGTTCGTTTGATAAATCTGTGTGCCATTCATGTCTTGNATTTATGGATAAGCCCTGGCTGTGAAGCTCTATTATATTTCTGTGATCTTTACACAAAAAACAAAAAAAAAAAAAAC | CV699581,CV014822,CV014112 | YES | NO |
| 82 | TUG82 | GGGGAAAGAACAAAGTTAGAGAGAGAGAAGTGGCCATGGCTACATGTAACATGGCATCAGCTGCAGCTGGGTTTGTGCTCTCATCACCATCATCATCATCATCAAGTAATGCTTTGACATCAAGCTCTTCTTCTAGGACTGGTGTGGCATTTCCTCTTAAGAACAACAACAACAACAATGGCAGTGTTTCAAGAAGGATGATGGTTGTGCGTGCATCCKASKMGSCSKWKCYACCAGCTGCCWYCAWRACCGCCACYACCWCCGKCTCACCTYCTRRGSYACCTCCRATWGKASYCAAGAGWSSYACCGTARGGTGAGAATTTCCTKAAAAGGGATAGGAGTCCTTTACCTGGGTTCAAAAGGCCATAGGTATCCTGTTTGTAGCSAGTCGATCTAGGTACCCAATAGTACTCGTATATCCAGTGGTGGTTCGATTCAACAAGGTCAACTATGCCAATGTATCTAACTTAACAACTATGCACTAGTATGAGATTGAAGTAAGWCGCAAATGAATTCTATATATATGTGTGCCTATTTTGAGTGTGGTTTGAATTTGTGTAATACTTCTCTGTATTCTATCCACTCTTGTTTCTGATAATTTTATCACTATGTGSTAGAGACCAATTTGAATTAGCAAAAAAAA | CV014045,  CV067080 | YES | YES |
| 83 | TUG83 | GGGGGAGAGAGTGACATAAACCCTAGCCGCTCCAATCGATTKKSKCTCTGTTTCTCTGTTAGTCTTTTGCAGGTTGTTGGAGTACGCCAGAATAGTTGACTGTTAAAAATGGCTTTCTCCAATAGATTTGGAAGTCTCATGAGGCAGACCATTTCACAGAATAGCATATCGAATGGGCACGTTTCAATGGCATCTATGCTTAATGCCATTCGTTGCATGTCATCTTCGAAACTTTTCATTGGAGGCCTTTCTTTGGAACTGATGACCAGTCTCTCAGGGAGGCATTTTCTGGCTTTGGTGATGTTGTAGATGCAAAGGTAATTACTGATAGAGATACTGGGAAGTCTAGAGGATTTGGGTTTGTGAACTTCTCGAGTGATGAATCTGCCGGCTCTGCGATGTCAGCAATGGATGGACAGCCACTGAATGGGCGGAACATCCGTGTGAGCTATGCTAATGATAGACCATCTGCTCCTAGAAGTAACTACGGTGGTGGTGGTGGTTATGGTGGTGGTGGTTACGGTTCCCGTGGCGGGTATGGTGATGCCAGAGAGAATGATGGTTTACTAGGCCACATGTGGATAGTTCTACTATTGAGAGCATGGCCTATGTTAAGTTCACTTATCTAGGAGGATATCATGGGATGCCTTATTATGTTTTAAATGGTTTCT | CV014001,CV013980 | YES | YES |
| 84 | TUG84 | GGGGGCTCTATGAACAAAGTTTCACAATTTTCTCTCCGAAGCTATGTCGAAATTTTCTCTCTCTTTCTTTTCTCGGCCTTTTTCTTTCCTCTTCCTCCTCCTGAGTTTCAGAAGTGGATTTAGAGAGCGTAGGGAGATGAAGAGCTGTGAGCTATGCAAGTACGCGGCGAGGATGTACTGCGAGTCAGACCAGGCGAGCTTATGCTGGGACTGCGATGCCAAGGTTCACTCCGCCAATTTCCTCGTCGCTAGGCATTCGAGGAGTTTGCTGTGTCACGTTTGTCAGTCTCCGACGCCGTGGAATGCTTCTGGTGCGAGAGTCGGCCGGACTGTTTCGGTGTGTGAGAGTTGTTTCGAAGTGAAGGACAACAGAGTCGATGAGGAAGAGAACGAAGGTGATAACTATAATGAAATTGATACTGAAGAGGATGAGTATGGAGATGATGAGAAGGAGGACGATGATGATGAGGAAGGAGATAATCAGGTGGTGCCGTGGTCATCGACTCCTCCTCCTCCTCCGGCTGAGGGTTCTTCAAGCAGTGAGGAGTCCTCGAACAGGTCCATAAATGGCGATGGAGTTGTTTCACTAAAGCAAATTGGCACGAAAAATGCCGAGATCCTTCACTCTGACGAATGAATCTCAA | CV013826 | YES | YES |
| 85 | TUG85 | GGGGGTCTCGACGGAAAATCGAAGGCGGGGTGATGGGCGGCGAGAGGAGTTCCAAGGAGGAGGAGGAGAGGAAGAAGAAGAAGAAGAGAAGCTCCGTTTACGACTCTGGAGATGATAGGAAAAGAAAAAAAACGTAGAAGTTAGAAGAAGAGGAAGCCAAGAGCAGTAAGACCAAAAAGAAAGACAAATCCAAGAGGAGCAAGAATTCTCATAGGTCTTCCAAACACCGTACTGATAAAGCTCCAGAAAAGAAATCAAAAGAGAAGCAGAAAAGTAAACGAAGCAAGCATGATGATCAGTCGAAACTAAATTTCCAAGTGCTGTCTATGGATGACTACTTCTCTAAGAACAATGAATTTGCAACATGGCTGAAGGAAGAGAAAAACATGTTTTTCTCAGATCTTTCGTCAGAGTCTGCACATGATCTGTTTTCACATTTGTCAAGGACTGGAACAGCCATGAGCTTGAATCCCGATACTACAAGGGCATTGAGACTGCGCCTCGGTCCGCCCATAAGTGGAAAATTAAGCAATAGTAGAATCTCTGAACTGTACTGTTTGTTATGCTTTGTTTTCAAATATCTACGTTGTTTGTGAGGGTTGTATTAGTTTCATAAGTTTACTGTGAAACAC | CV013714 | YES | YES |
| 86 | TUG86 | GGGGGMKTCAGTTCSTTSYCMKTTRMCCTWGCACACARAGGCGACGCCTMKCTAYACTATCAGATTTGCAAACTAGTTTTSAATTATCTCYTCCGCGAAAGATGKCTCTGAGCGAWCTCGCTTGCASCTACGCTGCTCTCATCCTCCACGACGATGGCATCCCCGTCACYKCAGAGAAGATTGCGACAYTGGTTAAGGCTGCSAACGTGCCTATAGAATCSTACTGGCCAGGCCTCTTTGCCAAGCTTTTTGAGAAGAGGAACATYGATGACCTCATCATGAACGTTGGATCTGGTGGAGGTGGTGCCGCCGYSGCCTTTTCCGCYCCCGCTGCTGGCGGTGGTKCCGCTGCCGCCCCYGCYGCCGCWGCTGCTGTTGAGGAGAAGAAGGAAGAACCRAAGGAAGAGAGYGATGASGATATGGGAYTCTCTTTGTTTGATTAGGAGCTASGTTCAGTGTGTTTTGGTATTCCTCTAGATGATTAAGAACTATTGAGATTTAAGTTTTTTCTTTTCTGCAATTATGATAGTTGTCTGAAGATTAAAAAATTTCGAAACATCTGATAAAGTCAAAAAAAAAAAAAAAAAAAAAAAAAAAAAA | CV013702,CV013590 | YES | NO |
| 87 | TUG87 | GGGGGAGTCATAAAAAATTAAAGTGAAATTTAAGCGAATTCACATTTTCAGAGGAAGAGGCGAAGAAGAAGAAGAAGAAGATGGGAAGTGAGCTTCGGAACCCTAAAAAGCAGCCACAACTAAACCCTAACTGGGCTCAGCTCTGCAAAAAAAAAAAAAAAAAAAAAAAAAAAAAA | CV013557 | YES | YES |
| 88 | TUG88 | GGGGACCTCATCACCTTAAAGTCTTGCTTTATTTCTTAGCTCAATATCTATTCCAATGGTGGTGATTATTATGGTCTCTACGGGCAACAACCACCACCTTGATTACTCCAAGTTGATTGCCAATGAAGAAGGGGTCTTGACTAGCTCTCCGTGTTTATATGAGACGTGAGGAGCGATGGCRATGACRATGATGACGATGATGATGTTGGTGTGGCAGCCAGCCGCATARCTTTTTTCAGTTTTGATKGTCTAAGGTTTKGAATATGTTAATGGTSAGCTAAGCAAGATASATGAGCTCATAATATTCAGTACTTGRCATTATAAATAACCTGTCTTGCTATTCATATTAATGTTCTAGTATATGATAATCACCTTCTCTCTCAGCAAAAAAAAAAAAAAAAAAACCAAAAAAAAAA | CV014474,BQ925884 | YES | NO |
| 89 | TUG89 | CCTCGTAATATATGCAAGACCTCCTTCAGCGAATGAGTTTTTCCTCCATAGCTGGTGTGGTGAGTCGATCGAAACTCATATCAAGGGCTGCTCTCTCTCTCTCTCTCTAAAAACTACTAACCCAAGATGGAAATGTGAGAATGTTATTGTCACTTTTGTCTTTTTTTTTTTCCTGCGGATTTCTTTTGGTATTTTAGATTTTGTTGATTGCTCTGTATGTATGTATGTATGTATGCATGCTTGTGTTTTGTTCAAAAAAAAAAA | DN976132 | NO | NO |
| 90 | TUG90 | CCTCGTCCCCCACGATTTACAATTTGAAAACAAAAGTAGGATTTTAATGTTAGGAACGGAAGAAACATAGCTTAAAATTATATGAGGGGAAGTGTGAAAAATCAGTTTAGAATAGGATGACAAACTTATTGATGAACAATTTTTTTGTTTGTTTGTTTGTTTGTGTGGAATTGCATAGAAAAGGAATCCCAATCTGTATTTTACCAAAAAAAAAAA | DN976128 | YES | YES |
| 91 | TUG91 | TATTTATATGTGGTGTATTCGAATTTCAATGTTGTTTGTTTGTTTGTTTGTGTTTATGAATTGTTTAGTTTGTAGAATATATATATGTGACAACTTGGCAGTACTGATTCTATTGTACGAAAATCATGTTGTATCTTTATTTTCAGATTGTATTCATGTTGTATTGTAGTTGTTGTTCCAAAAAAAAAAA | DN976105 | NO | NO |
| 92 | TUG92 | CCGGGGAGAGAGAAGATCTAGGGTTTTGAGCAAGAGCAAGGATGTTTCATCAATGTTCGCGAGGAAACCATGACAAAGAGGTGGCTCTGAAGCAGCTGAGGACACACGTTGCCATGTTCGGAGCTTGGGTCGCCGTGGTTCGGATCACCCCTTACATCCTGATACTACTTCTCGGACCACAAAGAAGAACTCAAGCTCGACTTCTAATCCTCTATCAGTTGGCTTGGTTGCCGGGTAGTGAAGGAATGCTGGGACTGGGAGATGATCATTAGCTTAATATATCTTCCTCTCTCTTTTTTTTTCTTTCTTTTTTTCTTTTTTTCCTTTTCTTTTAAATCTACTCTAAATCTCATGCCAGTGAAAGATTGAATTCTGTTTCATCTTAAGCTTGCCTTTTCCAAATCATTTTTATTTTCATGTTGGTACGGGACACAATGTACTCATTTTTTATTATGTTTGGTACAAAGACTGAACATGTGGTTAATCAGTAGTCCTACTTTCAAAGCATGACATTCAAATTCAGGCTCTGTATGCCTCAGAACATTGTAAGGTGCTATCATTATTGACATTTATGAAGCTAGAAACCCACAATCTTACCACCGAAAAAAAAAAAAAAAAAAAAAAAAAAAAA | CV067041 | YES | YES |
| 93 | TUG93 | GGGGGGGACTTGGAATCAGCTTCGTCAGTATGTGCAAAATCGTAGCCGCAAGGTGTGAATAGATTCTTGACAAGGTGTGCTAGACAATCTGTTGCTACCAAATTTTTTAATTTTTTAATTTTTTACTGTATGTATGTATGTATGTATGTATGCACTGATGCCAATGGTGGGTGACGGATGTTTAGGGATGTTTAACTTATGATAATGGAAGTATCTCCTTTCTTCTCCAAAAAGATAAAAACATATAAAAAGAAAAAAAAAAA | CV014589 | YES | NO |
| 94 | TUG94 | GGGGGCGCTCTGCAGTTGGGTTAGTTGGTTGGTTGGTTGGTTGGGGTTTCGTTTTCACTCTTTGGGCAGAGCATAGCATTGAGTCCCAAATGGCTTCTTCTGTTTTCTCTTTTTCTCTCTTCTTCTTCTTCCTTCTCTTCTCCTTTACCACCTCTCTCCACCTCTGCACTAGCCTTCTCCGCCCAACACAGCCCTCTCCGCCACCACCTTCGCCACCCTCGCTTCGCCGGACACAACTACAGGGACGCTCTCTCCAAATCCATCCTATACTTCGAAGGCCAGAGGTCTGGAAAGCTCCCTCCTAACCAGAG | CV014500 | NO | NO |
| 95 | TUG95 | GGGGCTCCTTCCTCTTCTGATCTTTGATCCCTCTCTTCTCAAACCCTAACCTATCCCTAAAAACAAAACAAACAAACAAACAAAAAAACCCAAACACCAATGGCAACAACCATAGCAACAACAATGGCCATGCTCAATCCCAACTTCACCACTACCACCAACCCTCCTAAGCTACCACCACCAACAACAAAACCCACCACCCTCCTCTCAATGCTAACCCTCCCAAAAGCCCTAACCACCTCAAAACCACCACCACACAATCCTAATCTCTCAACCGCCCTCGCCGGAGCCGTCTTCTCCACCTTGAGCTCATGCAACCCTGCCATGGCAGCCCAACAAGTAGCCGAGATAGCGGAAGGCGACAACCGCGGCCTCGCCCTCCTCCTGCCCATCATTCCAGCCATTGCTTGGGTTCTTTTCAACATCCTCCAACCAGCTCTCAATCAAATAAACAAAATGAGTAGTAGCAAAGGAGTGATCATAGGGCTAGGACTAGGTGGTAGATTATTAGGAACGTCAATGACTATGACTCCTCATGCTTCAGCTACTGAGGTTGCTATGGTGGCTGAGGCTGCTGCTAGTGACAACAGAGGGCAGCTTCTGTGTTTGTCATCGCTCCGGCGATTCTTGGGGTTCTTTACATATTCTACAGCC | CV013811 | YES | YES |
| 96 | TUG96 | GGGGAAGGACGATGAAGTAATCGCCGAGTTCTGCAGCAATGTGGAGTCGGCAGAGGCGTTTTCAAGGTTGGATGCATAACAATAAAAGCACTTCTTCTGGAAAAAAATAGGATTCTAACAATCAAAACAGCAATGGAATTAACACCATTAACAACCACCCACTCACCACCCACCCACTGACGACTGTAACACTTACTGGTGGTGGAACACTGCTGTTGGTACCTAACTGGGTCGTCTTCTTCTTCTTGACTCTTTGACTGACTGACTGACTGACTGGACGACACACCTACGTACGGAACTGACGGCGACGAGACGACGACGACGACGACGTAGGTGGTCTTAGTTTGGC | CV013685 | YES | NO |
| 97 | TUG97 | TTCGAGCGGCCGCCCGGGCAGGTACTCCCTTCCCTTCCCTKCCCYTCCCYTCCMTGGCAACTATGGCTACAACCCTCACTTCCTCTCTTTCCTCCAACCCCAAGACTTCCTTCTTGGACACCCACCAATCCTCCTTCCATGGCGTCCCTCTTTCTTCCCCAATTCGCCTCCAACCCATCAAATCCACCCCACACAACCTTTCTATTTCCATGTCCGCTTCTTCTCCTYCCTACGATCTCCGATCATTCACATTCGAACCCATCAAAGAATCGATCGTTTCGCGCGAAATGACTCGACGGTACCTCGGCCGCGACCACGCTA | CV699734,CV699773,CV699679,  CV699772 | NO | NO |
| 98 | TUG98 | GGGGCTCTTCGGGTGTGCTAAAGTTACTACCCCAATCAAACGATACGCCTGTTAGCTAACCTAAGCCATGAGCTTACTAGATCAGCTCTGGGACGACACCGTTGCTGGTCCTCGTCCCGAAAACGGCCTCGGAAAGCTCCGTAAACAACACACCTTTCACTGTGCGTACCCGAGCAAGGAATCGGAGGGTTGGAGGTCCGGAGGAGGGTTATGAGGGGTAACGAGAAGCATCATGGATACCTGAAACCACCGCCGKACTCAGAACGGTTCGCCTCCGGTTTCGCCTGCTGGATCTACGCCACCGGTATCTCCGTTTGCCGGAGGGAGCAGAGAGGCATACCGGTTCCGGAGGAGGTCTATATCGGATGTGTACGAGAAAGCAAATGGGATTGGACCCAGGAGCCCAACTCCTCCTGACGACATGTGAGATTTGAGTTCACTTTTTTCTCATCAACCAGCACAAGAACAAAAAATTGARAAAATGAAATAAGAACAAGAAAAGAAAAGTTCTCCTCTCTTATCTTTTTAGTGGGTTGTTGTGATTGAAGCTGAAAATGTGTTGTGTTGTGTTGTGTTTGTGTAGAAGTGTGTGGTTGYGCRTGYGSCTTCTTGTAGTATATGTAATATGGCTTGTGTTATCGCGGGAGGAGAGTCCGAATGAGGCTGCTCAAAGCGTTGTTAGCTGGTAGTGTTTGTAATGGATCTTTCCTCTATATAGAGCTGAGGTTTTCTCMAAAAAAAAAAAAAAAAAAAAAA | CV013580,CV067110,CV066947 | YES | YES |
| 99 | TUG99 | GGGGAGAGTGTGAGAGAGAGGCAGRGRGAGRGRGACAGAGAATAGGGTTTKGCAGAGGCCAGCGGAGAGGAGAAGAGAGGAGAAGAGAAGAGAAGAGATGCCTTGCTTGAACCTGTCGACTAACGTGAACGTGGAGGGCGTTGACACCTCCTCCATCCTCAAGGAGGCCACTTCCACCGTCGCCGATCTCATCGGCAAACCTGAGGCTTATGTGATGATTGTGTTGAAGGGTTCCGTGCCCATTGCTTTTGGTGGGACTGAGGAGCCAGCAGCCTATGGTGAGTTGGTGTCCATTGGGGGCTTGAACCCAAGTGTAAACAAGAAGCTTAGTGCTGCAATTTCAGCGATACTTGAAGCCAAGTTGTCTATTCCCCCGTCACGATTCTTCCTGAAATTCTATGACACTAAGGGCTCCAACTTKGGATGGAATGGAACCACCTTCTAAATTCAGTGCCACTTCTGTWGTGTTATTTTGAGTCTATCGTGAACTGGATGCTATGTATGGAAAGTAAGGCTATTKGTCTTGTAACTTGGTATTATAATTTATAACTTCTATGGTAGGTACCTATCTAAAAAAAAAATACTTCTATGGTAGTATKGGTAAGAATGTAASTHCTATCKGTTACAAAAAAAAAAAAAAAACAAAAAAAAAAAAACAC | CV013752,CV014498,CV014808,  CV014892, CV014613,CV066978 | YES | YES |
| 100 | TUG100 | GGGGGGGACTTGGAATCAGCTTCGTCAGTATGTGCAAAATCGTAGCCGCAAGGTGTGAATAGATTCTTGACAAGGTGTGCTAGACAATCTGTTGCTACCAAATTTTTTAATTTTTTAATTTTTTACTGTATGTATGTATGTATGTATGTATGCACTGATGCCAATGGTGGGTGACGGATGTTTAGGGATGTTTAACTTATGATAATGGAAGTATCTCCTTTCTTCTCCAAAAAGATAAAAACATATAAAAAGAAAAAAAAAAA | CV014589 | YES | N0 |
| 101 | TUG101 | GGGGGAGATTGGTATGGCATTGAATGATCAGAGCTTCCTCTTCTCCTTCCCCCAAAACCTAATCCAATCCAATCCAAGCCAAGCCAGGCCAAGCCAATCCAAGCACCACAATCGCCGCCGCCGCCGCCGCCGCCATACGAAGAAGATCAAACAGAGGAGGAGGGGTTCAGGGAAATGGGGGGATTGAAAAATGATGTACGTAAAGGCATCGCATCCACCTGATATGAATCGTAATACGGAGTGGTTCACGTATCCAGGGGTTGGGACCACCTACATCTTGATCCTCTTCTTCTCTGGCTCATCGTTCTCTCCCTATTCGGCTGCTCTCCCGGCATGGCCTGGACCATCGTCAATCTCTCCCACTTCGCCGTCACTTATCACTTTTTCCATTGGAAGAAAGGAACTCCGTTTGCTGACGACCAGGGGATCTATAATGGGCTGACTTGGTGGGAACAGATGGACCATGGAAAGCAGTTCACCCGCAACAGAAAGTTCCTCACTGTTGTACCTGTGGTGCTGTACTTGATAGCCTCACACACAACTGACTACCAACACCCGATGCTCTTCTTCAAACACTCTTGCAGTGGTTGGTGCTCGTTGTTGCCACAATTCCCCCAAATAATGCACACGGTCCCGATCTTCGCACTAAAGTGGAGAAGCCA | CV014485 | YES | N0 |
| 102 | TUG102 | GGGGGTCGTAGCTCGCACACAACACCACCAACAAGAGAGAAGAAGGGGGGGGACAACAAAAAAAAATTTACCCTTTAAAGGAAAGGAAGGAAAGGGAAAAGGAAAGGAAAGGAAAGGAAGGGAAAGGAAAGGAAAAACCGGGGGAAAGGGGGAAACCAAGGGCGTCGTCATTTCGGAGGGGACGGATCTCATATCGTGTGTGTGGAGAGAGAGAGAGAGAGAGAGAGTGAATATTAGAAAGAGAAAGGGAGACGCAGAGAGTGTGTCGTTGGAGAAGAGAGAGAGAAGCATCTGGAATCTGTTTGATTTGTGACTGGAGCATTTTGATTGTTCTAGAAGGAAGGAAGAATGGAGAGAAAAGGCACTTCCGGTGAGGAAGTCTCACACATCCACTGCAGATCTACTCACCTGGTCTGAAATTCCTCCCTCCTATTCCCCCTCCACCGCCTCAGCTGCTCG | CV013805 | YES | YES |
| 103 | TUG103 | GGGGATTGGTTCTTAGCTTTGTGGAGCCTGAGTTAGGAGAGTGAGAGAGAGAAGAGAAGAGAAGAGAAGAGAGTTTAGCTGTAGTGAGATGGCTTCATTGAACATATTCTTTTCGCCAGCAGTGACCACCGGAAGAGTGTATGCTGCGACAGCAGCCAAGAGCTCCGGCGGAAGTGAGGAGAAGAGCCTCCTGGACTTCATTCTTGGGGGGTTASCCGAAAGGAAMGATCAMGATGCTGGAGACCGACCCCATCCTGAAGAAGGTGGAGGGAGAAGAATGGCGGCCGCAAGAATGTTGTGGCGGTTCCTTCCAAGRAAGAAGAATGGTGGCTTTTGGAGGCTTTAGGAGGCCTTTTCGACAAGAAATGAATCTACTGAATGACACCCCAAGTTTCTCTCTCTCTCTCTCGCTACTGAATGAATGAATCTACTTTCAAGCGTAGATGATTCATCTTCAAATGCTAAGTTGTGTTTTCATTTTCTTATTTATGAACTGATATATATTTATTACGAATAATCAATTCATTTGGCAATCTGGACCCGTGCCACTAAGTATTAATGCTGTAGCATTGTTGTACASRAAAMWAMWRMRRWRWAMMWAAAAMWACCGCCCATAATTATCAAATAATTTCTCTTCATGCCGAAAAA | CV013769,CV067084,CV014255 | YES | NO |
| 104 | TUG104 | GGGGGACGGTGTGTTCTATCTCGCTCTCGCTCTGGCTCGCTGCGCTGCGCTGCGCTGACTCTCCCTCAAACAGAGATATTTTGCATAGCAGTTGGTCCTACATGGCTTCGTTCCGAGCATTCTTGGAACAGTCCAGTTGGCCCTAAAACAACTCATTTCTTGGGGTCCTGTTGCTAACTGGGGATTCGTAATTGCTGGGCTGGTGGACATGAAGAAACCCCCTGAAATGATCTCAGGCAACATGACTGGAGCAATGTGTGTATATTCTCTATTATTCATGAGGTTTGCATGGATGGTACAGCCTCGCAATTATCTACTTCTAGCATGCCATGCCTCGAATGAGACAGTGCAGCTCTATCAACTCTCTCGCTGGGCAAAGGGTAATGGGTACTTGGGTGGGCAGAAGAAGGATGAACCTGCACCTCAGTAACTTGGTGCTGCCCTTTTTCTTTTCCATTGTTGGGCCTGCTTCCCTACAGAAAACAAGTTGTCATTGATGTGAAATTAAGCATTTTATTAGTATGTGATTAGGGTACTAGCAGAACCACACAATCATTTCAAGTAAATTGTGACTGCTGTATTTGTGTATTGCACTCTGGCTTTGTTACAATCCAATATTACCTAAATAAACTGTTTGTCAGAATATTCAGCTC | CV013739 | YES | NO |
| 105 | TUG105 | GGGGGAGCTAGGGTTTTAGTTTCAGTAGTATAACTCAGAACGACTCTCTTCTCTTCTCTTCTCTTCTCTGCAAAGAAAAAATAACAGAAAGAAAGACGAGGCAAGGATGTTTCCGGGAATGTTCGCTCGGAAACCGGACAAAGAAGTGGCTCTGAAGCAGCTTCGCAGCCACGTAGCCATGTTCGGTGTTTGGGTCACCGTGGTTCGGATCACCCCTTACGTTCTCCACTACTTCTCTGATCGCAATGAACAACTCAACCTCGACTTCTAATCCTCCTCCTCCTTCTTCACATCTCACTCTTTTCGAGTAAGCCCTAAAAAAGCACACATTCCATGTCCCTACCTGACAAAACCATGTTTTAAATTAAATTTTTTAGCTCATACATTATATAAACATGTCCTTGATGTATCTGTATACTATATGTTTTTAAAATTTTACAGACATATTTGTAATAAA | CV013666 | YES | YES |
| 106 | TUG106 | TAGCGTGGTCGCGGCCGAGGTACTTCCTTGCCTTCTCACTCACAAGAAGCCCGTGATCATCGTGTCCCTCACTCTTTGCATGTTTCCATACACCTTTATAATCCTCTTTCTCAGAGACGATCCAGCTCCCCTCAAACGAATCATCAAACGAATCGTAAAAGATCGGATCAGAAGAAGCACAGAGCTGAGAAGTGAAGCAACCGATCAAAATCAACAAACTAAATTGCAGATACAACCTCCGATTACTTTCTTCCATCATCAAAATCAACTGATTCTGATAAAAAAAGAAAAAGAAAAAGTGCAAGATCTGAGATCTACAGCACTGTGAATCGTCGAACCCTAACCCTAACCCTAACCCTAACCACCGCTAACCTGCCCCGGGCGGCCGC | CV699835 | N0 | N0 |
| 107 | TUG107 | GGGGGACACAACCAACTCTTAGAAAAAACATCCCTTACTCTTGGAGATGGAGATGTATCTTCCAATCCTCTTCGTCGCTTCGCTTCTCTTCTCCTACTCTCATGGTTCAGTGCTAGACTTCTGTGTGGCAGACTTCTCATTGCCCGATGGACCTGCAGGCTACTCTTGCAAGAAACCTGCTAAAGTGACGGTTAATGATTTKGTTTATTCAGCTTTAGGCATTGCTGGTGACACTAACAATCTTATCAAAGCTAAGGTTACACCAGCATTTGCAGCTCAATTTCCGGGTGTGAATGGGCTTGGCCTTTCCATGGCACGTCTAGATCTAGCCGTGGATGGAGTTGTGCCAATCCACACGCACCCTGGAGGTTCAGAAATCCTTTTGTGGTGGAAGGAACAATTTGTGCCGGATTCATATCTTCTTCGGCCAACACTGTTTATTTTAAAACTCTTTACAAAGGGGATGTTATGGTTTTCCCACAAGGGTTATTGCATTTTCAATTCAATTCTGGAAATAAACCGGCTCTTGCGATTGTTAGCTTCAGTAGCCCAAGCCCGGGTCTCCAAATCACCGATTTTGCGTTGTTTGCCAACAATTTGCCTTCTGCATTGGTGGAGAAGGTAACTTTTCCTTGATGATGCTCAGGTCAAGAAAGCTCAAGGCTGTTCTTGGTGGCACTGGTTAAAAAAGGCCTTTGAAGCCTTTTTATTTTTATTTTTATTTTTTAWTTTTTATTATAATGGTTTMTTTTTGTAGTACTACTTAAAGRGTTMYTYYWGTCGAGTRTCTTTTGTATGGGTCTTTAACATTTCCTTCTGCAGGCAAATGCATGTTTTTTTTTCTTTTAATAGTATTTATCTGAATAATTGGAGAAATTCCATTAGTATTATTAGTATTATACCTTTCATATTATTTTGGTCCCTGCACTTTTATTCGATTTGAAAAAAAAAAAAAAAA | CV699727 | YES | NO |
| 108 | TUG108 | GGGGACATCATCACCAGCTTAAAAACTCAGCACAACAAAAAGCAAAGACACAAAAAAMCCAAAAACAAAAACACAARAACAAAAACATGTCTCTGATCACAGAAGAGGCCAAAGCAAAAGCAGAGTTCTACCAAGGAARCGACCTATGCCAAGAGAAATCAAAGGTCTTGCTCAAAGAATTGGGTTTGCCCAACGGTCTCCTYCCATTGGAGGACATGGAAGAGTGTGGCTRTGTCAGAGAAACCGGTTTATGTGTGGCTTAAACAGAAGAGGAAGACCGAGCACAAGTTCGAGAAGATTGGCAAACTCGTGTCGTATGCGACCCGAGGTCACTGCCTATGTCGAGCCCAAATAAGATCMAGAAGCTTGACCCGGTGTCAAGACTAAGGAGCTACTTGCTTTGGATCACTCTCAGYGATATATATKTCGATGATCCGCCGACCGGGAAGATCACTTTTTCAGGATTCCGGCTGGGCTGTCCMGGACTTTATCCCGTGTCGGCTTTCCGARGTGGCAAGASGGGCCGGCGAAGGCGGAGGTTGGTGGTGGAAATGTTAGTGTTACTGCTGCTGCTGTTGCTGC | CV014280,CV013736 | YES | YES |
| 109 | TUG109 | GGGCTCCCTCGGGCCACTCCTAAGAGCGGCGCCGCTGTTAACAGAGTCGCCGTTACCGGATCCGCCGCCGTTGTATTCTTCGCCGCAGCAACATGTTGCTTTAGATCTGATATCTGGAGTTGTTTTCTTCATTTGATCGAGATGAAGATTCTTTAGGATTTATTTTTTAGATTTTAATTTTAATTTTAATTTTCTTTTTTATTTCATTGCGGGGTGTTTGATGTTGTTATTATTATTATTATTATGATTACATTTGTCTGATTTACACTTGATTTGATTATTTGTATTGTATTGTTGATTCTGATTATGAATTCTTTTGCTACGATTTCTTTCCCAAAAAAAAAAAAAAAAAAAAAAAAAAAAAAA | CV014026 | N0 | N0 |

* Non redundant nucleotide sequence with clustering of random ESTs using SeqMan DNA Star lasvergene version 7.1.
